# Application of OU processes to modelling temporal dynamics of the human microbiome, and calculating optimal sampling schemes

**DOI:** 10.1186/s12859-020-03747-4

**Published:** 2020-10-12

**Authors:** Toby Kenney, Junqiu Gao, Hong Gu

**Affiliations:** Department of Mathematics and Statistics, Dalhousie University, Halifax, NS Canada

**Keywords:** Mean reversion, Time series, Sampling frequency, Ornstein–Uhlenbeck process, Fisher information

## Abstract

**Background:**

The vast majority of microbiome research so far has focused on the structure of the microbiome at a single time-point. There have been several studies that measure the microbiome from a particular environment over time. A few models have been developed by extending time series models to accomodate specific features in microbiome data to address questions of stability and interactions of the microbime time series. Most research has observed the stability and mean reversion for some microbiomes. However, little has been done to study the mean reversion rates of these stable microbes and how sampling frequencies are related to such conclusions. In this paper, we begin to rectify this situation. We analyse two widely studied microbial time series data sets on four healthy individuals. We choose to study healthy individuals because we are interested in the baseline temporal dynamics of the microbiome.

**Results:**

For this analysis, we focus on the temporal dynamics of individual genera, absorbing all interactions in a stochastic term. We use a simple stochastic differential equation model to assess the following three questions. (1) Does the microbiome exhibit temporal continuity? (2) Does the microbiome have a stable state? (3) To better understand the temporal dynamics, how frequently should data be sampled in future studies? We find that a simple Ornstein–Uhlenbeck model which incorporates both temporal continuity and reversion to a stable state fits the data for almost every genus better than a Brownian motion model that contains only temporal continuity. The Ornstein–Uhlenbeck model also fits the data better than modelling separate time points as independent. Under the Ornstein–Uhlenbeck model, we calculate the variance of the estimated mean reversion rate (the speed with which each genus returns to its stable state). Based on this calculation, we are able to determine the optimal sample schemes for studying temporal dynamics.

**Conclusions:**

There is evidence of temporal continuity for most genera; there is clear evidence of a stable state; and the optimal sampling frequency for studying temporal dynamics is in the range of one sample every 0.8–3.2 days.

## Background

A significant number of microscopic organisms live in and around the human body. Research has shown that the human microbiome plays a significant role in human health, for example [1–4]. Technological development in DNA sequencing has permitted a more systematic study of the microbiome [5].

Several recent studies have suggested the temporal dynamics of the microbiome may have clinical relevance to IBD. For example, Pascal et al. [6] found that under some $\beta$-diversity measures, there was more variation between multiple gut microbiome samples from individuals with Crohn’s disease than healthy controls, and there was less variation in samples from individuals with ulcerative colitis. This suggests that the dynamics of the microbiome may be affected by these diseases. However, that study was based on samples taken at 3-month intervals, so the actual dynamics were not observed. Other studies such as [7] have also sampled IBD patients and healthy controls at 3-month intervals. Vázquez-Baeza et al. [8] attempted to apply this to improve classification of IBD patients and healthy controls from microbiome data. By comparing longitudinal samples, they were able to improve classification accuracy. They argued that this might be caused by IBD patients having more variable microbiomes. However, it could also be explained by the fact that sampling more replicates would in general improve prediction.

Other studies have looked at the temporal dynamics of the microbiome when an individual’s diet changes. Wu et al. [9] and David et al. [10] both took daily samples of individuals in a controlled feeding experiment. They both found that the microbiome reacts quickly to sudden changes in diet, and reverts to baseline when the controlled diet regime ends. This suggests that the temporal dynamics of the microbiome should be measured on a scale of days, rather than months.

There have been a few methods developed by extending time series models to accomodate specific features in microbiome data to address questions of stability and interactions of the microbime time series, including the dynamic linear models (MALLARDs) [11] with analysis performed on artificial human gut datasets, the sparse vector autoregression (sVAR) model [12], ARIMA Poisson model [13] and the Linear Mixed Model (MTV-LMM) [14]. Most of these models assume equal space sampling (which is usually not the case for the real data) based on a discrete process and most of the papers did not directly address the sampling frequency issues. The MALLARD model used in [11] does deal with missing data. It is in theory possible to incorporate other irregular sampling frequencies into the MALLARD framework, though it is not clear how much computational work is needed to reparametrise the model in such a way. The hierarchical nature of these models makes it difficult to study sampling frequency issues.

Since the microbiome is often considered as an ecological system, it is natural to model its temporal dynamics as a stochastic process. The observed stability suggests that a mean-reverting process may be appropriate. In this paper, we compare a mean-reverting process model with two alternative models: one alternative model is Brownian motion, which can be characterised as random drift; the second alternative model is an independent model, where observations at different time points are independent. By comparing with these models, we formally test the widely held beliefs that the microbiome does show some temporal continuity, and that the system is subject to mean reversion, meaning that the system returns to its stable state whenever the composition randomly fluctuates away from that state. After confirming these aspects of the dynamics, we will also obtain an estimate of the time-scale under which the dynamics operate. For this paper, we focus on the dynamics of each individual genus without explicitely modelling the interactions between different genera (part of the interaction is implicitly expressed by the mean reversion and another part is absorbed in the stochastic term in the model).

The Ornstein–Uhlenbeck model has previously been applied to microbiome data by Laitinen and Lahti [15] and Lloyd-Price et al. [16]. Lloyd-Price et al. [16] used a Bayesian hierarchical OU model to estimate the dynamics from a longitudinal data set with only several samples from each individual and the separation between samples between one month and one year. Our analysis therefore promises to give different information from theirs in several respects. Firstly, their model assumed that the parameters of the OU process were the same for different individuals. If the rates of mean reversion were not the same for all individuals, then this assumption could lead to incorrect estimates. Secondly, the data they analysed had time intervals in months, so could not identify the temporal dynamics on a daily scale. Thirdly, because there were only several samples for each individual, there was a limit to how much of the temporal dynamics could be estimated. Laitinen and Lahti [15] suggested the use of an OU process for modelling OTU abundance, and suggested that using a hierarchical Bayesian framework could improve estimates in cases where the rates of mean reversion were similar between different individuals. However, they only studied a single genus, Bacteroides, so it is hard to draw any general conclusions from their paper. Furthermore, the pre-supposition that the rates of mean reversion are comparable for different individuals needs to be assessed before it can be used as the basis for a model. We therefore model the data from different individuals separately, to see whether there is evidence that the mean-reversion rates are similar across individuals and body sites.

Another major issue in studying the temporal dynamics of the microbiome is sampling frequency. Sampling too frequently may result in not covering enough time to observe the patterns, while large gaps between samples can lead to consecutive samples being uncorrelated. It is widely acknowledged that “An important question is how often to sample …” [17]. Current knowledge on this topic largely consists of guesswork, based on what has been observed in studies conducted at different timescales. However, stochastic differential equation models, in addition to offering insightful explanations of the dynamics, also allow us to apply the powerful statistical theory of Fisher information. This theory provides the asymptotic variance of parameter estimates, based on the sampling scheme and the true parameter values. It is then a straightforward optimisation problem to determine which sampling scheme will generate the most accurate estimates of the temporal dynamics.

In this paper, we study the moving picture data set [18] and the David et al. data set [19]. These two published time series studies contain long time series measurements, with approximately daily sampling, for four healthy individuals in total. We are interested in healthy individuals because we want to better understand baseline temporal dynamics of the microbiome. This will help to interpret future work on how the dynamics change under certain conditions. For these data sets, we estimate the temporal dynamics of the most abundant genera in each environment. We perform formal tests between the OU model and both Brownian motion without drift and i.i.d. normal data. We study the estimated rates of mean reversion for each abundant genus in each dataset, and examine these estimates for any patterns. We also apply our method to calculate the variance of the mean reversion rate estimates for these datasets. Finally, we use the Fisher information based on the estimated dynamics from these datasets to determine the optimal sampling frequency for future studies. This is, to our knowledge, the first application of Fisher Information to sampling design in temporal microbiome studies.

## Results

### Summary of the two data sets studied

We performed this analysis on two real-world data sets—the moving picture data set [18] and the David et al. dataset [19]. These data sets each followed two healthy individuals over 6-month to 15-month periods. In the moving picture dataset, four body sites were observed: gut, tongue, right palm and left palm, while in the David et al. data, most samples observed the gut (we did not analayse the tongue samples from this data set as they were only available for one individual). Samples were not collected at completely regular time intervals. Many samples were taken at daily intervals, but many intervals of multiple days were also present. For the David et al. data, there were some days with multiple samples. These were amalgamated into a single sample. Samples in the moving picture dataset were sequenced using PCR on the V2 region of the 16S rRNA gene [5], while samples in the David et al. dataset were sequenced using PCR on the V4 region of the 16S rRNA gene. We aggregated the data at genus level and normalised counts by dividing by total read count for that sample. We then restricted our attention to abundant genera with total counts greater than 20,000 over all samples in a given environment (e.g. Person 1’s gut) in the moving picture dataset, and genera with total count over all samples for one subject exceeding 10,000 and average proportion for that subject greater than 0.005 for the David et al. data. There are several reasons for restricting attention to the most abundant genera. From a biological point of view, the most abundant genera often have the largest effects on the behaviour of the community, so studying their dynamics is more important. From a technical point of view, our analysis has not made any correction for the fact that we record a discrete count for each abundance, rather than a continuous abundance measurement. Approximating the discrete counts as continuous proportions is more accurate for abundant genera, so our model is expected to better fit the data for abundant genera. Furthermore, because we are interested in the log-scale dynamics of the microbiome, we cannot directly deal with zeros in the data, and need to implement an ad-hoc approach for this. For abundant genera, this has little effect because there are few zeros in the data. Finally zero counts were replaced with a pseudocount of 0.3 to enable log-scale analyses.

Tables 1 and 2 show the number of observations and abundant genera for each individual and each body site respectively in each dataset.

**Table 1:** The number of observations for each individual and body site

(a) Moving picture					(b) David et al.	
	Gut	Tongue	Right palm	Left palm		Gut
Person 1	131	135	134	134	Subject A	314
Person 2	336	373	359	365	Subject B	183

**Table 2:** The number of abundant genera for each individual and body site

(a) Moving picture					(b) David et al.	
	Gut	Tongue	Right palm	Left palm		Gut
Person 1	17	12	10	36	Subject A	25
Person 2	39	23	80	35	Subject B	19

### Sequencing depth and compositionality

One major issue with microbiome data is that total read counts are affected by many technical factors not related to the original sample, so the total count from a sample is at best very weakly related to the original microbial abundance, while the relative abundances are believed to be much more strongly related to the relative abundances in the original environment. Therefore, it is common to attempt to correct for this in some way that focuses on the observed relative abundances. The simplest way is to analyse the proportion of each OTU in a given sample, instead of the count of the OTU. This has the undesirable consequence of inducing negative correlations between all genera. This is particularly problematic for analyses involving pairwise correlations between genera [20], and differential abundance of OTUs [21], but it could also create problems for our anaylsis. If the true abundance of one genus undergoes mean reversion, then because of the sum-to-one constraint on proportions, other genera may appear to show the same mean reversion, even if their true abundance is not subject to mean reversion. Other approaches have been used for handling the compositionality, such as centred log-ratios (CLR), but this does not solve the main problem that the relative abundance of a genus depends on the abundance of other genera. Furthermore, compositional data analysis methods are based on strictly positive data and “... the problem of zeros is unlikely ever to be satisfactorily resolved.” [22]. In particular, since centred log-ratios involve taking logarithms of counts of all genera, they are more sensitive than proportions to the choice of methods for dealing with zero counts. Methods such as multiple imputation as in [23] may help to deal with this uncertainty. However there is a heavy computational cost.

An approach that does deal with the issue of the abundance of a single genus affecting the relative abundances of all other genera is pairwise log-ratios. The ratio between the abundances of two genera is not affected by the sequencing depth, so does not need to be corrected. This is used in the ANCOM method [24] for testing differential abundance of microbes. Because the pairwise log ratio of two relative abundances is the same as the pairwise log ratio of their absolute abundances, it is not affected by the abundance of any other genera. Furthermore, recent evidence, such as [25] suggests that microbiome data are subject to multiplicative noise, where the multiplicative factor depends on the particular genus. For a fixed pairwise log-ratio, this bias becomes an additive constant, so does not affect the estimated mean reversion rate. However, pairwise log-ratios are more difficult to interpret, and because there are many more pairwise log ratios, more computation is needed to analyse the pairwise log ratios.

We performed simulations to assess the effect of these normalisation methods and the effect of the sampling—the observed data are counts, rather than continuous measurements of microbial abundance. In our simulations, the true abundance of each genus follows a certain model, and we test the null hypothesis that the normalised abundance follows this model as a proxy for the true null hypothesis. Full details of the simulations are in Additional file 1: Appendix C. In summary, we found that:

- Without Poisson noise, pairwise log-ratios have false positive rates close to or below the nominal size of the test.
- With Poisson noise, all methods have false positive rates above the nominal size of the test. For pairwise log-ratios, the false positive rates are mainly in the range 10–35%, so not too large.
- For log-proportion, the false positive rate is in the range 10–30% for cases with Poisson noise and in the range 40–45% in the case without Poisson noise where some genera follow Brownian motion and others follow an OU process.
- CLR has much higher false positive rates than other methods.
- Even in cases where the tests give a false positive, the estimated mean reversion rate is very small.
- The mean reversion rate of one genus can affect the estimates for other genera using log-proportion or CLR.
- This effect is more significant for abundant genera using log-proportion and more significant for less abundant genera using CLR.

Based on these results, pairwise log-ratios give the most reliable results, so we analysed these for the real data. For improving interpretability, it is still helpful to use a method that analyses a single genus, rather than a ratio of two genera. Based on our simulations, log-proportions are better for this purpose than CLR, particularly as we expect most genera to reject Brownian motion, so controlling the false positive rate makes these rejections more reliable. We therefore also analysed log-proportion data.

### Testing temporal dependence of microbial dynamics

We performed a likelihood ratio test with null hypothesis that the log-proportion of a given genus (or the pairwise log-ratio between the abundance of two genera) follows an i.i.d. normal model, where there is no temporal dependence between observations, and alternative hypothesis that it follows an Ornstein Uhlenbeck (OU) process which includes temporal dependence. From the results in Additional file 1: Appendix C, this hypothesis is a reasonable proxy for an hypothesis that the log-abundance of the genus follows an i.i.d. normal distribution. The number of genera rejecting the null hypothesis, at the 5% significance level, for each person and body site is shown in Table 3. Many of the abundant genera show strong evidence of dependence between different time points, particularly in more enclosed body sites, such as the gut. More exposed body sites show less evidence of temporal dependence. Since the i.i.d. normal model is a limiting case of the OU process when the rate of mean reversion tends to $\infty$, evidence of temporal dependence will be weaker in cases where the mean reversion is faster. It makes intuitive sense that exposed body sites could have faster mean reversion, because exposure to external influences is one of the driving factors that influence the microbiome towards its stable state, so body sites which are more exposed to external influences could be expected to exhibit faster mean reversion.

The likelihood ratios for each genus, along with the null distribution for each data set, are shown in Additional file 1: Figure 1 in Appendix D. Many of the likelihood ratios are much larger than the critical values, indicating very significant evidence of temporal dependence for at least some genera. The results for pairwise log-ratios are in line with the results for log proportions, rejecting the majority of cases.

**Table 3:** The number of abundant genera which reject the null hypothesis of i.i.d. log-normal distribution at the 5% significance level against an OU process for each individual and body site

(a) Moving picture					
		Gut	Tongue	Right palm	Left palm
Log proportion	Person 1	17/17	9/12	3/10	24/36
	Person 2	36/39	23/23	67/80	30/35
Pairwise Log ratio	Person 1	114/136	52/66	33/45	444/630
	Person 2	713/741	251/253	2967/3160	571/595

(b) David et al.					
	Subject A	Subject B			
Log proportion	24/25	17/19			
Pairwise log ratio	295/300	139/171			

Numbers after “/” are total number of abundant genera

### Testing for mean reversion

We tested for mean reversion using a likelihood ratio test between a null hypothesis of Brownian motion without drift, which has no mean reversion and an alternative hypothesis of an OU process, where the rate of mean reversion is given by the parameter $\eta$. The likelihood ratio statistics for each abundant genus in each body site are shown in Additional file 1: Figure 2 in Appendix D along with the null distribution and critical values. In addition to the statistical benefits of comparing nested models, setting the drift parameter to 0 in a Brownian motion is natural because the proportions of different genera are constrained to lie between 0 and 1, so a model with drift is not sustainable. For pairwise log-ratios, a Brownian motion with drift is sustainable only when the abundance of one genus goes to zero. Nearly all the log-likelihood ratio tests reject the null hypothesis at the 5% significance level. The largest *p* value of any genus in each body site for each person are shown in Table 4. There is very strong evidence rejecting Brownian motion at the 5% significance level in almost all data sets. The only case where Brownian motion was not rejected is the pairwise log-ratio between *Bilophila* and an unidentified genus from family Alcaligenaceae in the moving picture Right palm data. This unidentified genus from family Alcaligenaceae also gave the largest *p* value for the log-proportion data. Examining the abundance of this genus over time, we see that it was almost not observed before day 97, then it was continually present in the samples. Analysing only the data from day 97 onward, we found that the *p* values for the log-proportion and for the log-ratio with *Bilophila* were both $<0.0002$. We have therefore found strong evidence that all abundant genera are subject to some mean reversion.

**Table 4:** Largest *p* values for any genus in each data set for a likelihood ratio test between Brownian motion without drift, and an OU process

(a) Moving picture				
Person	Gut	Tongue	Right palm	Left palm
1	0.0064	< 0.0002	< 0.0002	< 0.0002
2	< 0.0002	0.0018	0.002	< 0.0002
Person 1 pairwise log ratio	< 0.0002	< 0.0002	0.0002	< 0.0002
Person 2 pairwise log ratio	< 0.0002	< 0.0002	0.0684	< 0.0002

(b) David et al.				
	Subject A	Subject B		
Log proportion	0.0002	0.001		
Pairwise log ratio	0.0006	0.0054		

These *p* values were calculated using a simulation of 5000 values from the null distribution (Brownian motion without drift)

Mean reversion is expected, since we know there are many mechanisms that keep the microbial system in a stable state. The likelihood ratio statistics between the OU process and Brownian motion are larger on average for the palms than for the gut and the tongue, while the likelihood ratio statistics for the comparison with the i.i.d. log-normal distribution are larger for the gut and the tongue. This suggests stronger mean reversion in the palm microbiomes, and weaker mean-reversion in the gut and tongue microbiomes, which can be explained by the fact that the gut and tongue are enclosed systems with fewer external influences driving the microbiome back to the stable state.

### Disruptions to microbiome in David et al.

In the data from David et al., there were external disruptions to the microbiome noted for both individuals. Subject A travelled several times and had two bouts of diarrhoeia. Subject B experienced food poisoning for a period of time during the study. These events had a clear effect on the microbiomes of the subjects. In theory under the OU model, these disruptions should be followed by the normal mean reversion process, so should not greatly affect our estimates of the temporal dynamics. However, the OU model does not perfectly model the temporal dynamics, so it is possible that our estimates were affected by these disruptions. Table 5 compares the estimated ${\hat{\eta }}$ values for various time periods.

**Table 5:** Estimates of ${\hat{\eta }}$ over different time periods for the David et al. data

(a) Subject A						
Phylum	Class	Family	Genus	All data	Days 123–364	
Actinobacteria	Actinobacteria	Actinomycetaceae	Actinomyces	0.832	0.6665	
Actinobacteria	Actinobacteria	Bifidobacteriaceae	Bifidobacterium	0.3566	0.5527	
Bacteroidetes	Bacteroidia	Bacteroidaceae	Bacteroides	1.441	1.2507	
Bacteroidetes	Bacteroidia	Prevotellaceae	Prevotella	2.0256	1.9338	
Firmicutes	Bacilli	Gemellaceae		1.1221	1.3865	
Firmicutes	Bacilli	Carnobacteriaceae	Granulicatella	1.6666	1.8486	
Firmicutes	Bacilli	Streptococcaceae	Streptococcus	1.3472	1.5587	
Firmicutes	Clostridia	Lachnospiraceae		0.136	*0*.*5283*	
Firmicutes	Clostridia	Lachnospiraceae		0.2726	*0*.*8016*	
Firmicutes	Clostridia	Lachnospiraceae		0.5886	*1*.*0314*	
Firmicutes	Clostridia	Lachnospiraceae	Blautia	0.7966	0.785	
Firmicutes	Clostridia	Lachnospiraceae	Coprococcus	0.7619	0.7905	
Firmicutes	Clostridia	Lachnospiraceae	Dorea	0.8493	0.9004	
Firmicutes	Clostridia	Lachnospiraceae	Roseburia	0.1311	0.1414	
Firmicutes	Clostridia	Lachnospiraceae	[Ruminococcus]	0.5008	0.6914	
Firmicutes	Clostridia	Ruminococcaceae		0.1105	*0*.*4005*	
Firmicutes	Clostridia	Ruminococcaceae		0.6707	0.5662	
Firmicutes	Clostridia	Ruminococcaceae	Faecalibacterium	0.8978	0.8938	
Firmicutes	Clostridia	Ruminococcaceae	Ruminococcus	0.5539	0.7457	
Firmicutes	Clostridia	Veillonellaceae	Phascolarctobacterium	0.734	0.8391	
Firmicutes	Clostridia	Veillonellaceae	Veillonella	0.9389	0.9646	
Firmicutes	Erysipelotrichi	Erysipelotrichaceae		0.7742	0.8541	
Fusobacteria	Fusobacteria	Fusobacteriaceae	Fusobacterium	2.0122	1.6443	
Proteobacteria	Gammaproteobacteria	Enterobacteriaceae		0.3079	0.3638	
Proteobacteria	Gammaproteobacteria	Pasteurellaceae	Haemophilus	2.1665	2.6424	

(b) Subject B						
Phylum	Class	Family	Genus	All days	Days 0–150	Days 160–318
Actinobacteria	Actinobacteria	Bifidobacteriaceae	Bifidobacterium	0.9358	1.2257	1.2334
Bacteroidetes	Bacteroidia	Bacteroidaceae	Bacteroides	0.6007	0.8103	0.3681
Firmicutes	Bacilli	Streptococcaceae	Streptococcus	1.3091	*2*.*78*	0.785
Firmicutes	Clostridia	Lachnospiraceae		1.8332	0.6724	2.4082
Firmicutes	Clostridia	Lachnospiraceae		0.5128	0.9548	*2*.*2174*
Firmicutes	Clostridia	Lachnospiraceae		0.3539	*0*.*733*	0.3101
Firmicutes	Clostridia	Lachnospiraceae	[Ruminococcus]	0.139	*0*.*5218*	*1*.*4967*
Firmicutes	Clostridia	Lachnospiraceae	Blautia	1.0021	0.7035	0.3194
Firmicutes	Clostridia	Lachnospiraceae	Coprococcus	0.6375	0.706	*1.1748*
Firmicutes	Clostridia	Lachnospiraceae	Dorea	0.7622	0.7333	0.9792
Firmicutes	Clostridia	Lachnospiraceae	Roseburia	3.9203	1.4014	5.3445
Firmicutes	Clostridia	Ruminococcaceae		0.7842	0.6013	*5*.*3743*
Firmicutes	Clostridia	Ruminococcaceae	Faecalibacterium	0.7168	0.5899	1.0053
Firmicutes	Clostridia	Ruminococcaceae	Ruminococcus	0.2663	*0*.*6*	0.2664
Firmicutes	Clostridia	Veillonellaceae	Phascolarctobacterium	1.8373	1.2156	1.0925
Firmicutes	Clostridia	Veillonellaceae	Veillonella	1.4537	1.4415	2.2837
Firmicutes	Erysipelotrichi	Erysipelotrichaceae		0.5073	0.5104	0.6201
Proteobacteria	Gammaproteobacteria	Enterobacteriaceae		4.9281	3.9415	5.3409
Proteobacteria	Gammaproteobacteria	Pasteurellaceae	Haemophilus	1.2358	1.0513	2.0246

Estimates for shorter time periods that differ from the estimate for the whole data by more than 2 standard errors are highlighted in italics. Entries in underline indicate that the estimates were close to 2 standard errors from the whole data estimate

Most of the estimated mean reversion rates are consistent between different time points. However, for some of the genera, restricting to a shorter time period has a significant impact on the estimated mean reversion rate.

**Fig. 1:**
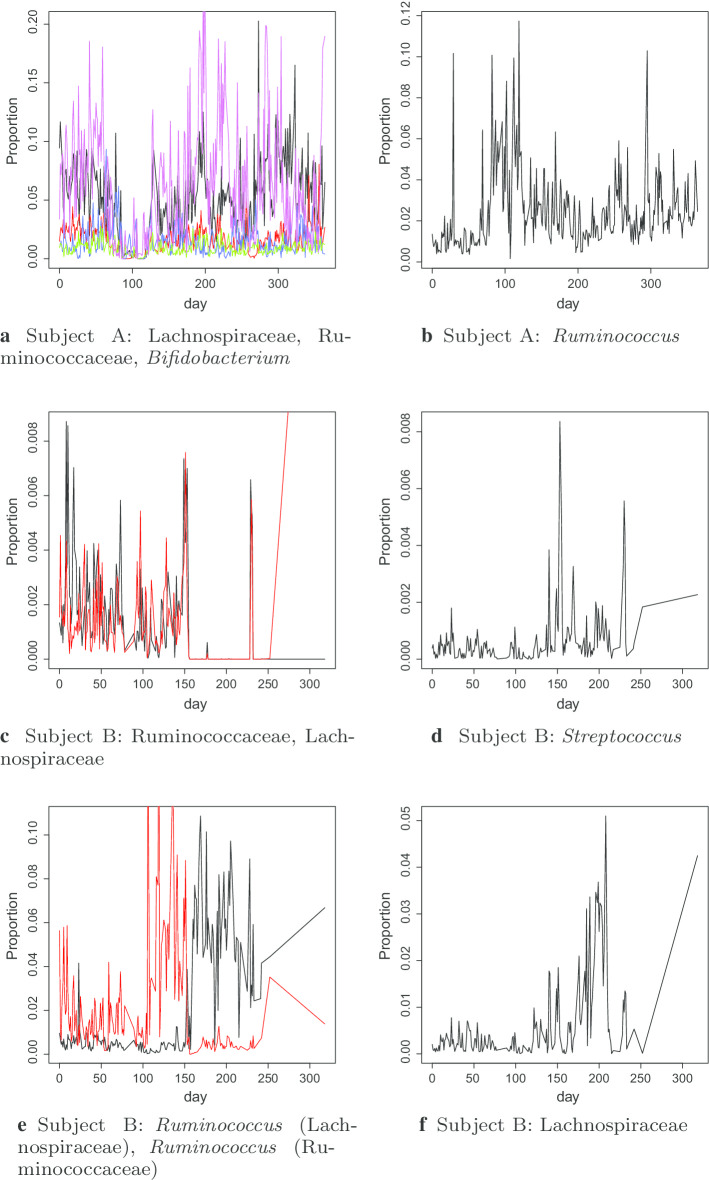
Relative abundance for which ${\hat{\eta }}$ estimates vary with time intervals in the David et al. data, over time

Figure 1 shows the relative abundance of most of the genera for which the results over different time intervals give inconsistent results.

A common pattern among these genera is a shift in the mean relative abundance. This makes sense, since the OU model only includes a single mean value towards which the abundance is always reverting. A shift in mean at some point will lead to underestimating the rate of mean reversion. One common shift is between presence and absence. This is natural since the absence of a particular genus is always a stable state.

We exclude the genera where the estimated ${\hat{\eta }}$ are significantly different for the time periods, namely *Bifidobacterium*, unidentified genera from family Lachnospiraceae, *Ruminococcus* (family Lachnospiraceae) and an unidentified genus from Ruminococcaceae, for Subject A; and *Streptococcus*, two unidentified genera from family Lachnospiraceae, *Ruminococcus* (family Lachnospiraceae), an unidentified genus from Ruminococcaceae and *Ruminococcus* for Subject B. We include *Coprococcus* for Subject B because the difference in ${\hat{\eta }}$ is only marginally significant, and there are no obvious shifts in mean.

**Fig. 2:**
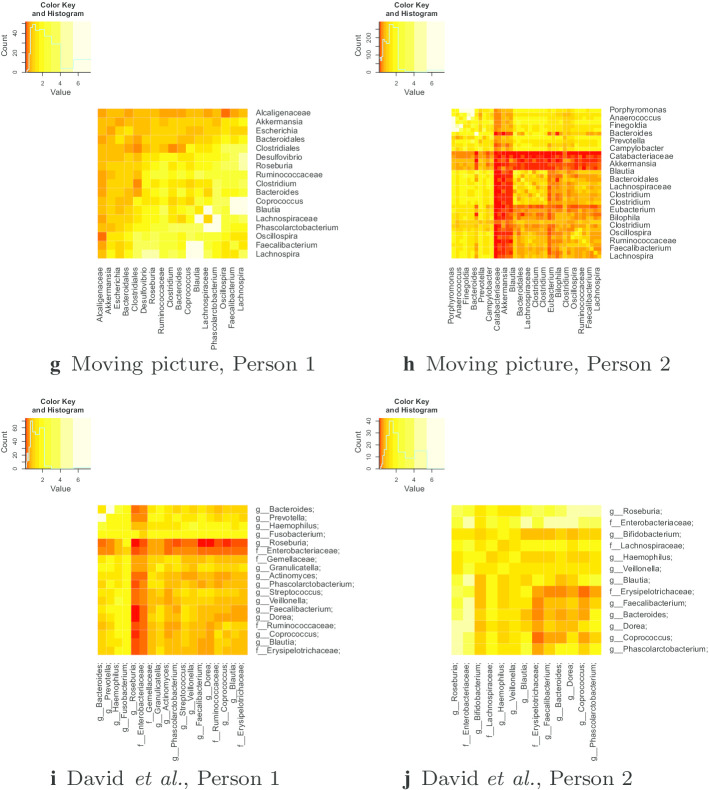
Comparison of estimated $\eta$ values for time series of pairwise log ratios between abundant genera. Diagonal entries in the heat-maps show the estimated $\eta$ values from the log-proportions of that genus

### Mean reversion rate estimates from pairwise log ratio and log proportion data

Figure 2 shows heatmaps of the estimated mean reversion rates of the pairwise log ratios of abundance for both the moving picture data and the data from David et al. The diagonal entries in these heatmaps are the estimated mean reversion rates for the log proportions of individual genera. The majority of mean reversion rates of the pairwise log-ratios are between the estimated $\eta$ values using the log proportion of the given genera, or within one or two standard errors, suggesting that the estimates from the log-proportion data reasonably reflect the rate of mean reversion of the genera.

For Person 1 from the moving picture data, there are 136 pairs of abundant genera, so results where the ${\hat{\eta }}$ from the pairwise log-ratio is within 3 standard errors of one of the ${\hat{\eta }}$ values estimated from the log-proportions, or between the two log-proportions are not surprising. There are only four pairs for which the estimated ${\hat{\eta }}$ from the pairwise log-ratio is not within 3 standard errors of one of the estimates from the log-proportions or between the two estimates from the log-proportions. All of them involve high estimates of ${\hat{\eta }}$, where the standard error is larger.

For Person 2 from the moving picture data, there are two groups of genera whose pairwise log-ratios show significantly different dynamics from the single-genus log-propotions. For the first group: *Parabacteroides*, *Lachnospira*, *Roseburia* and *Faecalibacterium*, the pairwise log-ratios have much lower estimates of ${\hat{\eta }}$ than the corresponding log-proportions. These genera are all positively correlated. For the second group: *Porphyromonas*, *Dialister*, *Peptoniphilus* and *Finegoldia*, the pairwise log-ratios have faster estimated mean reversion rates than the corresponding log-proportions. The large values correspond to cases where the log-ratio could not reject an i.i.d. normal hypothesis. The log-proportions of these genera are also highly positively correlated.

**Fig. 3:**
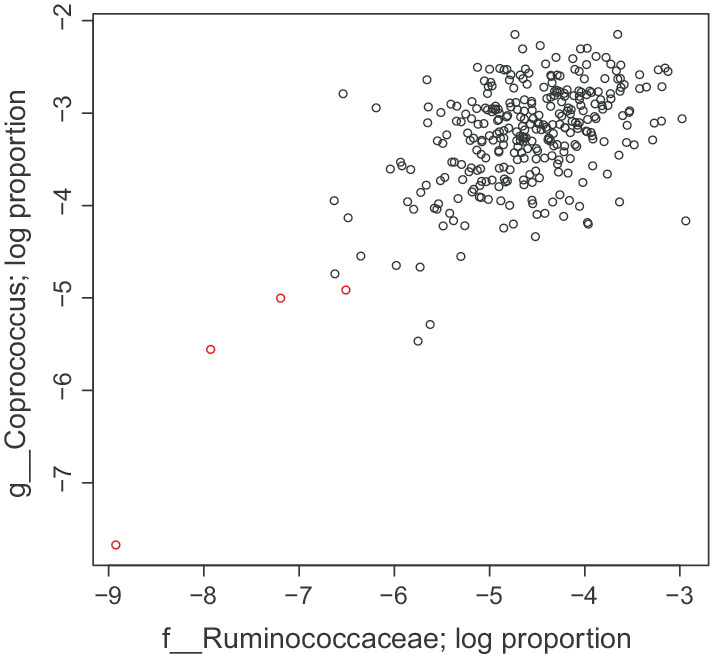
Log proportion of *Coprococcus* and a genus from Ruminococcaceae for David et al. Subject A

For the David et al. dataset, the rates of mean reversion for pairwise log ratios are mostly between the rates of mean reversion for the log proportions of the individual genera. For Subject A, There are only two pairs of genera for which the log ratio is more than 2.5 standard errors away from the interval between the corresponding log-proportions, namely *Coprococcus* and an unidentified genus from Ruminococcaceae and *Bacteroides-Prevotella*. For *Bacteroides-Prevotella*, the log-proportion data for *Prevotella* only marginally rejects the i.i.d. normal hypothesis, so the log-ratio being unable to reject the i.i.d. normal hypothesis is not very surprising, and does not suggest any artefacts of using log-proportion data. For the log-ratio between *Coprococcus* and the unidentified genus from Ruminococcaceae, it is not clear exactly what causes the difference between the dynamics of the log proportion data and the log ratio data. It might be explained by the significant tail dependence between these genera. This is demonstrated in Fig. 3. There is some weak dependence between the two genera, but it is most significant in the lower left tail, where some samples have very low abundance of both genera. However, removing these points does not have a large effect on the estimated mean reversion rates, so there must be some other factor causing the log-ratios and log propotions to give different estimated dynamics.

For Subject B, only several pairwise log-ratios show an estimated mean reversion rate far outside the mean reversion rates for the log proportions of the individual genera. These involve the genera *Blautia*, *Coprococcus*, *Dorea* and a genus from Erysipelotricheae. These genera are also relatively low abundance, so it is unlikely that the behaviour of the pairwise log ratios is an artefact of compositionality.

Rates of mean reversion are comparable for log-ratios and log proportions in the vast majority of cases in all datasets. Even in the cases where they are not comparable, if the problems were caused by one or two abundant genera affecting the log-proportions of other genera, we would expect to see disagreements between log proportions and pairwise log ratios for most pairs not involving the most abundant genera. Since we do not observe any such pattern, we have no reason to mistrust the results of the log-proportion analysis. We therefore focus our analysis on the log-proportion results because these results are easier to interpret.

### Variance of estimated mean reversion rates and optimal sampling protocols

**Fig. 4:**
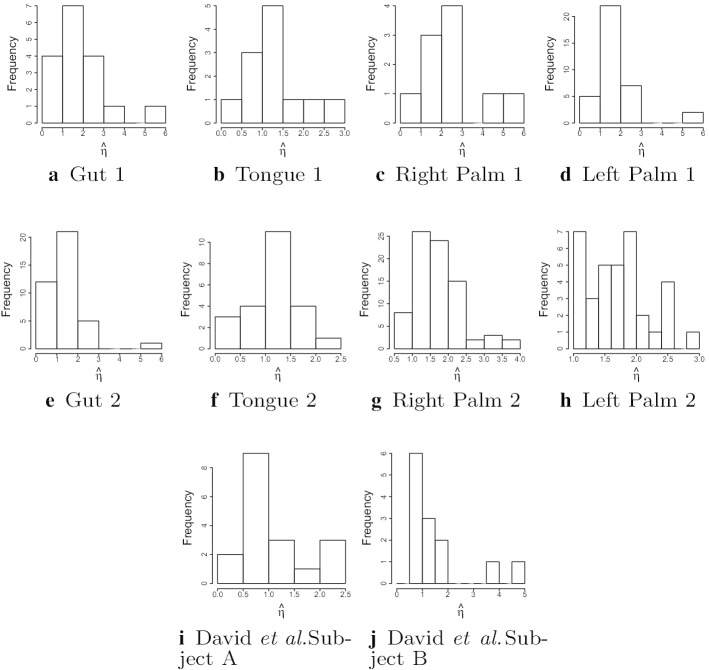
Distribution of ${\hat{\eta }}$ over genera for Person 1 (top), Person 2 (middle) from the moving picture dataset [18] and both subjects from David et al. [19] (bottom)

Under an OU process, based on the theory of Fisher information, the asymptotic covariance of the parameter estimates in the OU model is given by the inverse of the Fisher information matrix given in Proposition 1. The variance of ${\hat{\eta }}$ depends on $\eta$, but not on $\mu$ or $\sigma$. Histograms of the estimated values of $\eta$ are given in Fig. 4.

Many values of ${{\hat{\eta }}}$ are close to 1, with a few larger values. The standard deviations of ${\hat{\eta }}$ for several different values of $\eta$ are given in Table 6. In the "Methods" section, we are able to use the theory of Fisher information to determine the optimal sampling scheme for estimating the mean reversion rate $\eta$ from a fixed number of observations. We compare the estimated standard deviations using the actual sampling scheme with the standard deviations that could be achieved by the optimal sampling scheme with a similar number of samples.

**Table 6:** Estimated standard deviations of ${\hat{\eta }}$ for various data sets and true mean reversion rates

Study	Body site	$\eta =0.4$	$\eta =1$	$\eta =1.5$	$\eta =2$
Moving picture	Person 1 Gut	0.093985	0.229590	0.404363	0.682644
	Person 1 Tongue	0.093338	0.224076	0.392306	0.660776
	Person 1 L. palm	0.093542	0.225305	0.395091	0.665951
	Person 1 R. palm	0.093542	0.225305	0.395091	0.665951
	Optimal sampling 131 samples	0.086844	0.217093	0.325639	0.434185
	Optimal equal-spaced 131 samples	0.087189	0.217972	0.326959	0.435945
	Person 2 Gut	0.058055	0.143934	0.256814	0.436208
	Person 2 Tongue	0.056130	0.133687	0.234737	0.395993
	Person 2 L. palm	0.056856	0.137175	0.242194	0.409543
	Person 2 R. palm	0.056524	0.135688	0.239088	0.403997
	Optimal sampling 351 samples	0.052923	0.132307	0.198460	0.264614
	Optimal equal-spaced 351 samples	0.053137	0.132843	0.199265	0.265686
David et al.	Subject A	0.060678	0.146476	0.258630	0.437380
	Optimal sampling 314 samples	0.055963	0.139909	0.209863	0.279817
	Optimal equal-spaced 314 samples	0.056190	0.140476	0.210713	0.280951
	Subject B	0.078551	0.195710	0.347970	0.589763
	Optimal sampling 183 samples	0.073391	0.183477	0.275215	0.366953
	Optimal equal-spaced 183 samples	0.073688	0.184220	0.276330	0.368441

From Table 6, we see that ${\hat{\eta }}$ is a reasonable estimate for $\eta =0.4$ and $\eta =1$, with a coefficient of variation ranging from 20 to 25% for all body sites for Person 1 in the moving picture data to a coefficient of variation of about 14% for Person 2 in the moving picture data, with the coefficients of variation for the David et al. datasets between these extremes. The sampling scheme used achieves an accuracy close to the optimal sampling for genera where the true rate of mean reversion is in this range. For the genera with faster mean reversion, ${\hat{\eta }}$ is less accurate, and the accuracy could be improved by sampling more frequently.

**Fig. 5:**
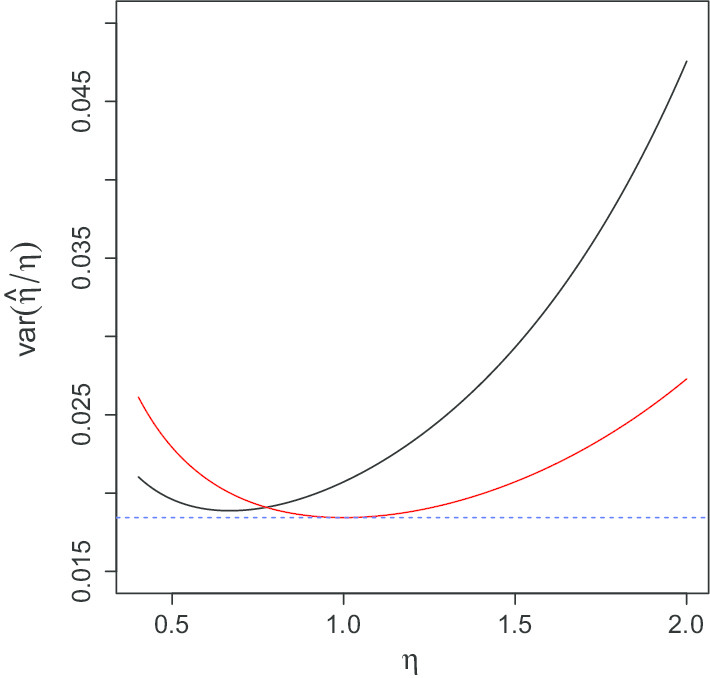
Variance of ${{\hat{\eta }}}$ under actual sampling (336 samples) for Person 2’s gut from the moving picture data (black), under a sampling scheme with 336 samples, optimised for $\eta =1$ (red), and under a sampling scheme with 336 samples, optimised for each $\eta$ separately (blue)

From Theorem 3 (see "Methods" section) we see that the optimal sampling scheme to study the temporal dynamics of a genus with mean reversion rate $\eta$ is to sample regularly with time step ${1.59362426}/{2\eta }$. Thus, for some of the less quickly reverting genera, it would be better to sample slightly less frequently, while for some of the more frequently reverting genera, we should aim to sample more frequently to understand the temporal dynamics. Some of the fastest mean-reverting genera have ${\hat{\eta }}$ more than 2. For this value of $\eta$, it would be best to sample 2.5 times per day. Obviously, this may be impractical for some environments. Figure 5 shows the effect of the sampling scheme on our estimates of $\eta$. We compare the actual sampling scheme, a sampling scheme optimised for $\eta =1$, and the best results that can be obtained for each particular $\eta$.

From Fig. 5, we see that the actual sampling gives fairly good estimates for smaller values of $\eta$, but for larger values of $\eta$, the variance of ${{\hat{\eta }}}$ is about twice as large as it would be if the sampling were optimised for $\eta =1$, and nearly three times as large as it could be with more frequent sampling. We see that optimising for $\eta =1$ would be good at controlling the variance for most of the values of $\eta$ estimated from the data. We conclude that daily sampling is fairly good, and should be used for future studies. Ideally, for the rates observed here, we should aim to sample slightly more frequently than once per day. Among regular sampling schemes, sampling approximately once every 18 hours would be ideal for these data sets, but small variations in time between samples could increase the range of values over which our estimates of $\eta$ are accurate, so there is some flexibility about the sampling scheme.

**Fig. 6:**
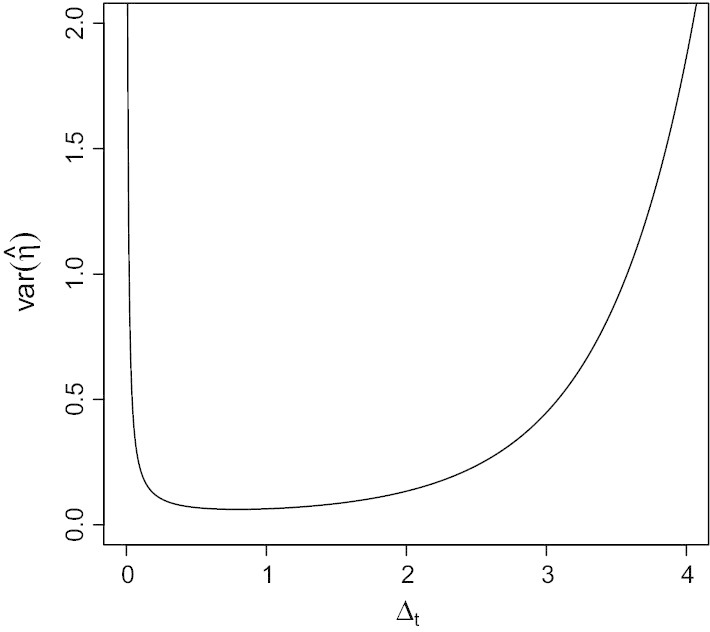
Relation between time difference $\Delta _t$ and ${{\,{\mathrm{Var}}\,}}({\hat{\eta }})$ for $\eta = 1$, $n=100$

From Proposition 2 in the "Methods" section, if the samples are evenly spaced with time difference $\Delta _t$, then ${{\,{\mathrm{Var}}\,}}\left( {\hat{\eta }}\right) =(I^{-1})_{\eta \eta }=\frac{e^{2\eta \Delta _t}-1}{n{\Delta _t}^2}$ does not depend on $\sigma$ or $\mu$ and is inversely proportional to *n*. Figure 6 shows the relation of ${{\,{\mathrm{Var}}\,}}\left( {\hat{\eta }}\right)$ with $\Delta _t$ for fixed $\eta =1$ and $n=100$.

Figure 6 shows that the accuracy of our estimated mean reversion rate is not harmed too much by sampling slightly more or less frequently, though as the sampling frequency deviates further from the optimal value, the effect of sampling frequency becomes more significant.

### Rates of mean reversion for different genera, people and body sites

A lot of previous work on temporal modelling of the microbiome, such as [15, 16] has been based on the assumption that the dynamics are similar for different individuals. If this assumption is valid, we expect our estimates of $\eta$ to be close for different individuals and perhaps body sites. Table 7a shows the estimates ${\hat{\eta }}$ for the genera which are abundant in at least five of the eight environments for the moving picture data, while Table 7b shows ${\hat{\eta }}$ for genera with more than 1,000,000 total reads for Subject A and more than 200,000 total reads for Subject B in the David et al. data.

**Table 7:** Comparison of estimated $\eta$ values (with standard errors) for an OU process across body sites and individuals for common genera in the moving picture data (a) and for the gut within individuals from both studies (b)

(a) Moving picture					
Genus	Person	Gut	Tongue	Left	Right
Actinomyces	1		2.51 (1.11)	1.20 (0.28)	
	2		1.42 (0.22)	1.06 (0.15)	1.91 (0.37)
Rothia	1		1.74 (0.50)	1.71 (0.49)	
	2		1.18 (0.16)	1.68 (0.29)	3.00 (1.13)
Porphyromonas	1		0.59 (0.13)	0.98 (0.22)	
	2	1.72 (0.32)	1.21 (0.17)	1.95 (0.38)	2.75 (0.88)
Prevotella	1		1.34 (0.33)	1.05 (0.24)	1.90 (0.60)
	2	1.42 (0.23)	1.45 (0.22)	2.23 (0.51)	1.75 (0.32)
Gemella	1		2.05 (0.69)	1.38 (0.35)	
	2		1.62 (0.27)	2.96 (1.07)	2.84 (0.96)
Granulicatella	1		1.36 (0.34)	1.27 (0.29)	
	2		1.74 (0.30)	2.53 (0.69)	2.27 (0.54)
Streptococcus	1		0.98 (0.22)	1.39 (0.35)	2.25 (0.86)
	2	1.24 (0.19)	2.29 (0.53)	2.41 (0.62)	2.28 (0.55)
Veillonella	1		1.28 (0.31)	1.16 (0.27)	
	2		1.81 (0.33)	1.63 (0.28)	1.83 (0.34)
Fusobacterium	1		0.66 (0.14)	0.63 (0.14)	0.61 (0.13)
	2		0.49 (0.07)	1.31 (0.19)	1.72 (0.31)
Neisseria	1		0.39 (0.09)	1.60 (0.44)	
	2		1.48 (0.23)	1.49 (0.24)	1.83 (0.34)
Pasteurellaceae (unclassified)	1		1.21 (0.29)	2.30 (0.90)	2.14 (0.77)
	2		1.28 (0.18)	2.16 (0.48)	3.67 (2.21)
Haemophilus	1		1.24 (0.30)	1.26 (0.30)	
	2		1.64 (0.27)	1.68 (0.29)	1.98 (0.40)

(b) Gut microbiome across both data sets					
	Person 1	Person 2	Subject A	Subject B	
Bacteroides	6.0000 (37.784)	2.5214 (0.743)	1.4410 (0.243)	0.6007 (0.113)	
Blautia	2.7268 (1.427)	1.4392 (0.240)	0.7966 (0.113)	0.8709 (0.166)	
Coprococcus	2.0080 (0.688)	1.0891 (0.161)	0.7619 (0.108)	0.6375 (0.119)	
Faecalibacterium	1.2732 (0.315)	2.4203 (0.671)	0.8978 (0.129)	0.7168 (0.134)	

**Fig. 7:**
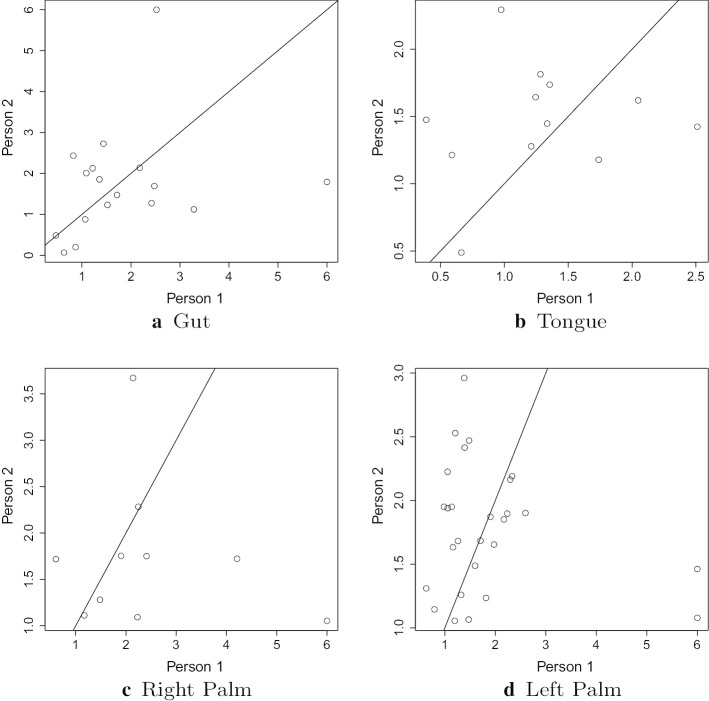
Comparison of estimated $\eta$ values data for common genera between two individuals for the same body site from the moving picture data. The lines $y=x$ are shown on each plot

**Fig. 8:**
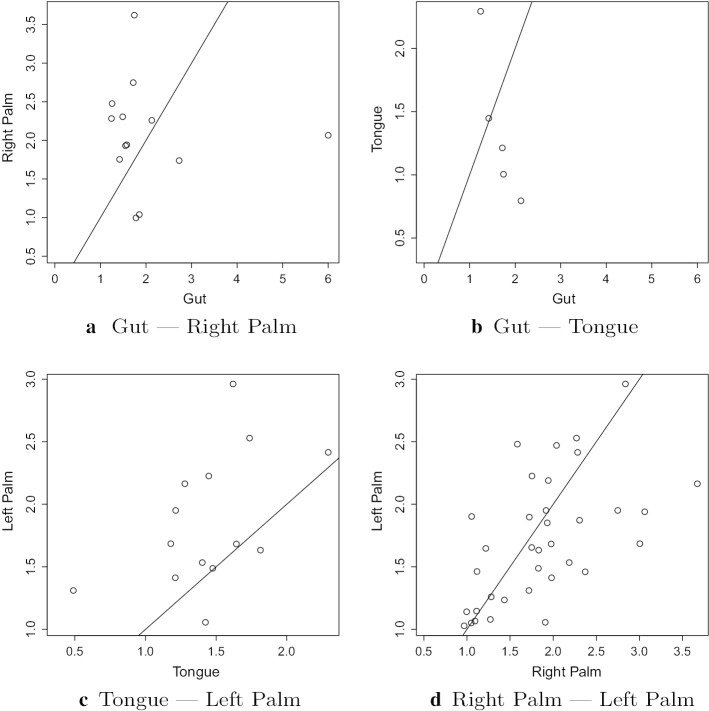
Comparison of estimated $\eta$ values for common genera between two body sites for Person 2 from the moving picture data. The lines $y=x$ are shown on each plot

There is substantial variation in the estimated mean reversion rates across different body sites, even for the same genus. This is also shown in Figs. 7 and 8, which include all genera abundant in both environments. Full estimates for $\eta$ for all abundant genera for both people in the moving picture data at all body sites are given in Additional file 1: Table 9 and Table 10 of Appendix D. Full results for the gut data from both studies are in Additional file 1: Table 15 of Appendix D. Comparing results between the two studies, the rates of mean reversion tend to be lower for the David et al. data than for the moving picture data. This is apparent both in Table 7(b) and in Fig. 4. The faster rates of mean reversion are not as universal as suggested by Table 7(b), though—there are several genera for which the rate of mean reversion is faster in the David et al. study. It is common for the differences between studies to be larger than within-study variation, so this difference is not extremely surprising, although one might expect the rate of mean reversion, to be more robust to the effects of different studies.

Overall, the rates of mean reversion for each genus differ between individuals. Figure 7 suggests a weak correlation between the rates for the two individuals. Similarly, for the David et al. data, the estimated ${\hat{\eta }}$ values for the two individuals are not correlated. This suggests that the results of methods that depend on the assumption that $\eta$ has the same value for all individuals, such as [15, 16], are likely to be incorrect.

From Figs. 7 and 8, we see that there is some correlation between the estimated mean reversion rates for a given genus in different environments, but it is fairly weak.

**Fig. 9:**
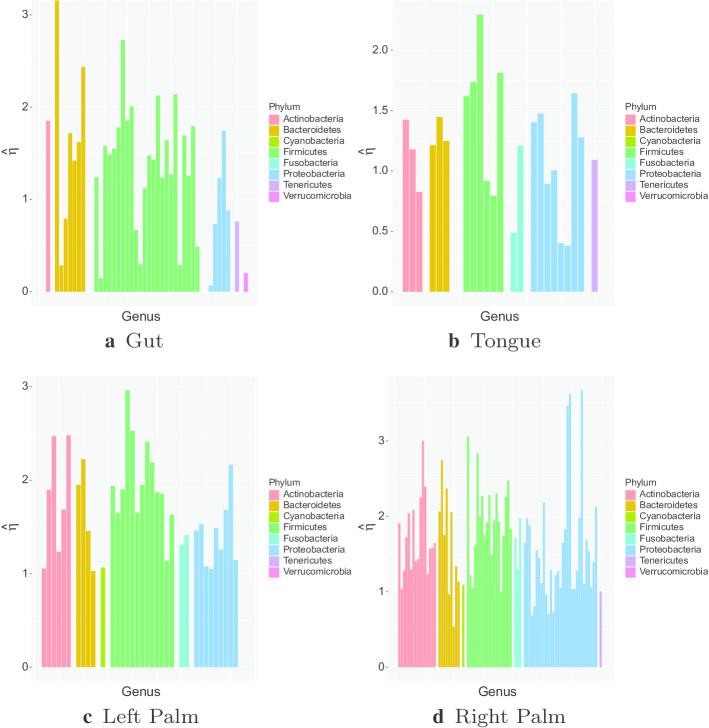
Comparison of estimated $\eta$ values for common genera at each body site for Person 2 from the moving picture data. The genera are arranged by taxonomy

**Fig. 10:**
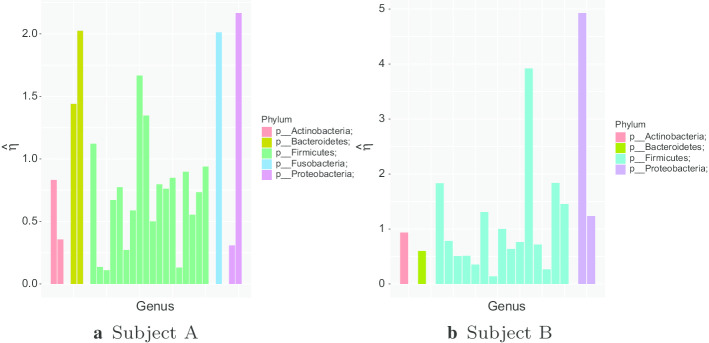
Comparison of estimated $\eta$ values for the David *et al.* dataset. The genera are arranged by taxonomy

Figure 9 shows the estimated mean reversion for the most abundant genera in each body site for Person 2 from the moving picture data. The genera are grouped by taxonomy, so genera in a given taxonomic grouping (phylum, class, order or family) are adjacent. From the plot, we see that estimated mean reversion rates are mostly between 1 and 2 in all environments, but there are more genera with slower mean reversion rates in the gut and tongue. There is a lot of variation between different genera in each environment. Figure 9 shows little similarity in the temporal dynamics of phylogenetically close genera. Most Firmicutes tend to have slightly faster mean reversion than other phyla, though a few show low mean reversion rates in the gut. Low mean reversion rates are rarer on the palms, which makes intuitive sense, since the palms are more exposed systems, so would be expected to be subject to external influences, which would reset any imbalance’s that might arise in the system. Figure 10 shows the same results for both subjects from the David et al. data. The estimated mean-reversion rates are slightly slower on average, and here Firmicutes seem to have slightly slower mean reversion for Subject A, but the number of abundant genera that were not Firmicutes is too small to draw reliable conclusions.

A similar figure showing the estimated mean reversion rates for various genera for Person 1 from the moving picture data is Additional file 1: Figure 3 of Appendix D. Again, there is no clear taxonomic pattern in the estimated mean reversion rates.

The estimated ${\hat{\sigma }}$ values for all abundant genera in the moving picture data are shown in Additional file 1: Tables 11 and 12 of Appendix D. There are several genera for which ${\hat{\sigma }}^2$ is extremely large. Upon inspection, these are conditionally rare genera, which are often absent from the samples, and occasionally occur in large blooms. It seems that ${\hat{\sigma }}^2$ is larger for the palms. This makes sense, since the palms are exposed to more external influences which can affect the microbial community. Interestingly ${\hat{\sigma }}^2$ is lower for the tongue than for the gut, indicating smaller random fluctuations. Given that the tongue is more exposed than the gut, this is slightly surprising. It can be partially explained because the most abundant genera in the tongue are more abundant, and more abundant genera are expected to be more stable. However, even if we compare genera with the same stable level in the gut and the tongue, the estimated value of ${\hat{\sigma }}$ is lower for the tongue.

## Discussion

In this paper, we have studied the temporal dynamics of the most abundant genera in the microbiomes at various body sites and for multiple individuals across two studies. We found evidence of temporal dependence and mean reversion in both data sets. For enclosed body sites, the abundant genera show stronger evidence of dependence than for external sites. Furthermore, all of the abundant genera show evidence of mean reversion. This provides statistical support for previous intuitive observations about the temporal stability of the microbiome.

Our model also estimates the time-scale of mean reversion for each genus. Under the OU model, the expected abundance decays exponentially towards the mean, and never reaches it. A common way to describe the time-scale in this context is the *half-life*, which is the time until the expected abundance is half way towards the mean. For the OU process, this time is $\lambda =\frac{\log (2)}{\eta }$. For the real data examples, our estimated values of $\eta$ were mostly between 0.4 and 2, which corresponds to a half-life of 0.35–1.7 days.

These results are consistent with the results of diet change studies, e.g. [9, 19]. Those studies observed that the microbiome can respond to changes in diet within one day. In the OU framework, a change in diet could be modelled as a change in equilibrium state. Under the OU model, the system would then adjust to the new equilibrium state with the same temporal dynamics. The time taken to revert half way towards the new system would be between 0.35 and 1.7 days, which is in line with the results from those studies.

The OU model also has some relation to the long-term variance of the system. The IBD studies [6, 7] found differences between IBD patients and healthy controls in the long-term variance of the system. Under the OU model, the long-term variance is $\frac{\sigma ^2}{2\eta }$. An increase in long-term variance between two populations could be explained in several ways:

- An increase in $\sigma ^2$. This would correspond to more rapid fluctuation in the microbial communities. It is unclear what biological processes could cause such a change.
- A decrease in $\eta$. This would correspond to a weakening of the mean-reversion mechanisms involved. For example, the host immune system might respond in an abnormal way to changes in the microbiome, reducing its stabilising effect.
- Temporal variation in the stable state. The OU model assumes that the stable state is fixed. However, there is strong evidence that this state is influenced by many external factors such as diet, lifestyle, antibiotics, etc. If these external factors vary more, or are more influential in IBD patients, then we would expect the asymptotic variance to increase.
- Variation in sampling bias. The sampling procedure is known to introduce large biases into microbiome data. It is conceivable that some of the many factors influencing this such as consistency of the stool, or blood in the stool, could lead to higher sampling variance in IBD patients than controls.
- An artefact of the methodology. The studies [6, 7] were based on beta-diversity measures, which could be sensitive to changes in the stable state. Since the stable state is different for IBD patients, comparisons of the variability of the microbiome for healthy controls and IBD patients depend heavily on the choice of measurement.

Further work is needed, with more frequent sampling, to determine which of these cases actually explains the observed results.

We can also examine how the half-life varies between different genera and environments. There is significant variation, but it is only weakly associated with the most obvious differences. The half-life is generally shorter for exposed environments such as the palms. This suggests that the external environment may act as a stabilising force, quickly driving the system back to the equilibrium state. The more enclosed states are not driven back so quickly, allowing the state to drift away from the equilibrium state for longer. On the other hand, the palms exhibit larger random fluctuations, so the long-term variance is actually larger for the palms than for the gut or tongue.

The rates of mean reversion for a fixed genus in multiple environments show weak correlation. This seems reasonable, since part of the mean reversion process is expected to depend on the characteristics of the particular genus, while the remainder is expected to be driven by the environment. We expect the strength of the correlation to vary according to the similarity between the environments. This seems to be the case, but the number of genera that are abundant in both environments is too small to make reliable conclusions about this.

Comparing the rates of mean reversion with the taxonomy of the genera, we do not see very strong relationships, even within a single environment. Mean reversion does appear to be slightly faster on average for Firmicutes than for Proteobacteria, but the variation within each phylum is much larger than the between-phylum variation. This suggests that more of the factors controlling microbial dynamics are related to particular genera, rather than higher level taxonomy. It would be interesting to study the dynamics at even higher resolution, but doing so would require adapting the methodology to better take account of the noise caused by sequencing.

We also derived the Fisher information matrix for the OU process and applied the theory of Fisher information to measure the accuracy of our estimated mean reversion velocity. For the moving picture data, we calculated the variance of our estimates, and showed that the estimates were reasonable. We also used Fisher information to determine the most efficient sampling schemes for future studies. If we insist on a minimum time difference between samples, in order to make our estimates more robust to model misspecification, then the optimal sampling scheme is to sample equally-spaced time points with difference $d_i\approx \frac{0.80}{\eta }$. Even in the case where the data perfectly follow an OU process, this sampling scheme is very efficient, causing a less than 1% increase in the variance of the estimator, compared to the theoretically best sampling scheme under the model. Given that we know the OU model is not perfect because of sequencing and other issues, we recommend this sampling scheme. We performed simulations to confirm that the asymptotic theory used here applies fairly accurately to our finite sample cases.

The optimal sampling scheme varies with the true rate of mean reversion. Thus, the optimal time difference is different for different body sites, or if we are interested in a particular subset of genera or OTUs. The optimal sampling frequency for enclosed body sites is slightly smaller than for exposed body sites (meaning we should have shorter intervals between samples for studying exposed body sites). The efficiency of the estimation remains reasonable if the sampling rate differs slightly from the optimal rate, so there is some flexibility in sampling. For the real data, sampling rates of approximately one sample per day should be adequate for studying the dynamics of these communities.

One limitation of the data set studied in this paper is that the sampling times are only available to the nearest day. We do not know what time of day the samples were taken. We have assumed that they were collected at the same time of day. However, if this assumption is not true, then the time of collection could affect the estimates under the OU model. It is unlikely that the difference will be large, but future studies would be able to estimate temporal dynamics more accurately if the time of sample collection and processing were available at higher resolution.

Another issue that might need to be considered for more frequent sampling is the possibility of diurnal cycles in the microbiome. There has been some evidence of diurnal cycles in mouse gut microbiomes [26]. These cycles could have a significant effect on the temporal dynamics if the sampling frequency is not a whole number of days.

### Future work

The OU process used in this paper is a very simple model with a linear velocity parameter mean reversion. It does not capture many of the aspects of the real data. In this section, we discuss some of the possible improvements to the model. The Fisher information theory from this paper can be extended to these improved models. We expect the estimated optimal sampling times not to be overly sensitive to the exact model specification, so that the same sampling scheme can be used to obtain good parameter estimates for multiple models.

One improvement to the model would be to allow multiple stable states. This could be achieved either by using a non-linear mean-reversion term or by a hidden Markov model where the equilibrium value varies following some process. While both of these models would describe a system with multiple stable states, they would have slightly different dynamics, and very different biological interpretations. Under the nonlinear equation, the microbiome drifts between stable states under its own dynamics. This change in state could then cause phenotypic changes in the host or environment. For example, dysbiosis might cause illness in the host. Under the hidden Markov model, the dynamics of the equilibrium state model external forces affecting the system. For example, an immune flare-up in response to some allergen might result in different dynamics of the microbiome. The distinction between these two models is of extreme clinical importance. Under the nonlinear model, monitoring the microbiome might provide early prediction of dysbiosis, and there is the potential for microbiome-based remedies. Under the hidden Markov model, changes to the microbiome occur after the external system change, so monitoring the microbiome offers less advance warning. Furthermore, if the microbiome changes are symptomatic rather than causal, microbiome-based interventions are unlikely to persist, or to remedy other symptoms. We hope to be able to distinguish between the two types of dynamics by comparing the fit of the respective two models.

It is widely believed that the temporal dynamics of the microbiome are driven by interactions between different OTUs, rather than each OTU acting independently. Therefore, it would be appropriate to develop a model which incorporates interactions between OTUs. There are already several differential equation models that have been used to model multiple systems. There is a natural multivariate version of the OU process, where the stable state is replaced by a vector, the mean reversion velocity $\eta$ is replaced by a matrix, and the random fluctuations are vector-valued. Alternatively, a number of multivariate differential equations used in ecology to model the growth of multiple populations, such as the Generalised Lotka–Volterra model or the Holling type-II model, could be equipped with a stochastic term to incorporate the random effects. Whatever model is used, parameter estimation would be challenging because of the high dimensionality. To deal with the high-dimensionality, some conditions to ensure sparsity of the estimated interaction parameters would be appropriate.

We should also incorporate measurement error in sampling to derive more accurate estimates. It is well-known that microbiome data are subject to significant errors and biases in DNA extraction and sequencing. These errors could have an impact on the estimated parameters of our model. By developing a model which incorporates as much as we know about the error in the sequencing procedure, we should be able to obtain more accurate parameter estimates. These errors are more significant at lower taxonomic levels, so by modelling the error structure, we will be able to apply our model to lower taxonomic levels, which could reveal more interesting biological patterns.

Another aspect we would like to include in the model in future is the non-homogeneity of the sample. Given that most microbes are several micrometres in diameter, it is very plausible that entirely separate microbial communities could coexist, separated by mere millimetres. In such a case, a faecal sample that has travelled the entire length of the gut, or even a sample collected from different areas on opposite sides of the tongue, would be expected to be a mixture of these different communities. However, the exact proportion of each community included in the sample will vary randomly between samples. This means that even if each of the microbial communities perfectly follows the stochastic differential equation, the overall sample will exhibit more complicated dynamics. Creating a model to include this effect will involve solving major statistical difficulties, but is a long-term goal for better modelling the temporal dynamics of microbial communities.

## Conclusions

There is clear evidence of temporal dependence among many of the abundant genera at all body sites. There is very strong evidence for mean reversion for all genera at all body sites. This provides support for previous observations about the temporal stability of the microbiome, but in a more statistically rigorous framework. Our model also estimates the time-scale of mean reversion. The estimated time for each genus to revert half way towards its mean varies between about 0.35 days for the most stable genera to about 1.7 days for the less stable genera. There is a large variation in all environments, but on average, mean reversion is slightly faster in exposed environments. The rate of mean reversion for a single genus varies between environments, but shows some weak correlation across different environments. The rate of mean reversion is not strongly associated with the taxonomy, though there are some general trends (e.g. mean reversion is on average slightly faster for Firmicutes than for Proteobacteria).

Using the Fisher information matrix, it is possible to estimate optimal sampling strategies for studying temporal dynamics of the microbiome. Based on this calculation, daily sampling is close to optimal for most genera. Estimates for fast-reverting genera would be improved by more frequent sampling. More accurate sample collection time data would also improve the accuracy of the estimated mean reversion.

## Methods

### Review of Ornstein–Uhlenbeck process

The Ornstein–Uhlenbeck process is a very simple stochastic differential equation in a single variable subject to mean reversion, meaning that, while fluctuating randomly, the variable’s values trend towards a stable mean value. This process has been used extensively to model situations where mean reversion is expected in a wide range of areas including physics [27], finance [28] and biology [29, 30]. It combines a linear mean-reversion term with a Brownian motion noise term. We therefore begin by reviewing Brownian motion.

#### Brownian motion

Brownian motion is a simple model of the behaviour of a system undergoing random fluctuations. It is the limiting process of a random walk as the step size and time between steps both converge to zero in a certain way. A thorough introduction to Brownian motion can be found in Karatzas and Shreve [31].

A stochastic process $W_t, t \geqslant 0$ with state space ${\mathbb {R}}$ is a *Standard Brownian Motion* (also called a *Wiener process*) if for any $0\leqslant s\leqslant t$, $W_t-W_s$ is normally distributed with mean 0 and variance $t-s$, and $W_t-W_s$ is independent of $\{W_r| 0\leqslant r\leqslant s\}$.

Let $W_t,t\ge 0$ be a standard Brownian motion. A stochastic process $\{X_t| t \geqslant 0\}$ given by

$$\begin{aligned} X_t= x_0+\mu _{\text {BM}} t+\sigma W_t, \quad t \ge 0 \end{aligned}$$

is called a *Brownian motion* with drift parameter $\mu _{\text {BM}} \in {{\mathbb {R}}}$, variance parameter $\sigma ^2 > 0$, and starting point $x_0 \in {{\mathbb {R}}}$.

If $X_t, t\geqslant 0$ follows Brownian motion with drift $\mu _{\text {BM}}$ and variance $\sigma ^2$ then for any $s,t\geqslant 0$, $X_{s+t}-X_s \sim N(\mu _{\text {BM}} t, \sigma ^2 t)$, and $X_{s+t}-X_s$ is independent of $\{X_r | 0\leqslant r\leqslant s\}$.

#### Ornstein–Uhlenbeck process

The OU process $X_t$ is defined by the following linear stochastic differential equation (SDE)

$$\begin{aligned} dX_t = \eta (\mu - X_t)dt +\sigma dW_t \end{aligned}$$

where $\eta >0$ is the velocity of the reversion process, $\mu$ is the stable state and $W_t$ is a Wiener process. We see that when $X_t>\mu$, the average derivative of $X_t$ is negative, meaning that on average $X_t$ will decrease; when $X_t<\mu$, the average derivative is positive. Thus in all cases $X_t$ will on average tend towards $\mu$, but the Brownian motion term adds random fluctuation to its trajectory. The rate at which $X_t$ trends towards the stable state grows larger as $X_t$ moves further away from the stable state. $\eta$ represents the average rate at which $X_t$ reverts to the stable state, while $\sigma$ represents the magnitude of the random fluctuations. A more complete introduction to the OU process can be found in any textbook on stochastic differential equations, for example [32].

There is a well-known explicit solution available to the OU process:

$$\begin{aligned} X_{s+t}|X_s\sim N\left( \mu +e^{-\eta t}(X_s-\mu ),\sigma ^2 \frac{1-e^{-2\eta t}}{2\eta }\right) \end{aligned}$$

### Testing temporal dependence and mean reversion of microbial dynamics

We use likelihood ratio tests to test for temporal dependence and for mean reversion. For testing temporal dependence, the hypotheses are:

$$H_0$$:
$X_t$ follow i.i.d. Normal distributions.
$$H_1$$:
$X_t$ follow an OU mean reverting process.

For testing for mean reversion, the hypotheses are:

$$H_0$$:
$X_t$ follow Brownian motion without drift ($\mu _{\text {BM}}=0$).
$$H_1$$:
$X_t$ follow an OU mean reverting process.

We perform separate tests for each person, body site and genus. Since we expect the majority of genera to reject the null hypotheses, we do not worry about multiple test correction, which reduces false positives in cases where the majority of tests cannot reject the null hypotheses. The i.i.d. normal model is a limiting case of the OU process model as $\eta \rightarrow \infty$, $\sigma \rightarrow \infty$ with $\frac{\sigma ^2}{\eta }$ fixed. The difference between the i.i.d. normal model and the OU process is just the serial correlation, so if the log-likelihood ratio test rejects the i.i.d. normal case, it suggests that there is serial dependence.

Brownian motion without drift is a special case of an OU process where the mean-reversion parameter is 0. Thus, testing against Brownian motion is a natural way to test for mean reversion.

The likelihood ratio statistics for these tests are not guaranteed to follow the usual $\chi ^2$ distribution. In the case of the i.i.d. normal hypothesis, this is because it is a limiting case, rather than an internal parameter value. In the Brownian motion case, the mean parameter $\mu$ of the OU process vanishes, so it is a non-identifiable special case. There is some theory on the asymptotic behaviour of these statistics in the case where the samples are equally spaced [33], but it does not apply in our case where the samples are not evenly spaced. Instead, we find the critical values for our hypothesis tests by simulation.

We simulate 5000 data sets using the same time points as the original data, under the null hypothesis. (We use a different simulation for each person and body site, as the time-points are different.) The likelihood ratio statistic is scale-invariant and translation-invariant for both tests (see Additional file 1: Appendix A.3 for a proof) so the null distribution is the same for any values for the parameters of the null distribution. Therefore, we only need to perform one simulation for each person and body-site. For the normal distribution we use $\sigma =1$ and $\mu =0$ for the simulation. For Brownian motion, we simulate with $x_0 = 0$, drift $\mu _{\text {BM}} = 0$ and variance $\sigma =1$.

For the Brownian motion simulation, the $\eta$ estimated for the OU mean-reverting process should be close to zero. To reduce the effect of rounding errors, we use a Taylor expansion approximation to evaluate the parameter estimates and the log-likelihood. Details of this approximation are in Additional file 1: Appendix A.2.

### Variance of estimated mean reversion rates and optimal sampling protocols

For estimating the variance of parameter estimates, we will use the statistical theory of Fisher Information. A detailed review of Fisher information can be found in [34] or [35]. For a model with parameter vector $\theta =(\theta _1,\ldots ,\theta _k)^T$, the Fisher information matrix at a vector $\theta =\theta _0$ is defined by $I=(I_{ij})_{i=1,\ldots ,k,j=1,\ldots ,k}$ where

$$I_{ij}=-{{\mathbb {E}}}_{X|\theta }\left( \left. \frac{\partial ^2 l(\theta ;X)}{\partial \theta _i\partial \theta _j}\right| _{\theta =\theta _0}\right)$$

where $l(\theta ;X)$ is the log-likelihood of data *X* at parameter value $\theta$.

The main use of Fisher information is the result that under common conditions, maximum likelihood estimates of parameters are asymptotically normal with variance given by the inverse of the Fisher information matrix. More explanation and a proof can be found in many textbooks on statistical theory, for example [36, 37].

#### Fisher information derivation for OU mean reverting process

To apply this to OTU temporal dynamics, we first calculate the Fisher information matrix for an OU process with parameters $\eta$, $\mu$ and $\sigma$.

##### Proposition 1

*For an OU process with parameter vector* $\theta =(\mu ,\eta ,\sigma )^T$, *sampled at time points* $t_0=0,t_1,\ldots ,t_n$, *the Fisher information matrix is given by*

$$\begin{aligned} I&= \left[ \begin{array}{ccc} \left[ I(\theta ) \right] _{\mu ,\mu } &\quad \left[ I(\theta ) \right] _{\mu ,\eta } &\quad \left[ I(\theta ) \right] _{\mu ,\sigma } \\ \left[ I(\theta ) \right] _{\mu ,\eta } &\quad \left[ I(\theta ) \right] _{\eta ,\eta } &\quad \left[ I(\theta ) \right] _{\eta , \sigma }\\ \left[ I(\theta ) \right] _{\mu ,\sigma } &\quad \left[ I(\theta ) \right] _{\eta , \sigma } &\quad \left[ I(\theta ) \right] _{\sigma ,\sigma } \end{array} \right] \\&=\left[ \begin{array}{ccc} \frac{2\eta }{\sigma ^2} {\sum }_{i = 1}^n\frac{e^{\frac{s_i}{2}}-1}{e^{\frac{s_i}{2}}+1}&{}\quad 0 &{}\quad 0 \\ 0 &{}\quad \frac{1}{4\eta ^2}{\sum }_{i=1}^n\left( {b_i}^2(e^{s_i}+1)-4b_i+2\right) &{} \quad \frac{1}{\sigma \eta }{\sum }_{i = 1}^n(b_i-1)\\ 0&{}\quad \frac{1}{\sigma \eta }{\sum }_{i = 1}^n(b_i-1) &{}\quad \frac{2n}{\sigma ^2} \end{array} \right] \end{aligned}$$

*where* $$s_i=2\eta (t_i-t_{i-1})$$ *and* $$b_i=\frac{s_i }{e^{s_i}-1}$$.

The proof of this proposition is in Additional file 1: Appendix B.1. In the case of equally spaced samples, we are able to simplify the Fisher information matrix. The following proposition is obtained by setting all $s_i=s=2\eta \Delta _t$, performing straightforward simplifications and inverting.

##### Proposition 2

*For an OU process with parameter vector* $\theta =(\mu ,\eta ,\sigma )^T$, *sampled at time points* $t_i=i\Delta _t$ *for some constant time spacing* $\Delta _t$ and $i=0,\ldots ,n$, *the inverse of the Fisher information matrix is given by*

$$\begin{aligned} I^{-1} =&\frac{1}{n}\left[ \begin{array}{ccc} \frac{\sigma ^2}{2\eta }\frac{e^{\frac{s}{2}}+1}{e^{\frac{s}{2}}-1} &{}\quad 0 &{}\quad 0 \\ 0 &{}\quad 4\eta ^2\frac{e^{s}-1}{s^2} &{}\quad 2\eta \sigma \left( \frac{e^{s}-1}{s^2}-\frac{1}{s}\right) \\ 0 &{}\quad 2\eta \sigma \left( \frac{e^{s}-1}{s^2}-\frac{1}{s}\right) &{}\quad \sigma ^2\left( \frac{e^{s}+1}{2(e^{s}-1)}-\frac{2}{s}+\frac{e^{s}-1}{s^2}\right) \end{array} \right] \end{aligned}$$

*where* $$s=2\eta \Delta _t$$.

#### Determining optimal sampling

The parameter that best describes the temporal dynamics is $\eta$, the rate of mean reversion. Therefore, if our objective is to understand the temporal dynamics of the microbiome, accurate estimation of $\eta$ is important. We will therefore focus on the sampling scheme that minimises ${{\,{\mathrm{Var}}\,}}({\hat{\eta }}) =\left[ I(\theta )^{-1} \right] _{\eta ,\eta }$. Theorem 3 gives the solution to this minimisation problem. The proof is in Additional file 1: Appendix B.2.

##### Theorem 3

1. *The optimal sampling scheme to minimise* ${{\,{\mathrm{Var}}\,}}({\hat{\eta }})$ *under an OU process is to sample the observations with time difference* $d_i=t_{i}-t_{i-1}$ *infinitesimal with probability* *p* *and equal to* $\frac{s^{\dagger }}{2\eta }$ *with probability* $1-p$, *where* $s^{\dagger }$ *and* *p* *are the solution to*
  $$\begin{aligned} 2\left( \frac{1}{s^{\dagger }}-\frac{1}{e^{s^{\dagger }}-1}\right) -1= 2s^{\dagger }\left( \frac{1}{s^{\dagger }}-\frac{1}{e^{s^{\dagger }}-1} \right) ^2\left( \frac{1}{s^{\dagger }}-\frac{1}{e^{s^{\dagger }}-1}-1\right) \end{aligned}$$
  *and*
  $$\begin{aligned} p=\frac{1}{2}-\frac{{s^{\dagger }}^2(e^{s^{\dagger }}-1)}{4(e^{s^{\dagger }}-1-s^{\dagger })^2} \end{aligned}$$
  *Numerically these values can be solved as* $s^{\dagger }=1.956493$ *and* $p=0.1572033$. *For this optimal sampling scheme*, ${{\,{\mathrm{Var}}\,}}({\hat{\eta }})=\frac{6.12679\eta ^2}{n}$.
2. *Let* $s_l$ *be the solution to*
  $$\begin{aligned} s_l^2(e^{s_l}-1)-c_0(e^{s_l}-1)^2=2(b_0(e^{s_l}-1)-s_l)^2 \end{aligned}$$
  *with* $c_0=\sup \frac{x^2}{e^x-1}=0.6476102$ *and* $b_0=1-\sqrt{1-c_0}=0.4063757$. *Numerically, this is* $s_l=0.5844618$. *If samples from an OU process must be collected with time difference* $d_i\geqslant \frac{s_l}{2\eta }$, *then the optimal sampling scheme is to sample evenly-spaced observations with* $d_i=\frac{s_0}{2\eta }$, *where* $s_0$ *is the solution to*
  $$\begin{aligned} (2-s_0)(e^{s_0}-1)=s_0 \end{aligned}$$
  *Numerically*, $s_0=1.59362426$, *and for this sampling scheme* ${{\,{\mathrm{Var}}\,}}\left( {\hat{\eta }}\right) =\frac{6.176555\eta ^2}{n}$.
3. *If* $\sigma$ *is known for an OU process, then the optimal sampling scheme to minimise* ${{\,{\mathrm{Var}}\,}}({\hat{\eta }})$ *is to sample evenly-spaced observations with time difference* $d_i=\frac{s_k}{2\eta }$ *where* $s_k$ *is the solution to*
  $$\begin{aligned} 4(e^{s_k}-1)^2+s_k^2e^{s_k}(3+e^{s_k})=6s_ke^{s_k}(e^{s_k}-1)+2s_k(e^{s_k}-1) \end{aligned}$$
  *Numerically, we get* $s_k=5.109858$. *In this case* ${{\,{\mathrm{Var}}\,}}\left( {\hat{\eta }}\right) =\frac{1.964279\eta ^2}{n}$.

The optimality of sampling with infinitesimal time differences is slightly counter-intuitive. Under an OU model, as time difference tends to zero, the Brownian motion term dominates, and therefore, samples with infinitesimal time differences are most efficient for estimating $\sigma$. Therefore, the optimal sampling scheme from Theorem 3(1) uses some samples which are very informative about $\sigma$, and others which give information about both $\sigma$ and $\eta$. We can see from part (3) of the theorem that the remaining samples are still much more frequent than would be needed if we already knew $\sigma$. This makes sense, because in the OU process, the long-term variance is given by $\frac{\sigma ^2}{2\eta }$, so if $\sigma$ is known then we can estimate $\eta$, even using samples at very large time difference.

When using samples with small time differences, $\sigma$ is estimated from the ratios $\frac{(X_{t_i}-X_{t_{i-1}})^2}{t_i-t_{i-1}}$. Because this involves differences between close quantities, it is very sensitive to any model misspecification, such as measurement error in *t* or *X*. Therefore, in practice, it may be sensible to limit the frequency of sampling as in part (2) of Theorem 3. With less frequent sampling, the OU model is likely to be a better fit for the data, so the sampling scheme from part (2) is likely to be more useful in practice. Even in the perfect case with no model misspecification, the loss from using equally spaced samples as in part (2) over the scheme given by part (1) is relatively small.

## Simulation

The asymptotic normality of MLE theorem states that for a large enough sample size, the asymptotic behaviour of MLEs can be described by the Fisher information matrix. However, it does not specify what sample size is needed for this asymptotic approximation to be reasonable. We therefore conducted a simulation study to confirm that the asymptotic approximation can be used for realistic sample sizes.

### Simulation design

In order to test the estimated covariance matrix, we simulated data sets under an OU model, with $\mu =0$, $\eta \in \{0.5,1,2\}$ and $\sigma \in \{1,2,4\}$, with time step $\Delta _t=1$, with different sample sizes *n*. These values cover a range of scenarios similar to the values estimated from the real data. For each sample, we computed the MLEs ${\hat{\eta }}$, ${\hat{\sigma }}$ and ${\hat{\mu }}$. We compared the covariance matrices estimated from 100,000 simulations with the asymptotic covariance matrices predicted using the Fisher information matrix, using the following matrix dissimilarity measure

$$\begin{aligned} d^2(A, B ) = \frac{\Vert A-B\Vert ^2}{\Vert A\Vert ^2} \end{aligned}$$

where $\Vert A\Vert$ is the Euclidean norm of *A*, i.e. $\Vert A\Vert ^2=\sum _{i,j}{A_{ij}}^2$. We measured the dissimilarity $d^2(I^{-1},{\hat{V}})$, where *I* is the Fisher information matrix, and ${\hat{V}}$ is the empirically estimated covariance matrix.

### Simulation results

The simulation results are shown in Table 8.

**Table 8:** Distance between inverse Fisher information matrix and sample covariance matrix for various sample sizes

$\eta$	$\sigma$	*n*					
		5000	1000	500	100	50	10
0.5	1	0.054	0.053	0.052	0.058	0.125	0.996
	2	0.066	0.065	0.064	0.055	0.052	0.998
	4	0.066	0.065	0.064	0.055	0.040	0.995
1	1	0.008	0.008	0.011	0.139	0.330	0.968
	2	0.057	0.051	0.046	0.106	0.266	1.000
	4	0.122	0.109	0.099	0.056	0.121	0.853
2	1	0.001	0.033	0.147	0.078	0.021	12.281
	2	0.002	0.028	0.123	0.073	0.047	12.168
	4	0.005	0.020	0.102	0.045	0.098	7.586

For small sample sizes (mostly $n\leqslant 100$) there were a few simulations where ${\hat{\eta }}=\infty$, i.e. there is no estimated correlation between consecutive samples. These simulations were removed from the variance calculation.

As expected, the observed covariance matrix gets closer to the Fisher information matrix as sample size *n* increases. (Note that the errors in the table are relative errors). For $\eta =0.5$ and $\eta =1$, the approximation becomes reasonably accurate, (with an error of about 10% in the covariance matrix), somewhere between sample size 100 and 500. Thus, for the moving picture data set [18] and the David et al. data set [19], we expect the inverse of the Fisher information matrix to provide a fairly accurate estimate of the variance of the parameter estimates.

## Supplementary information

**Additional file 1:** Supplemental Appendices.

## Data Availability

The data analysed in this paper are from Caporaso et al. [18] and from David et. al. [19], and are studies 550 and 2202, respectively, in *Qiita*. They can be accessed via the following links: https://qiita.ucsd.edu/study/description/550 and https://qiita.ucsd.edu/study/description/2202.

## Abbreviations

OU: Ornstein–Uhlenbeck
IBD: Irritable bowel disease
i.i.d.: Independent identically distributed
MLE: Maximum likelihood estimator
PCR: Polymerase chain reaction
OTU: Operational taxonomic unit
