# Supplementary material for: Application of OU processes to modelling temporal dynamics of the human microbiome, and calculating optimal sampling schemes
Source: BMC Bioinformatics. 2020 Oct 12;21:450. doi: 10.1186/s12859-020-03747-4 (PMC7549249; doi:10.1186/s12859-020-03747-4)
Supplement: Supplementary file 1 — Additional file 1: Supplemental Appendices. [file 12859_2020_3747_MOESM1_ESM.pdf]

Appendices to "Application of OU processes to modelling temporal dynamics of the human microbiome, and calculating optimal sampling schemes."

Toby Kenney, Junqiu Gao, Hong Gu
Department of Mathematics and Statistics, Dalhousie University

August 29, 2020

A Log likelihood calculations

A.1 Exact log likelihood

For Brownian motion, the log likelihood for a set of observed $X(t)$ values, $x_0, x_1, \dots x_n$ corresponding to times $t_0, \cdots t_n$, is

$$l(x;\mu,\sigma) = -\frac{1}{2}\sum_{i=1}^{n}\log(2\pi\sigma^2(t_i - t_{i-1})) - \sum_{i=1}^{n}\frac{[x_i - (x_{i-1} + \mu(t_i - t_{i-1}))]^2}{2\sigma^2(t_i - t_{i-1})}$$

Setting the first derivatives of this equal to 0, we get the following maximum likelihood estimates:

$$\hat{\mu} = \frac{x_n - x_1}{t_n - t_1}$$

$$\hat{\sigma}^2 = \frac{1}{n}\sum_{i=1}^{n}\frac{[x_i - (x_{i-1} + \hat{\mu}(t_i - t_{i-1}))]^2}{t_i - t_{i-1}}$$

For the OU process, the conditional distributions are also normal, so it is

easy to compute the log-likelihood

$$
\begin{aligned}
l(\boldsymbol{x};\mu,\eta,\sigma) = &-\frac{n}{2}\log(2\pi) - \frac{n}{2}\log\left(\frac{\sigma^2}{2\eta}\right) - \frac{1}{2}\sum_{i=1}^{n}\log(1-e^{-2\eta(t_i-t_{i-1})}) \\
&- \frac{\eta}{\sigma^2}\sum_{i=1}^{n}\frac{(x_i-\mu-(x_{i-1}-\mu)e^{-\eta(t_i-t_{i-1})})^2}{1-e^{-2\eta(t_i-t_{i-1})}}
\end{aligned} \tag{1}
$$

It is straightforward to solve $\frac{\partial l(\boldsymbol{x};\mu,\eta,\sigma)}{\partial\mu} = 0$ and $\frac{\partial l(\boldsymbol{x};\mu,\eta,\sigma)}{\partial\sigma} = 0$. These equations give the following solutions for fixed η (see [**?**])

$$
\hat{\mu} = f(\hat{\eta}) = \sum_{i=1}^{n}\frac{x_i - x_{i-1}e^{-\hat{\eta}(t_i-t_{i-1})}}{1+e^{-\hat{\eta}(t_i-t_{i-1})}}\left(\sum_{i=1}^{n}\frac{1-e^{-\hat{\eta}(t_i-t_{i-1})}}{1+e^{-\hat{\eta}(t_i-t_{i-1})}}\right)^{-1} \tag{2}
$$

$$
\hat{\sigma}^2 = g(\hat{\mu},\hat{\eta}) = \frac{2\hat{\eta}}{n}\sum_{i=1}^{n}\frac{\left(x_i-\hat{\mu}-(x_{i-1}-\hat{\mu})e^{-\hat{\eta}(t_i-t_{i-1})}\right)^2}{1-e^{-2\hat{\eta}(t_i-t_{i-1})}} \tag{3}
$$

Plugging in the estimates $\hat{\mu}$ and $\hat{\sigma}^2$, the profile log likelihood function becomes:

$$
\begin{aligned}
V(\eta) = &-\frac{n}{2}\log(2\pi) - \frac{n}{2}\log\left(\frac{g(f(\eta),\eta)}{2\eta}\right) - \frac{1}{2}\sum_{i=1}^{n}\log\left(1-e^{-2\eta(t_i-t_{i-1})}\right) \\
&- \frac{\eta}{g(f(\eta),\eta)}\sum_{i=1}^{n}\frac{\left(x_i-f(\eta)-(x_{i-1}-f(\eta))e^{-\eta(t_i-t_{i-1})}\right)^2}{1-e^{-2\eta(t_i-t_{i-1})}}
\end{aligned} \tag{4}
$$

It is straightforward to find the optimal $\hat{\eta}$ from this equation, and use Equations (2) and (3) to get MLE estimates $\hat{\mu}$ and $\hat{\sigma}^2$. To ensure robustness in our work, we used an exhaustive grid search with step size 0.0001 in our computations.

A.2 Taylor Expansion of OU Process Likelihood

When η is close to zero, the formulae (2)–(4) are numerically unstable. We therefore replace the unstable parts of them using Taylor series expansions.

Theorem 1.

$$V(\eta) = -\frac{n}{2}\log(2\pi\hat{\sigma}_T^2) - \frac{1}{2}\sum_{i=1}^{n}\left[\log(d_i) + P_i - \frac{P_i^2}{2} + \frac{P_i^3}{3} - \frac{P_i^4}{4} - \frac{(x_i - x_{i-1})^2}{2\hat{\sigma}_T^2 M_i}\right]$$
$$- \frac{\eta}{\hat{\sigma}_T^2}\sum_{i=1}^{n}(x_{i-1} - \hat{\mu}_T)\left(\frac{2x_i - \hat{\mu}_T(1 - e^{-\eta d_i})}{1 + e^{-\eta d_i}} - x_{i-1}\right) + o(\eta^4) \quad (5)$$

where

$$\hat{\mu}_T = \sum_{i=1}^{n}\frac{x_i - x_{i-1}e^{-\hat{\eta} d_i}}{1 + e^{-\hat{\eta} d_i}}\left(\sum_{i=1}^{n}\frac{N_i}{1 + e^{-\hat{\eta} d_i}}\right)^{-1} \quad (6)$$
$$\hat{\sigma}_T^2 = \frac{1}{n}\sum_{i=1}^{n} M_i^{-1}(x_i - \hat{\mu}_T - (x_{i-1} - \hat{\mu}_T)e^{-\hat{\eta} d_i})^2 \quad (7)$$

and

$$d_i = t_i - t_{i-1} \qquad M_i = d_i - \frac{2\eta d_i^2}{2!} + \frac{4\eta^2 d_i^3}{3!} - \frac{8\eta^3 d_i^4}{4!} + \frac{16\eta^4 d_i^5}{5!}$$
$$N_i = \eta d_i - \frac{\eta^2 d_i^2}{2!} + \frac{\eta^3 d_i^3}{3!} - \frac{\eta^4 d_i^4}{4!} \qquad P_i = -\frac{2\eta d_i}{2!} + \frac{4\eta^2 d_i^2}{3!} - \frac{8\eta^3 d_i^3}{4!} + \frac{16\eta^4 d_i^4}{5!}$$

Proof. From the Taylor expansion, we have

$$1 - e^{-\eta d_i} = N_i + o(\eta^4) \qquad \frac{1 - e^{-2\eta d_i}}{2\eta} = M_i + o(\eta^4)$$
$$\frac{1 - e^{-2\eta d_i}}{2\eta d_i} = 1 + P_i + o(\eta^4) \qquad \log\left(\frac{1 - e^{-2\eta d_i}}{2\eta d_i}\right) = P_i - \frac{P_i^2}{2} + \frac{P_i^3}{3} - \frac{P_i^4}{4} + o(\eta^4)$$

Substituting these approximations into the formulae for $\hat{\mu}$ and $\hat{\sigma}^2$ gives $\hat{\mu} = \hat{\mu}_\mathrm{T} + o(\eta^4)$ and $\hat{\sigma}^2 = \hat{\sigma}_T^2 + o(\eta^4)$. Plugging these into Equation (4) gives

$$V(\eta)_\mathrm{T} = -\frac{n}{2}\log(2\pi) - \frac{1}{2}\sum_{i=1}^{n}\log\left(\frac{(\hat{\sigma}_\mathrm{T}^2 + o(\eta^4))(1 - e^{-2\eta d_i})}{2\eta}\right)$$
$$- \frac{\eta}{\hat{\sigma}_T^2 + o(\eta^4)}\sum_{i=1}^{n}\frac{(x_i - \hat{\mu}_\mathrm{T} - (x_{i-1} - \hat{\mu}_\mathrm{T})e^{-\eta d_i} + o(\eta^4))^2}{1 - e^{-2\eta d_i}} \quad (8)$$

expanding the third term:

$$\frac{(x_i - \hat{\mu}_{\mathrm{T}} - (x_{i-1} - \hat{\mu}_{\mathrm{T}})e^{-\eta d_i})^2}{1 - e^{-2\eta d_i}} = \left(\frac{(x_i - x_{i-1})^2}{2\eta M_i + o(\eta^4)}\right) + \left(\frac{2(x_i - x_{i-1})(x_{i-1} - \hat{\mu}_{\mathrm{T}})}{1 + e^{-\eta d_i}}\right) + \left(\frac{(x_{i-1} - \hat{\mu}_{\mathrm{T}})^2(1 - e^{-\eta d_i})}{1 + e^{-\eta d_i}}\right) + o(\eta^4)$$

and substituting the Taylor approximations into the expression completes the proof.

□

A.3 Invariance of Likelihood Ratio Statistics

We used log-likelihood ratio tests to assess whether the OU process fits the data better than an i.i.d. normal model or a Brownian motion model. To find the null distributions, we use a simulation with chosen parameters. We want to show that the null distribution does not depend on the parameters.

We start by fixing our notation. Let $l_{\mathrm{OU}}(\boldsymbol{x}; \mu, \eta, \sigma)$ denote the log-likelihood under an OU process with parameters μ, η and σ for the data $\boldsymbol{x}$; and let $l_{\mathrm{N}}(\boldsymbol{x}; \mu, \sigma)$ denote the log-likelihood of an i.i.d. normal distribution with mean μ and variance σ^2 for the data x. Let $\hat{\mu}_{\mathrm{OU},\boldsymbol{x}}$, $\hat{\eta}_{\mathrm{OU},\boldsymbol{x}}$, and $\hat{\sigma}_{\mathrm{OU},\boldsymbol{x}}$ be the MLEs under an OU model for the data $\boldsymbol{x}$; let $\hat{\sigma}_{\mathrm{BM},\boldsymbol{x}}$ be the MLE under Brownian motion for the data $\boldsymbol{x}$; and let $\hat{\mu}_{\mathrm{N},\boldsymbol{x}}$ and $\hat{\sigma}_{\mathrm{N},\boldsymbol{x}}$ be the MLEs for an i.i.d. normal model for the data x.

The reason the distribution does not depend on μ and σ is that both distributions have scale and location parameters, so the MLE estimates are linearly transformed. This is formalised in the following lemmas.

Lemma 2. *If $\boldsymbol{y} = a\boldsymbol{x} + b$ for scalars a and b, then*

$$\begin{aligned}\hat{\mu}_{OU,\boldsymbol{y}} &= a\hat{\mu}_{OU,\boldsymbol{x}} + b\\ \hat{\eta}_{OU,\boldsymbol{y}} &= \hat{\eta}_{OU,\boldsymbol{x}}\\ \hat{\sigma}_{OU,\boldsymbol{y}} &= a\hat{\sigma}_{OU,\boldsymbol{x}}\end{aligned}$$

and

$$l_{OU}(\boldsymbol{y}; \hat{\mu}_{OU,\boldsymbol{y}}, \hat{\eta}_{OU,\boldsymbol{y}}, \hat{\sigma}_{OU,\boldsymbol{y}}) = l_{OU}(\boldsymbol{x}; \hat{\mu}_{OU,\boldsymbol{x}}, \hat{\eta}_{OU,\boldsymbol{x}}, \sigma_{OU,\boldsymbol{x}}) - n\log(a)$$

Proof. Let

$$h(n,\eta,\sigma) = -\frac{n}{2}\log(2\pi) - \frac{n}{2}\log\left(\frac{\sigma^2}{2\eta}\right) - \frac{1}{2}\sum_{i=1}^{n}\log(1-e^{-2\eta(t_i-t_{i-1})})$$

We have that

$$\begin{aligned}
l_{\mathrm{OU}}(\boldsymbol{y};\mu,\eta,\sigma) &= h(n,\eta,\sigma) - \frac{\eta}{\sigma^2}\sum_{i=1}^{n}\frac{(y_i-\mu-(y_{i-1}-\mu)e^{-\eta(t_i-t_{i-1})})^2}{1-e^{-2\eta(t_i-t_{i-1})}} \\
&= h(n,\eta,\sigma) - \frac{\eta}{\sigma^2}\sum_{i=1}^{n}\frac{(ax_i+b-\mu-(ax_{i-1}+b-\mu)e^{-\eta(t_i-t_{i-1})})^2}{1-e^{-2\eta(t_i-t_{i-1})}} \\
&= h(n,\eta,\sigma) - \frac{a^2\eta}{\sigma^2}\sum_{i=1}^{n}\frac{\left(x_i-\frac{\mu-b}{a}-\left(x_{i-1}-\frac{\mu-b}{a}\right)e^{-\eta(t_i-t_{i-1})}\right)^2}{1-e^{-2\eta(t_i-t_{i-1})}} \\
&= l_{\mathrm{OU}}\left(\boldsymbol{x};\frac{\mu-b}{a},\eta,\frac{\sigma}{a}\right) - n\log(a)
\end{aligned}$$

Thus, in particular, for any μ, η and σ we have

$$\begin{aligned}
l_{\mathrm{OU}}(\boldsymbol{y};\mu,\eta,\sigma) &= l_{\mathrm{OU}}\left(\boldsymbol{x};\frac{\mu-b}{a},\eta,\frac{\sigma}{a}\right) - n\log(a) \\
&\leqslant l_{\mathrm{OU}}(x;\hat{\mu}_{\mathrm{OU},\boldsymbol{x}},\eta,\hat{\sigma}_{\mathrm{OU},\boldsymbol{x}}) - n\log(a) \\
&= l_{\mathrm{OU}}(y;a\hat{\mu}_{\mathrm{OU},\boldsymbol{x}}+b,\eta,a\hat{\sigma}_{\mathrm{OU},\boldsymbol{x}})
\end{aligned}$$

which completes the proof. □

Lemma 3. *If* $\boldsymbol{y} = a\boldsymbol{x} + b$ *then*

$$\begin{aligned}
\hat{\sigma}_{BM,\boldsymbol{y}} &= a\hat{\sigma}_{BM,\boldsymbol{x}} \\
l_{BM}(\boldsymbol{y};\hat{\sigma}_{BM,\boldsymbol{y}}) &= l_{BM}(\boldsymbol{x};\hat{\sigma}_{BM,\boldsymbol{x}}) - n\log(a)
\end{aligned}$$

Proof. Let

$$h(n,\sigma) = -\frac{1}{2}\sum_{i=1}^{n}\log(2\pi\sigma^2(t_i-t_{i-1}))$$

We have that

$$\begin{aligned}
l_{\mathrm{BM}}(\boldsymbol{y};\sigma) &= h(n,\sigma) - \sum_{i=1}^{n} \frac{(y_i - y_{i-1})^2}{2\sigma^2(t_i - t_{i-1})} \\
&= h(n,\sigma) - \sum_{i=1}^{n} \frac{(ax_i - ax_{i-1})^2}{2\sigma^2(t_i - t_{i-1})} \\
&= l_{\mathrm{BM}}\left(\boldsymbol{x};\frac{\sigma}{a}\right) - n\log(a)
\end{aligned}$$

Thus, in particular

$$l_{\mathrm{BM}}(\boldsymbol{y};\sigma) = l_{\mathrm{BM}}\left(\boldsymbol{x};\frac{\sigma}{a}\right) - n\log(a) \leqslant l_{\mathrm{BM}}(x;\hat{\sigma}_{\mathrm{BM},\boldsymbol{x}}) - n\log(a) = l_{\mathrm{BM}}(y;a\hat{\sigma}_{\mathrm{BM},\boldsymbol{x}})$$

which completes the proof. □

Theorem 4. *The null distribution of the likelihood ratio statistic for comparing an OU process to an i.i.d. normal model, and the likelihood ratio statistic for comparing an OU process to Brownian motion are independent of the true parameters of the normal distribution or Brownian motion.*

Proof. If the null distribution of a random vector X is i.i.d. normal with mean μ and variance σ^2, then $Y = \frac{X-\mu}{\sigma}$ is i.i.d. standard normal. For any random draws $\boldsymbol{x}$ from X, $\boldsymbol{y} = \frac{\boldsymbol{x}-\mu}{\sigma}$ is a random draw from Y, and by Lemma 2:

$$\begin{aligned}
l_{\mathrm{OU}}(\boldsymbol{x};\hat{\mu}_{\mathrm{OU},\boldsymbol{x}},\hat{\eta}_{\mathrm{OU},\boldsymbol{x}},\hat{\sigma}_{\mathrm{OU},\boldsymbol{x}}) - l_{\mathrm{OU}}(\boldsymbol{y};\hat{\mu}_{\mathrm{OU},\boldsymbol{y}},\hat{\eta}_{\mathrm{OU},\boldsymbol{y}},\sigma_{\mathrm{OU},\boldsymbol{y}}) &= -n\log(\sigma) \\
&= l_{\mathrm{N}}(\boldsymbol{x};\hat{\mu}_{\mathrm{N},\boldsymbol{x}},\hat{\sigma}_{\mathrm{N},\boldsymbol{x}}) - l_{\mathrm{N}}(\boldsymbol{y};\hat{\mu}_{\mathrm{N},\boldsymbol{y}},\hat{\sigma}_{\mathrm{N},\boldsymbol{y}})
\end{aligned}$$

so

$$l_{\mathrm{OU}}(\boldsymbol{x};\hat{\mu}_{\mathrm{OU},\boldsymbol{x}},\hat{\eta}_{\mathrm{OU},\boldsymbol{x}},\sigma_{\mathrm{OU},\boldsymbol{x}}) - l_{\mathrm{N}}(\boldsymbol{x};\hat{\mu}_{\mathrm{N},\boldsymbol{x}},\hat{\sigma}_{\mathrm{N},\boldsymbol{x}}) = l_{\mathrm{OU}}(\boldsymbol{y};\hat{\mu}_{\mathrm{OU},\boldsymbol{y}},\hat{\eta}_{\mathrm{OU},\boldsymbol{y}},\hat{\sigma}_{\mathrm{OU},\boldsymbol{y}}) - l_{\mathrm{N}}(\boldsymbol{y};\hat{\mu}_{\mathrm{N},\boldsymbol{y}},\hat{\sigma}_{\mathrm{N},\boldsymbol{y}})$$

Similarly, if the null distribution of a random vector X follows Brownian motion with variance σ^2, then $Y = \frac{X-X_0}{\sigma}$ follows standard Brownian motion. For any random draws $\boldsymbol{x}$ from X, $\boldsymbol{y} = \frac{\boldsymbol{x}-x_0}{\sigma}$ is a random draw from Y, and by Lemma 2 and Lemma 3:

$$\begin{aligned}
l_{\mathrm{OU}}(\boldsymbol{y};\hat{\mu}_{\mathrm{OU},\boldsymbol{y}},\hat{\eta}_{\mathrm{OU},\boldsymbol{y}},\hat{\sigma}_{\mathrm{OU},\boldsymbol{y}}) - l_{\mathrm{OU}}(\boldsymbol{x};\hat{\mu}_{\mathrm{OU},\boldsymbol{x}},\hat{\eta}_{\mathrm{OU},\boldsymbol{x}},\hat{\sigma}_{\mathrm{OU},\boldsymbol{x}}) &= -n\log\left(\frac{1}{\sigma}\right) \\
&= l_{\mathrm{BM}}(\boldsymbol{y};\hat{\sigma}_{\mathrm{BM},\boldsymbol{x}}) - l_{\mathrm{BM}}(\boldsymbol{x};\hat{\sigma}_{\mathrm{BM},\boldsymbol{y}})
\end{aligned}$$

so

$$l_{\text{OU}}(\boldsymbol{x};\hat{\mu}_{\text{OU},\boldsymbol{x}},\hat{\eta}_{\text{OU},\boldsymbol{x}},\sigma_{\text{OU},\boldsymbol{x}})-l_{\text{BM}}(\boldsymbol{x};\hat{\sigma}_{\text{BM},\boldsymbol{x}}) = l_{\text{OU}}(\boldsymbol{y};\hat{\mu}_{\text{OU},\boldsymbol{y}},\hat{\eta}_{\text{OU},\boldsymbol{y}},\hat{\sigma}_{\text{OU},\boldsymbol{y}})-l_{\text{BM}}(\boldsymbol{y};\hat{\sigma}_{\text{BM},\boldsymbol{y}})$$

□

B Fisher Information Calculation

B.1 Fisher Information

The observed information matrix is the negative of the Hessian matrix of the log-likelihood functions. To simplify the algebra we define $V_i = (x_i - \mu) - (x_{i-1} - \mu)e^{-\eta d_i}$ and $W_i = (x_{i-1} - \mu) - (x_i - \mu)e^{-\eta d_i}$. This means that the log-likelihood (with constant term removed) is

$$l_{\text{OU}}(\boldsymbol{x};\mu,\eta,\sigma) = -\frac{1}{2}\sum_{i=1}^{n}\left(\log\left(\frac{\sigma^2}{2\eta}\right) + \log(1-e^{-2\eta d_i}) + \frac{2\eta}{\sigma^2}\frac{{V_i}^2}{1-e^{-2\eta d_i}}\right) \quad (9)$$

Lemma 5. *For $V_i = (x_i - \mu) - (x_{i-1} - \mu)e^{-\eta d_i}$ and $W_i = (x_{i-1} - \mu) - (x_i - \mu)e^{-\eta d_i}$,*

$$\begin{aligned}
\frac{\partial}{\partial \eta}\left[\frac{V_i^2}{1-e^{-2\eta d_i}}\right] &= \frac{d_i e^{-\eta d_i}}{(1-e^{-2\eta d_i})^2}\left[2V_iW_i\right] \\
\frac{\partial^2}{\partial \eta^2}\left[\frac{V_i^2}{1-e^{-2\eta d_i}}\right] &= \frac{2d_i^2 e^{-\eta d_i}}{(1-e^{-2\eta d_i})^3}\left[e^{-\eta d_i}({V_i}^2+{W_i}^2) - V_iW_i(1+e^{-2\eta d_i})\right] \\
\frac{\partial V_i}{\partial \mu} &= e^{-\eta d_i} - 1
\end{aligned}$$

Proof. Note that $x_i - \mu = V_i + e^{-\eta d_i}(x_{i-1} - \mu) = V_i + e^{-\eta d_i}(W_i + e^{-\eta d_i}(x_i - \mu))$ so $(x_{i-1} - \mu)(1 - e^{-2\eta d_i}) = W_i + V_i e^{-\eta d_i}$.

We first calculate

$$\frac{\partial V_i}{\partial \eta} = d_i(x_{i-1} - \mu)e^{-\eta d_i} = d_i e^{-\eta d_i}\frac{W_i + e^{-\eta d_i}V_i}{1-e^{-2\eta d_i}}$$

then

$$
\begin{aligned}
\frac{\partial}{\partial \eta}\left[\frac{V_i}{1-e^{-2\eta d_i}}\right] =& \frac{\frac{\partial V_i}{\partial \eta}}{1-e^{-2\eta d_i}} - \frac{V_i \frac{\partial(1-e^{-2\eta d_i})}{\partial \eta}}{(1-e^{-2\eta d_i})^2} \\
=& \left[\frac{d_i(W_i + e^{-\eta d_i}V_i)e^{-\eta d_i}}{(1-e^{-2\eta d_i})^2}\right] - 2\left[\frac{V_i d_i e^{-2\eta d_i}}{(1-e^{-2\eta d_i})^2}\right] \\
=& d_i e^{-\eta d_i}\left[\frac{(W_i - e^{-\eta d_i}V_i)}{(1-e^{-2\eta d_i})^2}\right]
\end{aligned}
$$

By a symmetric argument

$$
\frac{\partial}{\partial \eta}\left[\frac{W_i}{1-e^{-2\eta d_i}}\right] = d_i e^{-\eta d_i}\left[\frac{(V_i - e^{-\eta d_i}W_i)}{(1-e^{-2\eta d_i})^2}\right]
$$

also

$$
\begin{aligned}
\frac{\partial}{\partial \eta}\left[\frac{V_i^2}{1-e^{-2\eta d_i}}\right] =& \frac{2V_i\frac{\partial}{\partial \eta}V_i}{1-e^{-2\eta d_i}} - \frac{V_i^2 \frac{\partial(1-e^{-2\eta d_i})}{\partial \eta}}{(1-e^{-2\eta d_i})^2} \\
=& 2V_i e^{-\eta d_i} d_i \left[\frac{W_i + e^{-\eta d_i}V_i}{(1-e^{-2\eta d_i})^2}\right] - 2\left[\frac{d_i e^{-2\eta d_i}V_i^2}{(1-e^{-2\eta d_i})^2}\right] \\
=& \frac{2d_i e^{-\eta d_i}}{(1-e^{-2\eta d_i})^2} V_i W_i
\end{aligned}
$$

so

$$
\begin{aligned}
&\frac{\partial^2}{\partial \eta^2}\left[\frac{V_i^2}{1-e^{-2\eta d_i}}\right] \\
=& 2d_i \frac{\partial}{\partial \eta}\left[e^{-\eta d_i}\left(\frac{V_i}{1-e^{-2\eta d_i}}\right)\left(\frac{W_i}{1-e^{-2\eta d_i}}\right)\right] \\
=& 2d_i e^{-\eta d_i}\left[-\frac{V_i W_i d_i}{(1-e^{-2\eta d_i})^2} + \left(\frac{V_i}{1-e^{-2\eta d_i}}\right)\frac{\partial}{\partial \eta}\left(\frac{W_i}{1-e^{-2\eta d_i}}\right) + \left(\frac{W_i}{1-e^{-2\eta d_i}}\right)\frac{\partial}{\partial \eta}\left(\frac{V_i}{1-e^{-2\eta d_i}}\right)\right] \\
=& \frac{2d_i^2 e^{-\eta d_i}}{(1-e^{-2\eta d_i})^3}\left[-V_i W_i(1-e^{-2\eta d_i}) + V_i e^{-\eta d_i}\left(V_i - W_i e^{-\eta d_i}\right) + W_i e^{-\eta d_i}\left(W_i - V_i e^{-\eta d_i}\right)\right] \\
=& \frac{2d_i^2 e^{-\eta d_i}}{(1-e^{-2\eta d_i})^3}\left[e^{-\eta d_i}(V_i{}^2 + W_i{}^2) - V_i W_i(1+e^{-2\eta d_i})\right]
\end{aligned}
$$

It is also easy to compute

$$\frac{\partial V_i}{\partial \mu} = e^{-\eta d_i} - 1$$

□

We now compute the expectations of the relevant terms.

Lemma 6. *If $\boldsymbol{x}$ follows an OU process with parameters μ, η and σ, and we set $V_i = (x_i - \mu) - (x_{i-1} - \mu)e^{-\eta d_i}$ and $W_i = (x_{i-1} - \mu) - (x_i - \mu)e^{-\eta d_i}$.*

$$\begin{aligned}
\mathbb{E}(V_i) = \mathbb{E}(W_i) &= 0 \\
\mathbb{E}(V_i^2) = \mathbb{E}(W_i^2) &= \frac{\sigma^2}{2\eta}(1 - e^{-2\eta d_i}) \\
\mathbb{E}(V_i W_i) &= -\frac{\sigma^2 e^{-\eta d_i}}{2\eta}(1 - e^{-2\eta d_i})
\end{aligned}$$

Proof. Recall that for an OU process, we have $\mathbb{E}(x_i) = \mu$, $\operatorname{Var}(x_i) = \frac{\sigma^2}{2\eta}$ and $\operatorname{Cov}(x_{i-1}, x_i) = \frac{\sigma^2}{2\eta} e^{-\eta d_i}$. It is therefore easy to see that $\mathbb{E}(V_i) = 0$ and $\mathbb{E}(W_i) = 0$. Now we can calculate

$$\begin{aligned}
\operatorname{Var}(V_i) &= \operatorname{Var}((x_i - \mu) - (x_{i-1} - \mu)e^{-\eta d_i}) \\
&= \operatorname{Var}(x_i - \mu) + \operatorname{Var}((x_{i-1} - \mu)e^{-\eta d_i}) - 2\operatorname{Cov}((x_i - \mu), (x_{i-1} - \mu)e^{-\eta d_i}) \\
&= \frac{\sigma^2}{2\eta} + \frac{\sigma^2}{2\eta} e^{-2\eta d_i} - 2e^{-\eta d_i} \frac{\sigma^2}{2\eta} e^{-\eta d_i} \\
&= \frac{\sigma^2}{2\eta}(1 - e^{-2\eta d_i})
\end{aligned}$$

The computation for $\operatorname{Var}(W_i)$ is the same. Finally,

$$\begin{aligned}
\operatorname{Cov}(V_i, W_i) &= -\operatorname{Cov}((x_i - \mu) - (x_{i-1} - \mu)e^{-\eta d_i}, (x_i - \mu)e^{-\eta d_i} - (x_{i-1} - \mu)) \\
&= -e^{-\eta d_i} \operatorname{Var}(x_i) - e^{-\eta d_i} \operatorname{Var}(x_{i-1}) + (1 + e^{-2\eta d_i}) \operatorname{Cov}(x_i, x_{i-1}) \\
&= \frac{\sigma^2 e^{-\eta d_i}}{2\eta}(1 + e^{-2\eta d_i}) - \frac{\sigma^2 e^{-\eta d_i}}{2\eta} - \frac{\sigma^2 e^{-\eta d_i}}{2\eta} \\
&= -\frac{\sigma^2 e^{-\eta d_i}}{2\eta}(1 - e^{-2\eta d_i})
\end{aligned}$$

□

Lemma 7. *If $\boldsymbol{x}$ follows an OU process with parameters μ, η and σ,*

$$\begin{aligned}
\mathbb{E}\left(\frac{\partial}{\partial\eta}\left[\frac{V_i}{1-e^{-2\eta d_i}}\right]\right) =& 0\\
\mathbb{E}\left(\frac{\partial}{\partial\eta}\left[\frac{V_i^2}{1-e^{-2\eta d_i}}\right]\right) =& -\frac{\sigma^2 d_i e^{-2\eta d_i}}{\eta(1-e^{-2\eta d_i})}\\
\mathbb{E}\left(\frac{\partial^2}{\partial\eta^2}\left[\frac{V_i^2}{1-e^{-2\eta d_i}}\right]\right) =& \frac{\sigma^2 d_i^2 e^{-2\eta d_i}\left(3+e^{-2\eta d_i}\right)}{\eta(1-e^{-2\eta d_i})^2}\\
\mathbb{E}\left(\frac{\partial V_i}{\partial\mu}\right) =& e^{-\eta d_i}-1
\end{aligned}$$

Proof. This is a straightforward substitution of the results in Lemma 6 into the results of Lemma 5. □

Proposition 8 (Proposition 1 in the main paper)**.** *For an OU process with parameter vector $\theta = (\mu, \eta, \sigma)^T$, sampled at time points $t_0 = 0, t_1, \ldots, t_n$, the Fisher information matrix is given by*

$$I = \begin{bmatrix} \frac{2\eta}{\sigma^2}\sum_{i=1}^n \frac{e^{\frac{s_i}{2}}-1}{e^{\frac{s_i}{2}}+1} & 0 & 0 \\ 0 & \frac{1}{4\eta^2}\sum_{i=1}^n \left({b_i}^2(e^{s_i}+1)-4b_i+2\right) & \frac{1}{\sigma\eta}\sum_{i=1}^n(b_i-1) \\ 0 & \frac{1}{\sigma\eta}\sum_{i=1}^n(b_i-1) & \frac{2n}{\sigma^2} \end{bmatrix}$$

where $s_i = 2\eta(t_i - t_{i-1})$ and $b_i = \frac{s_i}{e^{s_i}-1}$.

Proof.

$$\frac{\partial}{\partial\mu} l_{\text{OU}}(\boldsymbol{x};\mu,\eta,\sigma) = \frac{2\eta}{\sigma^2}\sum_{i=1}^n \frac{V_i(1-e^{-\eta d_i})}{1-e^{-2\eta d_i}} \tag{10}$$

$$\frac{\partial}{\partial\sigma} l_{\text{OU}}(\boldsymbol{x};\mu,\eta,\sigma) = -\frac{n}{\sigma} + \frac{2\eta}{\sigma^3}\sum_{i=1}^n \frac{V_i^2}{1-e^{-2\eta d_i}} \tag{11}$$

The first row comes by differentiating Equation (10) and taking the expectation. The third row comes by differentiating Equation (11) and taking the

expectation. Finally, for the second derivative with respect to η, we have

$$
\begin{aligned}
&\mathbb{E}\left(\frac{\partial^2 l_i(\boldsymbol{x};\mu,\eta,\sigma)}{\partial\eta^2}\right)\\
=&-\frac{1}{2\eta^2}-\frac{1}{2}\left(\frac{\partial^2\log(1-e^{-2\eta d_i})}{\partial\eta^2}+\frac{4}{\sigma^2}\mathbb{E}\left(\frac{\partial}{\partial\eta}\frac{V_i^2}{1-e^{-2\eta d_i}}\right)+\frac{2\eta}{\sigma^2}\mathbb{E}\left(\frac{\partial^2}{\partial\eta^2}\frac{V_i^2}{(1-e^{-2\eta d_i})}\right)\right)\\
=&-\frac{1}{2\eta^2}-\frac{1}{2}\left(-\frac{4d_i^2e^{-2\eta d_i}}{(1-e^{-2\eta d_i})^2}-\frac{4d_ie^{-2\eta d_i}}{\eta(1-e^{-2\eta d_i})}+\frac{2d_i^2e^{-2\eta d_i}\left(3+e^{-2\eta d_i}\right)}{(1-e^{-2\eta d_i})^2}\right)\\
=&-\frac{1}{2\eta^2}+\frac{1}{2}\left(\frac{4d_ie^{-2\eta d_i}}{\eta(1-e^{-2\eta d_i})}-\frac{2d_i^2e^{-2\eta d_i}(1+e^{-2\eta d_i})}{(1-e^{-2\eta d_i})^2}\right)\\
=&-\frac{1}{4\eta^2}\left(2-4\frac{s_i}{e^{s_i}-1}+\frac{s_i^2(e^{s_i}+1)}{(e^{s_i}-1)^2}\right)\\
&=-\frac{1}{4\eta^2}\left(2-4b_i+b_i^2(e^{s_i}+1)\right)
\end{aligned}
$$

□

B.2 Optimal Sampling Scheme

Theorem 9 (Theorem 3 in the main paper)**.** *1. The optimal sampling scheme to minimise* $\mathrm{Var}(\hat{\eta})$ *under an OU process is to sample the observations with time difference* $d_i = t_i - t_{i-1}$ *infinitesimal with probability* p *and equal to* $\frac{s^\dagger}{2\eta}$ *with probability* $1-p$*, where* $s^\dagger$ *and* p *are the solution to*

$$2\left(\frac{1}{s^\dagger}-\frac{1}{e^{s^\dagger}-1}\right)-1=2s^\dagger\left(\frac{1}{s^\dagger}-\frac{1}{e^{s^\dagger}-1}\right)^2\left(\frac{1}{s^\dagger}-\frac{1}{e^{s^\dagger}-1}-1\right)$$

and

$$p=\frac{1}{2}-\frac{{s^\dagger}^2(e^{s^\dagger}-1)}{4(e^{s^\dagger}-1-s^\dagger)^2}$$

Numerically these values can be solved as $s^\dagger = 1.956493$ *and* $p = 0.1572033$*. For this optimal sampling scheme,* $\mathrm{Var}(\hat{\eta}) = \frac{6.12679\eta^2}{n}$*.*

2. Let s_l *be the solution to*

$$s_l^2(e^{s_l}-1)-c_0(e^{s_l}-1)^2=2(b_0(e^{s_l}-1)-s_l)^2$$

with $c_0 = \sup \frac{x^2}{e^x-1} = 0.6476102$ *and* $b_0 = 1 - \sqrt{1-c_0} = 0.4063757$. *Numerically, this is* $s_l = 0.5844618$. *If samples from an OU process must be collected with time difference* $d_i \geqslant \frac{s_l}{2\eta}$, *then the optimal sampling scheme is to sample evenly-spaced observations with* $d_i = \frac{s_0}{2\eta}$, *where* s_0 *is the solution to*

$$(2 - s_0)(e^{s_0} - 1) = s_0$$

Numerically, $s_0 = 1.59362426$, *and for this sampling scheme* $\mathrm{Var}(\hat{\eta}) = \frac{6.176555\eta^2}{n}$.

3. *If* σ *is known for an OU process, then the optimal sampling scheme to minimise* $\mathrm{Var}(\hat{\eta})$ *is to sample evenly-spaced observations with time difference* $d_i = \frac{s_k}{2\eta}$ *where* s_k *is the solution to*

$$4(e^{s_k} - 1)^2 + s_k^2 e^{s_k}(3 + e^{s_k}) = 6 s_k e^{s_k}(e^{s_k} - 1) + 2 s_k (e^{s_k} - 1)$$

Numerically, we get $s_k = 5.109858$. *In this case* $\mathrm{Var}(\hat{\eta}) = \frac{1.964279\eta^2}{n}$.

Proof. Recall that the variance of the MLEs is asymptotically given by the inverse of the Fisher information matrix. Since the matrix is block diagonal, we can invert the blocks separately, so $(I^{-1})_{\eta\eta} = \frac{\frac{2n}{\sigma^2}}{|I_{(\eta,\sigma)(\eta,\sigma)}|}$. Therefore

$$\begin{aligned}
2\eta^2((I^{-1})_{\eta\eta})^{-1} &= \frac{\eta^2\sigma^2}{n}|I_{(\eta,\sigma)(\eta,\sigma)}| \\
&= \frac{\eta^2\sigma^2}{n}\left(\frac{2n}{\sigma^2}\left(\frac{1}{4\eta^2}\sum_{i=1}^{n}\left({b_i}^2(e^{s_i}+1) - 4b_i + 2\right)\right) - \left(\frac{1}{\sigma\eta}\sum_{i=1}^{n}(b_i - 1)\right)^2\right) \\
&= \frac{1}{2}\sum_{i=1}^{n}\left(b_i^2(1+e^{s_i}) - 4b_i + 2\right) - \frac{1}{n}\left(\sum_{i=1}^{n}(b_i-1)\right)^2 \\
&= n\left(\frac{1}{n}\sum_{i=1}^{n}(b_i-1)^2 - \left(\frac{1}{n}\sum_{i=1}^{n}(b_i-1)\right)^2\right) + \frac{1}{2}\sum_{i=1}^{n}{b_i}^2(e^{s_i}-1) \\
&= n\left(\frac{1}{n}\sum_{i=1}^{n}{b_i}^2 - \left(\frac{1}{n}\sum_{i=1}^{n}b_i\right)^2\right) + \frac{1}{2}\sum_{i=1}^{n}b_i s_i
\end{aligned}$$

Where the last step is because the first term is the variance of $b_i - 1$ over the observations, which is equal to the variance of b_i, since variance is translation-invariant. More formally, we let D be a random variable which takes each value d_i with probability $\frac{1}{n}$, and let $S = 2\eta D$, $B = \frac{S}{e^S - 1}$ and $C = \frac{S^2}{e^S - 1}$. We have therefore shown that

$$((I^{-1})_{\eta\eta})^{-1} = \frac{n}{2\eta^2}\left(\mathrm{Var}\,(B) + \frac{1}{2}\mathbb{E}\,(C)\right)$$

Now we can prove the optimality results in parts 1–3

1. We will show that $\mathrm{Var}\,(B) + \frac{1}{2}\mathbb{E}\,(C)$ is maximised when S is a distribution with support a two-element set $\{0, s^\dagger\}$, with probabilities p and $1-p$ respectively. In practice, the distribution S must be discrete with probability masses multiples of $\frac{1}{n}$, but for large n, this can be arbitrarily close to the optimal distribution, so we will simply solve the problem of the asymptotic distribution.

We will first show that there is a unique non-zero point $s^\dagger$ in the support of the optimal S. To prove this, it will be convenient to define the functions $a(x) = \frac{1}{e^x - 1}$, $b(x) = \frac{x}{e^x - 1}$ and $c(x) = \frac{x^2}{e^x - 1}$. For a distribution S to be optimal, $\mathrm{Var}(b(S)) + \frac{1}{2}\mathbb{E}(c(S))$ must be larger than for any other distribution $\tilde{S}$. Letting $\tilde{B} = b(\tilde{S})$, $\tilde{C} = c(\tilde{S})$ and $b^* = \mathbb{E}(B)$, we have

$$\begin{aligned}\mathbb{E}(2(B - b^*)^2 + C) &\geqslant \mathbb{E}(2(\tilde{B} - \mathbb{E}(\tilde{B}))^2 + \tilde{C}) \\ &= \mathbb{E}(2(\tilde{B} - b^*)^2 + \tilde{C}) - 2(\mathbb{E}(\tilde{B}) - b^*)^2\end{aligned}$$

$$\mathbb{E}(2(\tilde{B} - b^*)^2 + \tilde{C}) - \mathbb{E}(2(B - b^*)^2 + C) \leqslant 2(\mathbb{E}(\tilde{B}) - b^*)^2$$

In particular, for s in the support of S and $\epsilon > 0$, we let

$$E_{s,\epsilon} = \begin{cases} \epsilon & \text{if } |S - s| \leqslant \epsilon \\ 0 & \text{otherwise} \end{cases}$$

then, letting p_ϵ denote $P(|S - s| < \epsilon)$, and letting b, c, b' and c' denote $b(s)$, $c(s)$, $\left.\frac{db(x)}{dx}\right|_{x=s}$ and $\left.\frac{dc(x)}{dx}\right|_{x=s}$ respectively, we get

$$\begin{aligned}\mathbb{E}(b(S + E_{s,\epsilon})) &= \mathbb{E}(B) + p_\epsilon(\epsilon b' + o(\epsilon)) \\ \mathbb{E}(2(b(S + E_{s,\epsilon}) - b^*)^2 + c(S + E_{s,\epsilon})) &= \mathbb{E}(2(B - b^*)^2 + C) + p_\epsilon(\epsilon(2(b - b^*)b' + c') + o(\epsilon))\end{aligned}$$

Thus

$$\begin{aligned}
p_\epsilon(\epsilon(2(b-b^*)b'+c')+o(\epsilon)) &\leqslant \left(p_\epsilon(\epsilon b'+o(\epsilon))\right)^2\\
\epsilon(2(b-b^*)b'+c')+o(\epsilon) &\leqslant p_\epsilon(\epsilon b'+o(\epsilon))^2\\
&= p_\epsilon(\epsilon^2 b'^2+o(\epsilon^2))\\
&= O(\epsilon^2)
\end{aligned}$$

We see that as $\epsilon \to 0$, this can only happen if $2(b-b^*)b'+c' \leqslant 0$. Similarly, considering $S - E_{d,\epsilon}$, we get $2(b-b^*)b'+c' \geqslant 0$. Thus s must satisfy the equation $2(b-b^*)b'+c' = 0$, or

$$\begin{aligned}
2(b-b^*)(a-b(a+1))+2b-c(a+1) &= 0\\
2(b-b^*)(a-b(a+1))+b+s(a-b(a+1)) &= 0\\
(2(b-b^*)+s)(a-b(a+1))+b &= 0\\
(2(b-b^*)+s)\left(a+1-\frac{1}{s}\right) &= 1 \qquad (12)
\end{aligned}$$

where $a = a(s)$. We see that $2b+s-2b^* = s\frac{e^s+1}{e^s-1} - 2b^*$ and $a+1-\frac{1}{s}$ are both increasing functions of s, (since $\frac{d}{ds}\left(a+1-\frac{1}{s}\right) = \frac{1}{s^2} - \frac{e^s}{(e^s-1)^2} = \left(\frac{1}{s}+\frac{1}{e^{\frac{s}{2}}-e^{-\frac{s}{2}}}\right)\left(\frac{1}{s}-\frac{1}{e^{\frac{s}{2}}-e^{-\frac{s}{2}}}\right) > 0$) and $a+1-\frac{1}{s}$ is positive, so there can be at most one solution to this equation.

We will let $s^\dagger$ denote this solution. Since this is the only internal point in the support of S, the support of S must consist of the two points $s^\dagger$ and 0. Thus,

$$S = \begin{cases} 0 & \text{with probability } p\\ s^\dagger & \text{with probability } 1-p \end{cases}$$

We need to maximise

$$2\operatorname{Var}(B) + \mathbb{E}(C) = 2p(1-p)(1-b^\dagger)^2 + (1-p)c^\dagger$$

over p and $s^\dagger$, where $b^\dagger = b(s^\dagger)$ and $c^\dagger = c(s^\dagger)$. (We have used that $b(0) = 1$ and $c(0) = 0$.)

$$\begin{aligned}
\frac{\partial}{\partial p}\left(2\operatorname{Var}_D(B) + \mathbb{E}_D(C)\right) &= (2-4p)(1-b^\dagger)^2 - c^\dagger\\
\frac{\partial}{\partial s^\dagger}\left(2\operatorname{Var}_D(B) + \mathbb{E}_D(C)\right) &= (1-p)c'^\dagger - 4p(1-p)(1-b^\dagger)b'^\dagger\\
&= (1-p)(2b^\dagger - c^\dagger(a^\dagger+1)) - 4p(1-p)(1-b^\dagger)(a^\dagger - b^\dagger(a^\dagger+1))
\end{aligned}$$

where $a^\dagger = a(s^\dagger)$. We set both of these partial derivatives to 0 and solve.

$$\begin{aligned}
(2-4p)(1-b^\dagger)^2 &= c^\dagger \\
(1-p)(2b^\dagger - c^\dagger(a^\dagger+1)) &= 4p(1-p)(1-b^\dagger)(a^\dagger - b^\dagger(a^\dagger+1)) \\
4p &= 2 - \frac{c^\dagger}{(1-b^\dagger)^2} \\
2b^\dagger - c^\dagger(a^\dagger+1) &= \left(2 - \frac{c^\dagger}{(1-b^\dagger)^2}\right)(1-b^\dagger)(a^\dagger - b^\dagger(a^\dagger+1)) \\
&= \left(2 - 2b^\dagger - \frac{c^\dagger}{(1-b^\dagger)}\right)(a^\dagger(1-b^\dagger) - b^\dagger) \\
&= 2a^\dagger(1-b^\dagger)^2 - a^\dagger c^\dagger - 2b^\dagger(1-b^\dagger) + \frac{b^\dagger c^\dagger}{1-b^\dagger} \\
2b^\dagger - c^\dagger - \frac{b^\dagger c^\dagger}{1-b^\dagger} &= 2(1-b^\dagger)(a^\dagger(1-b^\dagger) - b^\dagger) \\
2b^\dagger(1-b^\dagger) - c^\dagger &= 2(1-b^\dagger)^2(a^\dagger(1-b^\dagger) - b^\dagger) \\
2a^\dagger b^\dagger(1-b^\dagger) - {b^\dagger}^2 &= 2a^\dagger(1-b^\dagger)^2(a^\dagger(1-b^\dagger) - b^\dagger)
\end{aligned}$$

Substituting $r^\dagger = \frac{a^\dagger(1-b^\dagger)}{b^\dagger} = \frac{1-b^\dagger}{s^\dagger} = \frac{1}{s^\dagger} - a^\dagger$ and dividing by ${b^\dagger}^2$, this equation becomes

$$2r^\dagger - 1 = 2(1-b^\dagger)r^\dagger(r^\dagger - 1) = 2s^\dagger {r^\dagger}^2(r^\dagger - 1)$$

We compute

$$\frac{dr}{ds} = a(a+1) - \frac{1}{s^2}$$

Therefore,

$$\begin{aligned}
&\frac{d}{ds}\big(2sr^2(r-1) - 2r + 1\big) \\
&\qquad = 2\left(r^2(r-1) + rs\left(a(a+1) - \frac{1}{s^2}\right)(3r-2) - \left(a(a+1) - \frac{1}{s^2}\right)\right)
\end{aligned}$$

From this, we can use Newton's method to find $s^\dagger$ numerically. Numerically, we solve $s^\dagger = 1.956493$. This corresponds to $p = 0.1572033$. Thus, the optimal sampling scheme under an OU process is to sample 0.1572033 of the

observations with almost no time difference, and the remaining 0.8427967 of the observations with a time difference $d_i = \frac{1.956493}{2\eta}$.

From this, we compute $b^\dagger = 0.3220844$ and $c^\dagger = 0.6301558$. Therefore, we have

$$\mathrm{Var}\left(\frac{\hat{\eta}}{\eta}\right) = \frac{2}{n}\left(2\,\mathrm{Var}(B) + \mathbb{E}(C)\right)^{-1} = \frac{2}{n}\left(2p(1-p)(1-b^\dagger)^2 + (1-p)c^\dagger\right)^{-1} = \frac{6.12679}{n}$$

2. We are trying to maximise $\mathrm{Var}(B) + \frac{1}{2}\mathbb{E}(C)$. We see that s_l is the solution to $c_0 = c(s_l) + 2\left(b(s_l) - b_0\right)^2$. Thus for $s > s_l$, we have $c(s) + 2\left(b(s) - b_0\right)^2 \leqslant c_0$, so $\mathrm{Var}(B) + \frac{1}{2}\mathbb{E}(C) \leqslant \mathbb{E}\left((B-b_0)^2 + \frac{1}{2}C\right) \leqslant c_0$, with equality only when $S = s_0$ with probability 1.

3. When σ is known, the variance of $\hat{\eta}$ is given by

$$(I_{\eta\eta})^{-1} = 4\eta^2 \left(\sum_{i=1}^n \left({b_i}^2(e^{s_i}+1) - 4b_i + 2\right)\right)^{-1}$$

so the variance is minimised by maximising

$$\sum_{i=1}^n \left({b_i}^2(e^{s_i}+1) - 4b_i + 2\right)$$

It is easy to see that all terms in this sum are maximised by the same s_i, so the optimal solution is to set all s_i to this value, which is found by solving

$$\begin{aligned}
\frac{\partial}{\partial s}\left(b^2(e^s+1) - 4b + 2\right) &= 0\\
2b(e^s+1)(a - b(a+1)) + b^2e^s - 4(a - b(a+1)) &= 0\\
2s\frac{e^s+1}{e^s-1}\left(\frac{1}{e^s-1} - \frac{se^s}{(e^s-1)^2}\right) + \frac{s^2e^s}{(e^s-1)^2} - 4\left(\frac{1}{e^s-1} - \frac{se^s}{(e^s-1)^2}\right) &= 0\\
2s(e^s+1)\left(e^s - 1 - se^s\right) + s^2e^s(e^s-1) - 4\left((e^s-1)^2 - se^s(e^s-1)\right) &= 0\\
4(e^s-1)^2 - 6se^s(e^s-1) - 2s\left(e^s - 1 - se^s\right) + 2s^2e^{2s} - s^2e^s(e^s-1) &= 0\\
4(e^s-1)^2 - 6se^s(e^s-1) - 2s\left(e^s - 1 - se^s\right) + s^2e^{2s} + s^2e^s &= 0\\
4(e^s-1)^2 - 6se^s(e^s-1) - 2s\left(e^s-1\right) + s^2e^s(e^s+3) &= 0
\end{aligned}$$

□

C Compositionality Simulations

In this section, we use simulations to study the issues caused by compositionality and the various methods to correct it. The issue is that when the data are normalised to deal with sequencing depth noise, the behaviour of a single genus will have effects on all other genera, so if a single genus fails to follow Brownian motion, for example, looking at relative abundance might cause us to reject Brownian motion for other genera. We perform a simulation to study this effect.

C.1 SimulationDesign

We perform a simulation with 10 genera. We let the log abundance of each genus independently follow an OU process with certain parameters. We then sample the observed counts as following a Poisson distribution with mean given by the abundance of this genus. After this, we apply normalisation to the simulated count data. We compare three different approaches to normalisation: log-proportions, where we divide each observed count by the total count for that sample, then take the logarithm of the proportion; centred log-ratio (CLR) where we take the logarithm of each observed count, then centralise these logarithms; pairwise log ratio, where we take the ratio of the count for each pair, and take the logarithm of this.

We analyse a number of different issues: we assess the power and size of our likelihood ratio tests using different normalisation approaches.

In each scenario, we simulate 300 timepoints with 60% of time differences 1 day, 30% 3 days and 10% 10 days. We simulate 10 genera. For each genus, σ is drawn from a scaled chi distribution with one degree of freedom, with scale equal to $\frac{\eta+1}{32}$. The reason we let the scale increase with η is that the long-term variance of the OU process is $\frac{\sigma^2}{2\eta}$, so if the scale is constant, then the variance will be smaller for large η.

We simulate the following scenarios:

- All genera follow Brownian motion. Initial log-abundances are normally distributed with mean 8 and variance 1.
- Half of the genera follow Brownian motion. The other half follow an OU process with $\eta = 1$. We set $\mu = 2, 3, 5, 6, 9$ for the mean-reverting

- All genera follow an OU process with $\eta = 0.6, 0.7, \ldots, 1.4, 1.5$ and $\mu = 6, 9, 6, 9, 6, 9, 6, 9, 6, 9$.
- Half of the genera follow an OU process with $\eta = 1$ and $\mu = 2, 3, 5, 6, 9$. The other half are i.i.d. normal with mean $2, 3, 4, 6, 8$.

There are two issues that might be influencing the accuracy of our methods: the compositionality of the data and the fact that the data are count data, rather than continuous measurements of abundance. To separate the influence of these issues, we also perform the same simulations without the Poisson noise. That is, we directly analyse the simulated abundances.

C.2 Simulation Results

C.2.1 Scenario 1

Table 1 shows the average estimated $\hat{\eta}$ values for each genus over 100 simulations, under an OU process and the proportion of simulations which reject a null hypothesis of Brownian motion for various normalisation methods. We note the following features of these results:

- All methods reject Brownian motion more frequently than the nominal size of the test.
- Log-proportions reject Brownian motion least frequently.
- Estimated η values are small for all methods, so even though the methods sometimes suggest that there is mean reversion, the estimated effect is not strong.
- While the rejection rates are higher than the nominal rates, they are still relatively small, so we would not expect results like the real data, where Brownian motion is rejected for all genera, if there were no mean reversion.

C.2.2 Scenario 2

Table 2 shows the average estimated $\hat{\eta}$ values for each genus over 100 simulations, under an OU process and the proportion of simulations which reject a null hypothesis of Brownian motion for various normalisation methods. In

Table 1: Comparison of normalisation schemes: Scenario 1 with Poisson noise.
Results are averaged over 100 simulations. True dynamics follow Brownian motion with Poisson noise. Initial abundances for each genus are i.i.d. log-normal with $\mu = 8$ and $\sigma = 1$.

(a) log-proportion and CLR, estimated η

Genus	η	log-prop	CLR
1	0	0.0154	0.0215
2	0	0.0176	0.0223
3	0	0.0198	0.0235
4	0	0.0212	0.0311
5	0	0.0170	0.0189
6	0	0.0215	0.0287
7	0	0.0215	0.0277
8	0	0.0169	0.0223
9	0	0.0163	0.0229
10	0	0.0208	0.0231

(b) Pairwise log ratio, estimated η

	2	3	4	5	6	7	8	9	10
1	0.097	0.025	0.024	0.028	0.026	0.030	0.030	0.028	0.030
2		0.020	0.024	0.046	0.028	0.026	0.058	0.019	0.029
3			0.026	0.020	0.023	0.030	0.024	0.022	0.040
4				0.032	0.037	0.032	0.032	0.052	0.028
5					0.020	0.024	0.017	0.023	0.018
6						0.027	0.034	0.022	0.048
7							0.036	0.038	0.030
8								0.023	0.057
9									0.029

(c) log-proportion and CLR, proportion that reject BM

Genus	η	log-prop	CLR
1	0	0.17	0.23
2	0	0.24	0.29
3	0	0.30	0.32
4	0	0.25	0.37
5	0	0.18	0.28
6	0	0.27	0.21
7	0	0.26	0.33
8	0	0.27	0.30
9	0	0.23	0.37
10	0	0.23	0.33

(d) Pairwise log ratio, proportion that reject BM

	2	3	4	5	6	7	8	9	10
1	0.34	0.26	0.34	0.23	0.23	0.31	0.22	0.28	0.33
2		0.28	0.25	0.31	0.28	0.2	0.3	0.28	0.29
3			0.27	0.3	0.24	0.32	0.3	0.3	0.33
4				0.35	0.28	0.29	0.28	0.34	0.3
5					0.27	0.22	0.21	0.28	0.2
6						0.3	0.29	0.26	0.31
7							0.25	0.31	0.32
8								0.24	0.29
9									0.33

Table 2: Comparison of normalisation schemes: Scenario 2 with Poisson noise Results are averaged over 100 simulations. Brownian motion for some genera and an OU process for others. All genera are subject to Poisson noise. Initial abundances for each genus are i.i.d. log-normal with $\mu = 8$ and $\sigma = 1$. Cells highlighted in yellow follow Brownian motion, so Brownian motion should not be rejected and the estimated value of η should be zero. Cells highlighted in blue are pairwise log-ratios where one genus follows Brownian motion and the other follows an OU process.

(a) log-proportion and CLR, estimated η

Genus	η	μ	log-prop	CLR
1	0		0.017	0.090
3	0		0.023	0.095
5	0		0.017	0.084
7	0		0.024	0.116
9	0		0.017	0.084
2	1	2	0.937	1.460
4	1	3	0.632	1.100
6	1	5	0.281	0.550
8	1	6	0.175	0.375
10	1	9	0.157	0.543

(b) pairwise log ratio, estimated η

	3	5	7	9	2	4	6	8	10
1	0.014	0.014	0.014	0.014	0.777	0.523	0.292	0.215	0.158
3		0.012	0.015	0.013	0.794	0.519	0.274	0.206	0.146
5			0.012	0.013	0.732	0.473	0.253	0.19	0.115
7				0.018	0.809	0.549	0.31	0.217	0.14
9					0.733	0.474	0.255	0.188	0.106
2						3.693	2.544	2.227	1.453
4							2.53	2.073	1.264
6								2.001	1.146
8									1.127

(c) log-proportion and CLR, proportion that reject BM

Genus	η	μ	log-prop	CLR
1	0		0.25	0.77
3	0		0.32	0.74
5	0		0.21	0.76
7	0		0.22	0.75
9	0		0.26	0.73
2	1	2	1.00	1.00
4	1	3	1.00	1.00
6	1	5	0.98	1.00
8	1	6	0.90	1.00
10	1	9	0.70	1.00

(d) pairwise log ratio, proportion that reject BM

	3	5	7	9	2	4	6	8	10
1	0.11	0.11	0.15	0.13	1	0.99	0.93	0.82	0.63
3		0.14	0.18	0.18	1	0.98	0.91	0.8	0.56
5			0.14	0.16	1	0.99	0.92	0.81	0.65
7				0.2	1	0.99	0.89	0.77	0.65
9					1	0.99	0.9	0.78	0.63
2						1	1	1	1
4							1	1	1
6								1	1
8									1

this simulation, five of the genera follow Brownian motion, while the other five have mean reversion. One of the major concerns about normalisation is that mean reversion in some abundances causes mean reversion in the total abundance, which in turn leads to mean reversion in all normalised abundances, causing us to reject Brownian motion, even for genera without mean reversion. We note the following features of these results:

- The pairwise log-ratio results are even better than in Scenario 1, with a rejection rate closer to the nominal rate in cases where both genera follow Brownian motion.
- Pairwise log ratios have high power to reject Brownian motion in cases where only one of the genera has mean reversion.
- The CLR has been severly affected by the normalisation, producing very high false positive rates.
- Even in cases of false positives, the estimated mean reversion rates are low, suggesting that large estimates of η correspond to genuine mean reversion.
- Log-proportions have higher false positive rates than Pairwise log-ratios, but much lower than CLR.
- Log proportions have low power to reject Genus 10 in this simulation. Because the mean level of Genus 10 is larger than the other genera, Genus 10 will typically represent about $\frac{2}{3}$ of the total abundance (this will vary a lot between simulations, because the abundance of the genera under Brownian motion can vary a lot), so this is an extreme case of a genus affected by compositional effects.
- The presence of genera under Brownian motion has caused the log-proportions to underestimate the mean-reversion rate for the most abundant genera. This underestimation is most severe for the most abundant genera.

C.2.3 Scenario 3

Table 3 shows the average estimated $\hat{\eta}$ values for each genus over 100 simulations, under an OU process and the proportion of simulations which reject

Table 3: Comparison of normalisation schemes: Scenario 3 with Poisson noise.
Results are averaged over 100 simulations. True dynamics follow an OU process with Poisson noise. Initial abundances for each genus are i.i.d. log-normal with $\mu = 8$ and $\sigma = 1$.

(a) log-proportion and CLR, estimated η

Genus	η	μ	log-prop	CLR
1	0.6	6	0.63	0.75
2	0.7	9	1.14	0.82
3	0.8	6	0.81	1.05
4	0.9	9	1.37	0.91
5	1.0	6	0.99	1.31
6	1.1	9	1.83	1.11
7	1.2	6	1.18	1.59
8	1.3	9	2.17	1.25
9	1.4	6	1.32	1.95
10	1.5	9	2.47	1.36

(b) pairwise log ratio, estimated η

	2	3	4	5	6	7	8	9	10
1	0.705	1.244	0.748	1.299	0.807	1.412	0.849	1.597	0.908
2		0.863	0.879	1.068	0.966	1.121	1.060	1.281	1.290
3			0.913	1.497	1.042	1.887	1.028	1.988	1.092
4				1.041	1.067	1.169	1.186	1.279	1.372
5					1.163	1.825	1.195	1.755	1.261
6						1.352	1.262	1.388	1.424
7							1.340	1.892	1.406
8								1.488	1.564
9									1.531

(c) log-proportion and CLR, proportion that reject BM

Genus	η	μ	log-prop	CLR
1	0.6	6	1	1
2	0.7	9	1	1
3	0.8	6	1	1
4	0.9	9	1	1
5	1.0	6	1	1
6	1.1	9	1	1
7	1.2	6	1	1
8	1.3	9	1	1
9	1.4	6	1	1
10	1.5	9	1	1

(d) pairwise log ratio, proportion that reject BM

	2	3	4	5	6	7	8	9	10
1	1	1	1	1	1	1	1	1	1
2		1	1	1	1	1	1	1	1
3			1	1	1	1	1	1	1
4				1	1	1	1	1	1
5					1	1	1	1	1
6						1	1	1	1
7							1	1	1
8								1	1
9									1

a null hypothesis of Brownian motion for various normalisation methods. In this simulation, all of the genera have some mean reversion, but the rate of mean reversion and the mean level varies between genera. We note the following features of these results:

- All the methods are able to reject Brownian motion for all genera in all simulations.
- All methods tend towards overestimating mean-reversion rates.
- Pairwise log-ratios perform well when at least one genus is abundant ($\mu = 9$).
- Log-proportion estimates mean reversion rates well for less abundant genera, but overestimates rates for more abundant genera.
- For CLR, the estimates for abundant genera are fairly good, but are pushed towards the mean (so large rates are underestimated, while small rates are overestimated). CLR tends to overestimate mean reversion rates for less abundant genera.

C.2.4 Scenario 4

Table 4 shows the average estimated $\hat{\eta}$ values for each genus over 100 simulations, under an OU process and the proportion of simulations which reject a null hypothesis of i.i.d. samples for various normalisation methods. Half of the genera are simulated following a mean-reverting process with $\eta = 1$. The other half are simulated with $\eta = 2000$, effectively producing i.i.d. samples. We note the following features of these results:

- The false positive rate for pairwise log-ratios is at or below the nominal size of the test in cases where both genera are i.i.d. In cases where both genera follow an OU process, pairwise log-ratios have high power, particularly for abundant genera. In cases where one genus is i.i.d. and the other follows an OU process, the pairwise log ratio results in power that appears to decrease as the abundance of the genus that follows an OU process increases. In most cases, the power is still high.
- The false positive rate for Log-proportions is higher than the nominal rate, but still moderately well controlled. The power falls for the more abundant genera, but remains high.

Table 4: Comparison of normalisation schemes: Scenario 4 with Poisson noise.
Results are averaged over 100 simulations. , true dynamics are i.i.d. for some genera and follow an OU process for other genera, with Poisson noise for all genera. Initial abundances for each genus are i.i.d. log-normal with $\mu = 8$ and $\sigma = 1$ for genera following an OU process and are drawn from the stationary distribution for i.i.d. genera. Red cells are i.i.d. and should not reject the i.i.d. hypothesis and should estimate large values for η. Magenta cells are log-ratios between an i.i.d. sequence and a genus following an OU process. These should reject the i.i.d. hypothesis and estimate a smaller value of η.

(a) log-proportion and CLR, proportion that reject iid

Genus	η	μ	log-prop	CLR
1	∞	2	0.08	0.37
3	∞	3	0.13	0.47
5	∞	4	0.11	0.45
7	∞	6	0.15	0.52
9	∞	8	0.30	0.45
2	1	2	1.00	1.00
4	1	3	1.00	1.00
6	1	5	0.95	0.84
8	1	6	0.85	0.60
10	1	9	0.81	1.00

(b) pairwise log ratio, proportion that reject iid

	3	5	7	9	2	4	6	8	10
1	0.01	0.02	0.02	0.04	0.98	0.97	0.74	0.52	0.27
3		0.02	0	0.03	0.99	0.98	0.74	0.58	0.35
5			0	0.01	1	0.95	0.77	0.55	0.45
7				0	0.97	0.93	0.77	0.6	0.4
9					0.97	0.97	0.77	0.57	0.41
2						0.64	0.93	0.99	1
4							0.84	0.96	1
6								0.93	1
8									0.99

(c) log-proportion and CLR, estimated η

Genus	η	μ	log-prop	CLR
1	∞	2	4.79	4.48
3	∞	3	4.67	4.53
5	∞	4	4.52	4.28
7	∞	6	5.04	4.41
9	∞	8	4.67	4.17
2	1	2	1.95	2.18
4	1	3	1.93	2.14
6	1	5	2.27	3.26
8	1	6	2.76	3.84
10	1	9	3.76	2.16

(d) pairwise log ratio, estimated η

	3	5	7	9	2	4	6	8	10
1	4.66	4.61	4.91	4.67	3.16	3.57	4.11	4.37	4.65
3		4.73	4.94	4.61	3.24	3.31	3.84	4.21	4.35
5			4.78	4.47	2.88	3.08	3.48	3.91	4.33
7				5.1	3.19	3.31	3.72	4.23	4.38
9					2.9	3.2	3.55	3.78	4.11
2						3.65	2.81	2.4	1.49
4							2.73	2.02	1.26
6								2.03	1.15
8									1.1

- CLR has higher false positive rates than the other methods, but its power is similar to other methods.
- All methods overestimate the mean-reversion rate η for the genera following an OU process. For log-proportions, this overestimation is largest for the most abundant genera. For Pairwise log-ratios this overestimation is smallest for the most abundant genera. For CLR, the overestimation does not seem related to abundance of the genus, but seems larger overall than for log-proportions.

C.2.5 Summary of Results with Poisson Noise

- All methods reject Brownian motion more frequently than the nominal size.
- CLR has very high false positive rates when some genera should reject Brownian motion and some should not. Even when no genera should reject Brownian motion, the false positive rate of CLR is higher than the other normalisation methods.
- The false positive rate for testing Brownian motion using log-proportion normalisation is larger than the nominal size of the test, but remains around 20–30%, so in the majority of cases, using log-proportions will not cause us to falsely reject Brownian motion.
- Even in cases where Brownian motion is wrongly rejected, the estimated mean reversion rates remain low. Thus, in cases where the estimated mean reversion rate is large, we can be confident that Brownian motion should be rejected.
- For testing i.i.d. normality, the false positive rate using pairwise log ratios is smaller than the nominal size, while the power is high, except in cases where one abundant genus is under mean reversion and the other genus is i.i.d..
- The false positive rate for testing i.i.d. normal using log-proportion normalisation is larger than the nominal rate, but still not too large. It is larger for more abundant genera. The power is high, but drops slightly for the more abundant genera.

- CLR has a higher false positive rate for testing i.i.d. normality, and has similar power.

C.3 Without Poisson Noise

C.3.1 Scenario 1

Table 5 shows the average estimated $\hat{\eta}$ values for each genus over 100 simulations, under an OU process and the proportion of simulations which reject a null hypothesis of Brownian motion for various normalisation methods. The log-abundances of all genera are simulated following Brownian motion. We note the following features of these results:

- The false positive rates for all methods are similar to the nominal size of the test.
- The estimated mean-reversion rates are all very low.

C.3.2 Scenario 2

Table 6 shows the average estimated $\hat{\eta}$ values for each genus over 100 simulations, under an OU process and the proportion of simulations which reject a null hypothesis of Brownian motion for various normalisation methods. The log-abundances of odd genera are simulated following Brownian motion, while even genera follow an OU process with $\eta = 1$. We note the following features of these results:

- The false positive rates for pairwise log ratio in cases where both genera follow Brownian motion are similar to the nominal size of the test.
- In cases where at least one genus has mean reversion, the pairwise log-ratio method has high power.
- The false positive rates for log-proportions and CLR are higher than in the case with Poisson noise.
- The false positive rates for CLR are very high.
- For genera with mean reversion, both log-proportions and CLR have high power.

Table 5: Comparison of normalisation schemes: Scenario 1 without Poisson noise.
Results are averaged over 100 simulations. All genera follow Brownian motion. Initial abundances for each genus are i.i.d. log-normal with $\mu = 8$ and $\sigma = 1$.

(a) log-proportion and CLR, estimated η

Genus	η	log-prop	CLR
1	0	0.007	0.007
2	0	0.006	0.006
3	0	0.008	0.008
4	0	0.007	0.007
5	0	0.008	0.007
6	0	0.008	0.007
7	0	0.008	0.008
8	0	0.007	0.008
9	0	0.008	0.008
10	0	0.007	0.007

(b) Pairwise log ratio, estimated η

	2	3	4	5	6	7	8	9	10
1	0.007	0.007	0.007	0.007	0.007	0.008	0.007	0.008	0.007
2		0.007	0.006	0.006	0.006	0.006	0.007	0.007	0.006
3			0.007	0.008	0.007	0.008	0.007	0.008	0.007
4				0.008	0.006	0.007	0.007	0.009	0.007
5					0.008	0.007	0.007	0.008	0.007
6						0.008	0.008	0.007	0.008
7							0.007	0.009	0.009
8								0.007	0.007
9									0.008

(c) log-proportion and CLR, proportion that reject BM

Genus	η	log-prop	CLR
1	0	0.04	0.07
2	0	0.05	0.08
3	0	0.08	0.11
4	0	0.02	0.05
5	0	0.04	0.04
6	0	0.07	0.03
7	0	0.04	0.04
8	0	0.06	0.06
9	0	0.01	0.06
10	0	0.04	0.06

(d) Pairwise log ratio, proportion that reject BM

	2	3	4	5	6	7	8	9	10
1	0.05	0.06	0.06	0.04	0.06	0.05	0.03	0.08	0.07
2		0.07	0.04	0.04	0.08	0.02	0.05	0.08	0.05
3			0.08	0.06	0.07	0.07	0.08	0.12	0.08
4				0.1	0.04	0.02	0.04	0.06	0.07
5					0.03	0.03	0.04	0.07	0.04
6						0.03	0.11	0.03	0.06
7							0.06	0.08	0.12
8								0.04	0.06
9									0.07

Table 6: Comparison of normalisation schemes: Scenario 2 without Poisson noise.
Results are averaged over 100 simulations. Odd genera follow Brownian motion; even genera follow an OU process. Initial abundances for each genus are i.i.d. log-normal with $\mu = 8$ and $\sigma = 1$. Yellow cells follow Brownian motion, so the Brownian motion hypothesis should not be rejected and the estimated η value should be zero. Blue cells are log-ratios between one genus that follows Brownian motion and one genus that follows an OU process. For these cells, the Brownian motion hypothesis should be rejected and the estimated η should be smaller.

(a) log-proportion and CLR, estimated η

Genus	η	μ	log-prop	CLR
1	0		0.037	0.173
3	0		0.049	0.172
5	0		0.046	0.161
7	0		0.057	0.193
9	0		0.039	0.152
2	1	2	0.721	0.898
4	1	3	0.653	0.842
6	1	5	0.460	0.549
8	1	6	0.337	0.395
10	1	9	0.409	0.759

(b) Pairwise log ratio, estimated η

	3	5	7	9	2	4	6	8	10
1	0.007	0.007	0.008	0.008	0.625	0.547	0.404	0.298	0.243
3		0.008	0.008	0.008	0.613	0.543	0.396	0.304	0.229
5			0.007	0.008	0.581	0.504	0.359	0.282	0.21
7				0.009	0.611	0.549	0.413	0.31	0.238
9					0.574	0.501	0.367	0.278	0.197
2						1.005	1.007	1.004	1
4							1.006	1.015	1.004
6								1.028	1.001
8									1.003

(c) log-proportion and CLR, proportion that reject BM

Genus	η	μ	log-prop	CLR
1	0		0.40	0.90
3	0		0.45	0.91
5	0		0.40	0.88
7	0		0.43	0.86
9	0		0.45	0.88
2	1	2	1.00	1.00
4	1	3	1.00	1.00
6	1	5	0.99	1.00
8	1	6	0.95	0.97
10	1	9	0.93	1.00

(d) Pairwise log ratio, proportion that reject BM

	3	5	7	9	2	4	6	8	10
1	0.06	0.04	0.05	0.08	1	0.99	0.96	0.93	0.78
3		0.06	0.07	0.12	0.98	0.97	0.95	0.87	0.7
5			0.03	0.07	1	1	0.96	0.85	0.81
7				0.08	1	1	0.96	0.88	0.8
9					1	1	0.98	0.89	0.81
2						1	1	1	1
4							1	1	1
6								1	1
8									1

- For genera following Brownian motion, even if Brownian motion is rejected, the estimated mean reversion rates are very low, particularly for log-proportions.

- For genera following an OU process, mean reversion rates are underestimated by both CLR and log proportion, with log-proportion producing more underestimation.

- Pairwise log-ratios estimates the mean reversion rates very well in cases where both genera follow an OU process.

C.3.3 Scenario 3

Table 7 shows the average estimated $\hat{\eta}$ values for each genus over 100 simulations, under an OU process and the proportion of simulations which reject a null hypothesis of Brownian motion for various normalisation methods. The log-abundances of all genera are simulated following an OU process, but each genus has a different rate of mean reversion. We note the following features of these results:

- All methods reject Brownian motion for all genera.

- log-proportion tends to overestimate the mean-reversion rate for abundant genera, and underestimate the mean reversion rate for less abundant genera.

- CLR tends to overestimate η for less abundant genera and underestimate η for abundant genera. The error in estimating η is smaller for CLR than for log-proportion.

- Pairwise log-ratio gives reasonable estimates for η. There is not a meaningful true rate of mean reversion in this case, but the estimates mostly fall between the rates for the two genera.

C.3.4 Scenario 4

Table 8 shows the average estimated $\hat{\eta}$ values for each genus over 100 simulations, under an OU process and the proportion of simulations which reject a null hypothesis of i.i.d. samples for various normalisation methods. Half of the genera are simulated following a mean-reverting process with $\eta = 1$. The

Table 7: Comparison normalisation schemes: Scenario 3 without Poisson noise.
Results are averaged over 100 simulations. All genera follow OU processes with different rates of mean reversion. Initial abundances for each genus are i.i.d. log-normal with $\mu = 8$ and $\sigma = 1$.

(a) log-proportion and CLR, estimated η

Genus	η	μ	log-prop	CLR
1	0.6	6	0.514	0.556
2	0.7	9	1.088	0.767
3	0.8	6	0.681	0.810
4	0.9	9	1.351	0.882
5	1.0	6	0.832	1.073
6	1.1	9	1.792	1.056
7	1.2	6	1.037	1.344
8	1.3	9	2.135	1.166
9	1.4	6	1.129	1.621
10	1.5	9	2.442	1.296

(b) Pairwise log ratio, estimated η

	2	3	4	5	6	7	8	9	10
1	0.635	0.725	0.69	0.882	0.732	1.173	0.771	1.296	0.834
2		0.77	0.833	0.919	0.921	1.033	1.006	1.159	1.234
3			0.835	0.982	0.898	1.284	0.944	1.522	1.007
4				0.972	1.031	1.083	1.152	1.188	1.349
5					1.048	1.175	1.108	1.383	1.172
6						1.195	1.203	1.302	1.365
7							1.249	1.415	1.32
8								1.392	1.481
9									1.454

(c) log-proportion and CLR, proportion that reject BM

Genus	η	μ	log-prop	CLR
1	0.6	6	1	1
2	0.7	9	1	1
3	0.8	6	1	1
4	0.9	9	1	1
5	1.0	6	1	1
6	1.1	9	1	1
7	1.2	6	1	1
8	1.3	9	1	1
9	1.4	6	1	1
10	1.5	9	1	1

(d) Pairwise log ratio, proportion that reject BM

	2	3	4	5	6	7	8	9	10
1	1	1	1	1	1	1	1	1	1
2		1	1	1	1	1	1	1	1
3			1	1	1	1	1	1	1
4				1	1	1	1	1	1
5					1	1	1	1	1
6						1	1	1	1
7							1	1	1
8								1	1
9									1

Table 8: Comparison of normalisation schemes: Scenario 4 without Poisson noise

Results are averaged over 100 simulations. Initial abundances for each genus are i.i.d. log-normal with $\mu = 8$ and $\sigma = 1$. Red cells follow an i.i.d. normal distribution, so the i.i.d. hypothesis should not be rejected and the estimated η value should be large. Magenta cells are log-ratios between one genus that follows an i.i.d. normal distribution and one genus that follows an OU process. For these cells, the i.i.d. hypothesis should be rejected and the estimated η should be smaller.

(a) log-proportion and CLR, estimated η

Genus	η	log-prop	CLR
1	∞	4.703	4.233
3	∞	4.646	4.455
5	∞	4.481	4.257
7	∞	5.023	4.479
9	∞	4.668	4.131
2	1	1.489	1.455
4	1	1.600	1.705
6	1	2.123	2.976
8	1	2.753	3.848
10	1	3.755	2.076

(b) Pairwise log ratio, estimated η

	3	5	7	9	2	4	6	8	10
1	4.81	4.75	4.98	4.65	2.57	2.85	3.41	3.82	4.12
3		4.73	5.04	4.61	2.76	3.15	3.66	4.03	4.11
5			4.84	4.45	2.64	2.89	3.38	3.77	4.19
7				5.04	2.91	3.15	3.64	4.14	4.30
9					2.73	3.07	3.48	3.78	4.12
2						1.02	1.01	1.01	1.00
4							1.01	1.01	1.00
6								1.02	1.00
8									1.01

(c) log-proportion and CLR, proportion that reject iid

Genus	η	μ	log-prop	CLR
1	∞	2	0.14	0.48
3	∞	3	0.15	0.54
5	∞	4	0.14	0.46
7	∞	6	0.15	0.54
9	∞	8	0.30	0.45
2	1	2	1.00	1.00
4	1	3	1.00	1.00
6	1	5	0.95	0.89
8	1	6	0.85	0.68
10	1	9	0.81	1.00

(d) Pairwise log ratio, proportion that reject iid

	3	5	7	9	2	4	6	8	10
1	0	0.02	0	0.01	0.99	0.98	0.81	0.62	0.39
3		0.02	0	0.02	0.98	0.99	0.76	0.64	0.38
5			0	0	1	0.96	0.78	0.58	0.45
7				0	0.97	0.94	0.77	0.6	0.44
9					0.98	0.97	0.77	0.56	0.41
2						1	1	1	1
4							1	1	1
6								1	1
8									1

other half are simulated with $\eta = 2000$, effectively producing i.i.d. samples. We note the following features of these results:

- The false positive rate for pairwise log-ratios in cases where both genera are i.i.d. is close to or smaller than the nominal size of the test.
- Pairwise log-ratios have high power in cases where both genera follow OU processes, and reasonable power in cases where one genus follows an OU process and the other is i.i.d.
- The false positive rate for log-proportion is slightly higher than the nominal size, and increases for more abundant genera.
- The false positive rate for CLR is much higher than other methods.
- Both log-proportion and CLR have fairly high power.
- Both log-proportion and CLR overestimate the mean reversion rate. This overestimation is larger for more abundant genera.
- Pairwise log-ratios estimate the mean reversion rate well when both genera follow an OU process.

C.4 Summary of Simulation Results

- Without Poisson noise, pairwise log-ratios have false positive rates close to or below the nominal size of the test.
- With Poisson noise, all methods have false positive rates above the nominal size of the test. For pairwise log-ratios, the false positive rates are mainly in the range 10–35%, so not too large.
- For log-proportion, the false positive rate is in the range 10–30% for cases with Poisson noise and in the range 40–45% in the case without Poisson noise where some genera follow Brownian motion and others follow an OU process.
- CLR has much higher false positive rates than other methods.
- Even in cases where the tests give a false positive, the estimated mean reversion rate is very small.

- The mean reversion rate of one genus can affect the estimates for other genera using log-proportion or CLR.
- This effect is more significant for abundant genera using log-proportion and more significant for less abundant genera using CLR.

D Real Data Results

Tables 9 and 10 give the estimated mean reversion rates for the most abundant genera in each environment for the moving picture data. Tables 11 and 12 give the estimated $\hat{\sigma}$ for the most abundant genera in each environment for the moving picture data. Tables 13 and 14 give the estimated means $\hat{\mu}$ for the most abundant genera in each environment for the moving picture data. Table 15 gives the estimated mean reversion rates for the the most abundant genera in the gut for each individual in both the moving picture dataset and the David *et al.* dataset.

Table 9: Estimate $\hat{\eta}$ for abundant genera in the moving picture dataset.

					Person 1				Person 2			
Phylum	Class	Order	Family	Genus	gut	tongue	left	right	gut	tongue	left	right
Actinobacteria	Actinobacteria	Actinomycetales		.								1.6442
Actinobacteria	Actinobacteria	Actinomycetales	Actinomycetaceae	Actinomyces.		2.5106	1.1959			1.424	1.0559	1.9075
Actinobacteria	Actinobacteria	Actinomycetales	Actinomycetaceae	Varibaculum.					1.8519			1.0387
Actinobacteria	Actinobacteria	Actinomycetales	Brevibacteriaceae	Brevibacterium.								1.2825
Actinobacteria	Actinobacteria	Actinomycetales	Corynebacteriaceae	.			1.4795				2.47	2.0379
Actinobacteria	Actinobacteria	Actinomycetales	Corynebacteriaceae	Corynebacterium.			2.2379	4.2108			1.8968	1.7227
Actinobacteria	Actinobacteria	Actinomycetales	Dermabacteraceae	Dermabacter.								1.2937
Actinobacteria	Actinobacteria	Actinomycetales	Intrasporangiaceae	.								2.0855
Actinobacteria	Actinobacteria	Actinomycetales	Microbacteriaceae	.			2.204					1.4044
Actinobacteria	Actinobacteria	Actinomycetales	Micrococcaceae	.								2.3931
Actinobacteria	Actinobacteria	Actinomycetales	Micrococcaceae	Arthrobacter.			1.8153				1.2351	1.4337
Actinobacteria	Actinobacteria	Actinomycetales	Micrococcaceae	Kocuria.								2.2503
Actinobacteria	Actinobacteria	Actinomycetales	Micrococcaceae	Rothia.		1.7382	1.7072			1.1781	1.6843	3.0039
Actinobacteria	Actinobacteria	Actinomycetales	Nocardiaceae	Rhodococcus.								1.2362
Actinobacteria	Actinobacteria	Actinomycetales	Nocardioidaceae	.								1.572
Actinobacteria	Actinobacteria	Actinomycetales	Propionibacteriaceae	Propionibacterium.							2.4802	1.5844
Actinobacteria	Actinobacteria	Coriobacteriales	Coriobacteriaceae	Atopobium.						0.8266		
Bacteroidetes	Bacteroidia	Bacteroidales		.	0.8238				2.4357			
Bacteroidetes	Bacteroidia	Bacteroidales	Bacteroidaceae	Bacteroides.	2.5214		1.4978		6			2.0655
Bacteroidetes	Bacteroidia	Bacteroidales	Porphyromonadaceae	Odoribacter.					0.2851			
Bacteroidetes	Bacteroidia	Bacteroidales	Porphyromonadaceae	Parabacteroides.					0.7917			
Bacteroidetes	Bacteroidia	Bacteroidales	Porphyromonadaceae	Porphyromonas.		0.5899	0.982		1.7185	1.2128	1.9502	2.7479
Bacteroidetes	Bacteroidia	Bacteroidales	Prevotellaceae	Prevotella.		1.3354	1.0539	1.9036	1.4192	1.4471	2.2253	1.7535
Bacteroidetes	Bacteroidia	Bacteroidales	Rikenellaceae	Alistipes.					1.6207			
Bacteroidetes	Flavobacteria	Flavobacteriales	Flavobacteriaceae	.						1.2482		2.0539
Bacteroidetes	Flavobacteria	Flavobacteriales	Flavobacteriaceae	Capnocytophaga.							1.4595	2.3705
Bacteroidetes	Flavobacteria	Flavobacteriales	Flavobacteriaceae	Flavobacterium.							1.0279	0.967
Bacteroidetes	Sphingobacteria	Sphingobacteriales	Flexibacteraceae	Flectobacillus.								0.5386
Bacteroidetes	Sphingobacteria	Sphingobacteriales	Flexibacteraceae	Hymenobacter.								1.3381
Bacteroidetes	Sphingobacteria	Sphingobacteriales	Sphingobacteriaceae	Pedobacter.								1.1341
Cyanobacteria				.			1.4736	2.2284			1.0658	1.0925
Firmicutes	Bacilli	Bacillales		Exiguobacterium.								1.6105
Firmicutes	Bacilli	Bacillales		Gemella.		2.0472	1.3846			1.6197	2.961	2.8366
Firmicutes	Bacilli	Bacillales	Bacillaceae	Bacillus.			1.0585				1.9397	3.06
Firmicutes	Bacilli	Bacillales	Staphylococcaceae	Salinicoccus.							1.6466	1.2179
Firmicutes	Bacilli	Bacillales	Staphylococcaceae	Staphylococcus.			2.597	6			1.9018	1.0536
Firmicutes	Bacilli	Lactobacillales	Aerococcaceae	Abiotrophia.								2.0086
Firmicutes	Bacilli	Lactobacillales	Carnobacteriaceae	Granulicatella.		1.355	1.2066			1.7362	2.5284	2.2695
Firmicutes	Bacilli	Lactobacillales	Lactobacillaceae	Lactobacillus.			1.974	2.4098			1.6543	1.7501
Firmicutes	Bacilli	Lactobacillales	Streptococcaceae	Lactococcus.			1.1366				1.95	1.916
Firmicutes	Bacilli	Lactobacillales	Streptococcaceae	Streptococcus.		0.9752	1.3926	2.2464	1.2405	2.2932	2.4145	2.2825
Firmicutes	Clostridia	Clostridiales		.	0.4677				0.4857			
Firmicutes	Clostridia	Clostridiales	Catabacteriaceae	.					0.1449			
Firmicutes	Clostridia	Clostridiales	Clostridiaceae	Clostridium.			1.3273					1.4922
Firmicutes	Clostridia	Clostridiales	FamilyXI	.					1.7791		1.1402	0.9959
Firmicutes	Clostridia	Clostridiales	FamilyXI	Anaerococcus.			2.3314		1.5771		2.1896	1.9423
Firmicutes	Clostridia	Clostridiales	FamilyXI	Finegoldia.			1.903		1.4869		1.8718	2.3053
Firmicutes	Clostridia	Clostridiales	FamilyXI	Peptoniphilus.			2.1694		1.5488		1.8509	1.9309
Firmicutes	Clostridia	Clostridiales	Lachnospiraceae	.	1.2176				2.1258	0.7949		2.259
Firmicutes	Clostridia	Clostridiales	Lachnospiraceae	Blautia.	1.4392				2.7268			1.7381
Firmicutes	Clostridia	Clostridiales	Lachnospiraceae	Clostridium.	1.3574				1.8556			
Firmicutes	Clostridia	Clostridiales	Lachnospiraceae	Coprococcus.	1.0891				2.008			
Firmicutes	Clostridia	Clostridiales	Lachnospiraceae	Eubacterium.					0.6677			
Firmicutes	Clostridia	Clostridiales	Lachnospiraceae	Lachnobacterium.					0.297			
Firmicutes	Clostridia	Clostridiales	Lachnospiraceae	Lachnospira.	3.2833				1.1228			
Firmicutes	Clostridia	Clostridiales	Lachnospiraceae	Oribacterium.						0.9181		
Firmicutes	Clostridia	Clostridiales	Lachnospiraceae	Roseburia.	1.7145				1.4758			
Firmicutes	Clostridia	Clostridiales	Lachnospiraceae	Ruminococcus.					1.4278			
Firmicutes	Clostridia	Clostridiales	Ruminococcaceae	.	2.4777				1.6933			
Firmicutes	Clostridia	Clostridiales	Ruminococcaceae	Clostridium.					1.2342			
Firmicutes	Clostridia	Clostridiales	Ruminococcaceae	Eubacterium.					1.6407			
Firmicutes	Clostridia	Clostridiales	Ruminococcaceae	Faecalibacterium.	2.4203				1.2732			
Firmicutes	Clostridia	Clostridiales	Ruminococcaceae	Oscillospira.	2.1789				2.1399			
Firmicutes	Clostridia	Clostridiales	Ruminococcaceae	Ruminococcus.					0.29			
Firmicutes	Clostridia	Clostridiales	Veillonellaceae	Dialister.					1.2542			2.4768
Firmicutes	Clostridia	Clostridiales	Veillonellaceae	Megamonas.			0.822					
Firmicutes	Clostridia	Clostridiales	Veillonellaceae	Phascolarctobacterium.	6				1.7944			
Firmicutes	Clostridia	Clostridiales	Veillonellaceae	Veillonella.		1.2826	1.1607			1.8134	1.6328	1.8312
Fusobacteria	Fusobacteria	Fusobacteriales	Fusobacteriaceae	Fusobacterium.		0.6637	0.6321	0.6053		0.4885	1.3104	1.7179
Fusobacteria	Fusobacteria	Fusobacteriales	Fusobacteriaceae	J2.29.								1.3004
Fusobacteria	Fusobacteria	Fusobacteriales	Fusobacteriaceae	Leptotrichia.						1.2103	1.4121	1.9804

Table 10: Estimate $\hat{\eta}$ for abundant genera in the moving picture dataset (cont.)

					Person 1				Person 2			
Phylum	Class	Order	Family	Genus	gut	tongue	left	right	gut	tongue	left	right
Proteobacteria	Alphaproteobacteria	Caulobacterales	Caulobacteraceae	Brevundimonas.								1.6504
Proteobacteria	Alphaproteobacteria	Rhizobiales	Methylobacteriaceae	Methylobacterium.								1.9707
Proteobacteria	Alphaproteobacteria	Rhodobacterales	Rhodobacteraceae	Paracoccus.								1.8774
Proteobacteria	Alphaproteobacteria	Sphingomonadales	Erythrobacteraceae	Erythromicrobium.								0.6884
Proteobacteria	Alphaproteobacteria	Sphingomonadales	Sphingomonadaceae	Blastomonas.								0.811
Proteobacteria	Alphaproteobacteria	Sphingomonadales	Sphingomonadaceae	Kaistobacter.								1.5511
Proteobacteria	Alphaproteobacteria	Sphingomonadales	Sphingomonadaceae	Sphingobium.								1.4494
Proteobacteria	Alphaproteobacteria	Sphingomonadales	Sphingomonadaceae	Sphingomonas.			6				1.4621	1.1159
Proteobacteria	Betaproteobacteria	Burkholderiales	Alcaligenaceae	.	0.6338				0.068			
Proteobacteria	Betaproteobacteria	Burkholderiales	Burkholderiaceae	Lautropia.						1.4016	1.5335	2.1853
Proteobacteria	Betaproteobacteria	Burkholderiales	Comamonadaceae	.								0.7261
Proteobacteria	Betaproteobacteria	Burkholderiales	Comamonadaceae	Brachymonas.								0.9601
Proteobacteria	Betaproteobacteria	Burkholderiales	Comamonadaceae	Comamonas.								0.7087
Proteobacteria	Betaproteobacteria	Burkholderiales	Comamonadaceae	Hylemonella.								1.2882
Proteobacteria	Betaproteobacteria	Burkholderiales	Oxalobacteraceae	.							1.0509	1.0497
Proteobacteria	Betaproteobacteria	Burkholderiales	Oxalobacteraceae	Janthinobacterium.								1.2169
Proteobacteria	Betaproteobacteria	Burkholderiales	Oxalobacteraceae	Massilia.			6				1.079	1.2714
Proteobacteria	Betaproteobacteria	Neisseriales	Neisseriaceae	.			1.7782			0.8931		3.4669
Proteobacteria	Betaproteobacteria	Neisseriales	Neisseriaceae	Conchiformibius.			2.7525					1.6505
Proteobacteria	Betaproteobacteria	Neisseriales	Neisseriaceae	Neisseria.		0.3888	1.5985			1.4754	1.4876	1.8279
Proteobacteria	Deltaproteobacteria	Desulfovibrionales	Desulfovibrionaceae	Bilophila.					0.7328			
Proteobacteria	Deltaproteobacteria	Desulfovibrionales	Desulfovibrionaceae	Desulfovibrio.	1.5205				1.2311			
Proteobacteria	Epsilonproteobacteria	Campylobacterales	Campylobacteraceae	Campylobacter.					1.7426	1.0044		3.6213
Proteobacteria	Gammaproteobacteria	Enterobacteriales	Enterobacteriaceae	.								1.0387
Proteobacteria	Gammaproteobacteria	Enterobacteriales	Enterobacteriaceae	Escherichia.	1.0673				0.8784			
Proteobacteria	Gammaproteobacteria	Enterobacteriales	Enterobacteriaceae	Pantoea.								1.0362
Proteobacteria	Gammaproteobacteria	Oceanospirillales	Pseudomonadaceae	Pseudomonas.			1.3225	1.4825			1.2595	1.2808
Proteobacteria	Gammaproteobacteria	Pasteurellales	Pasteurellaceae	.		1.2105	2.296	2.1419		1.2779	2.1635	3.6708
Proteobacteria	Gammaproteobacteria	Pasteurellales	Pasteurellaceae	Actinobacillus.						0.4027		
Proteobacteria	Gammaproteobacteria	Pasteurellales	Pasteurellaceae	Gallibacterium.						0.3802		
Proteobacteria	Gammaproteobacteria	Pasteurellales	Pasteurellaceae	Haemophilus.		1.2447	1.2604			1.6429	1.6823	1.9761
Proteobacteria	Gammaproteobacteria	Pseudomonadales	Moraxellaceae	Acinetobacter.			0.7946	1.1707			1.1458	1.1116
Proteobacteria	Gammaproteobacteria	Pseudomonadales	Moraxellaceae	Enhydrobacter.			0.707					1.6925
Proteobacteria	Gammaproteobacteria	Pseudomonadales	Moraxellaceae	Moraxella.			1.9418					1.5368
Proteobacteria	Gammaproteobacteria	Pseudomonadales	Moraxellaceae	Psychrobacter.			1.0116					1.0637
Proteobacteria	Gammaproteobacteria	Vibrionales	Vibrionaceae	Photobacterium.								1.3984
Proteobacteria	Gammaproteobacteria	Xanthomonadales	Xanthomonadaceae	.								2.1284
Tenericutes	Erysipelotrichi	Erysipelotrichales	Erysipelotrichaceae	Bulleidia.						1.0922		
Tenericutes	Erysipelotrichi	Erysipelotrichales	Erysipelotrichaceae	Clostridium.					0.7616			
Tenericutes	Mollicutes	Mycoplasmatales	Mycoplasmataceae	Mycoplasma.								1.0052
Verrucomicrobia	Verrucomicrobiae	Verrucomicrobiales	Verrucomicrobiaceae	Akkermansia.	0.8704				0.2008			

Table 11: Estimate $\hat{\sigma}$ for abundant genera in the moving picture dataset (cont.)

					Person 1				Person 2			
Phylum	Class	Order	Family	Genus	gut	tongue	left	right	gut	tongue	left	right
Actinobacteria	Actinobacteria.class.	Actinomycetales		.								11.5713
Actinobacteria	Actinobacteria.class.	Actinomycetales	Actinomycetaceae	Actinomyces.		7.3339	5.8337			5.3231	5.7648	9.1805
Actinobacteria	Actinobacteria.class.	Actinomycetales	Actinomycetaceae	Varibaculum.					18.0721			7.6536
Actinobacteria	Actinobacteria.class.	Actinomycetales	Brevibacteriaceae	Brevibacterium.								9.1561
Actinobacteria	Actinobacteria.class.	Actinomycetales	Corynebacteriaceae	.			7.1460				11.5745	10.0903
Actinobacteria	Actinobacteria.class.	Actinomycetales	Corynebacteriaceae	Corynebacterium.			5.3490	7.9450			4.9047	4.7124
Actinobacteria	Actinobacteria.class.	Actinomycetales	Dermabacteraceae	Dermabacter.								9.8613
Actinobacteria	Actinobacteria.class.	Actinomycetales	Intrasporangiaceae	.								13.6072
Actinobacteria	Actinobacteria.class.	Actinomycetales	Microbacteriaceae	.			10.4786					9.4308
Actinobacteria	Actinobacteria.class.	Actinomycetales	Micrococcaceae	.								14.4841
Actinobacteria	Actinobacteria.class.	Actinomycetales	Micrococcaceae	Arthrobacter.			7.9834				7.4695	8.3561
Actinobacteria	Actinobacteria.class.	Actinomycetales	Micrococcaceae	Kocuria.								13.5286
Actinobacteria	Actinobacteria.class.	Actinomycetales	Micrococcaceae	Rothia.		4.7963	8.1885			4.7040	8.2849	12.3755
Actinobacteria	Actinobacteria.class.	Actinomycetales	Nocardiaceae	Rhodococcus.								9.3824
Actinobacteria	Actinobacteria.class.	Actinomycetales	Nocardioidaceae	.								11.3708
Actinobacteria	Actinobacteria.class.	Actinomycetales	Propionibacteriaceae	Propionibacterium.							11.6594	8.8403
Actinobacteria	Actinobacteria.class.	Coriobacteriales	Coriobacteriaceae	Atopobium.						5.6878		
Bacteroidetes	Bacteroidia	Bacteroidales			3.4568				7.2939			
Bacteroidetes	Bacteroidia	Bacteroidales	Bacteroidaceae	Bacteroides.	1.4124		8.2452		4.2234			13.8059
Bacteroidetes	Bacteroidia	Bacteroidales	Porphyromonadaceae	Odoribacter.					2.4435			
Bacteroidetes	Bacteroidia	Bacteroidales	Porphyromonadaceae	Parabacteroides.					3.2531			
Bacteroidetes	Bacteroidia	Bacteroidales	Porphyromonadaceae	Porphyromonas.		3.4054	4.7097		14.2417	4.1221	9.4673	12.0866
Bacteroidetes	Bacteroidia	Bacteroidales	Prevotellaceae	Prevotella.		2.8001	4.3239	7.2767	11.7634	3.0897	8.7249	7.5680
Bacteroidetes	Bacteroidia	Bacteroidales	Rikenellaceae	Alistipes.					6.2186			
Bacteroidetes	Flavobacteria	Flavobacteriales	Flavobacteriaceae	.						8.0703		11.9459
Bacteroidetes	Flavobacteria	Flavobacteriales	Flavobacteriaceae	Capnocytophaga.							10.0865	14.4215
Bacteroidetes	Flavobacteria	Flavobacteriales	Flavobacteriaceae	Flavobacterium.							7.9244	7.6471
Bacteroidetes	Sphingobacteria	Sphingobacteriales	Flexibacteraceae	Flectobacillus.								6.0873
Bacteroidetes	Sphingobacteria	Sphingobacteriales	Flexibacteraceae	Hymenobacter.								9.9119
Bacteroidetes	Sphingobacteria	Sphingobacteriales	Sphingobacteriaceae	Pedobacter.								8.5701
Cyanobacteria				.			4.7064	6.7833			4.2022	4.3862
Firmicutes	Bacilli	Bacillales		Exiguobacterium.								12.7964
Firmicutes	Bacilli	Bacillales		Gemella.		7.4695	6.6294			7.6401	12.3815	12.1848
Firmicutes	Bacilli	Bacillales	Bacillaceae	Bacillus.			5.6366				10.4153	14.5897
Firmicutes	Bacilli	Bacillales	Staphylococcaceae	Salinicoccus.							8.9410	7.2233
Firmicutes	Bacilli	Bacillales	Staphylococcaceae	Staphylococcus.			6.3380	10.1238			4.8409	3.1336
Firmicutes	Bacilli	Lactobacillales	Aerococcaceae	Abiotrophia.								11.5262
Firmicutes	Bacilli	Lactobacillales	Carnobacteriaceae	Granulicatella.		5.0272	6.8558			6.6148	11.4936	10.7642
Firmicutes	Bacilli	Lactobacillales	Lactobacillaceae	Lactobacillus.			7.6583	9.0704			8.8016	9.3972
Firmicutes	Bacilli	Lactobacillales	Streptococcaceae	Lactococcus.			7.4121				12.5400	12.3341
Firmicutes	Bacilli	Lactobacillales	Streptococcaceae	Streptococcus.		1.6824	3.7654	5.5317	10.3785	4.1578	5.8117	5.7289
Firmicutes	Clostridia	Clostridiales			2.8876				2.6672			
Firmicutes	Clostridia	Clostridiales	Catabacteriaceae	.					1.9824			
Firmicutes	Clostridia	Clostridiales	Clostridiaceae	Clostridium.			6.6831					9.9343
Firmicutes	Clostridia	Clostridiales	FamilyXI	.					15.1291		7.1054	6.4036
Firmicutes	Clostridia	Clostridiales	FamilyXI	Anaerococcus.			9.3642		15.05775		9.0037	8.3598
Firmicutes	Clostridia	Clostridiales	FamilyXI	Finegoldia.			9.5807		14.3401		8.5894	10.0665
Firmicutes	Clostridia	Clostridiales	FamilyXI	Peptoniphilus.			10.2840		14.1150		8.5189	8.9110
Firmicutes	Clostridia	Clostridiales	Lachnospiraceae		4.3996				6.4215	4.1473		13.3436
Firmicutes	Clostridia	Clostridiales	Lachnospiraceae	Blautia.	5.9901				10.9907			12.9068
Firmicutes	Clostridia	Clostridiales	Lachnospiraceae	Clostridium.	4.7992				7.1198			
Firmicutes	Clostridia	Clostridiales	Lachnospiraceae	Coprococcus.	5.1965				9.1480			
Firmicutes	Clostridia	Clostridiales	Lachnospiraceae	Eubacterium.					4.9234			
Firmicutes	Clostridia	Clostridiales	Lachnospiraceae	Lachnobacterium.					2.9662			
Firmicutes	Clostridia	Clostridiales	Lachnospiraceae	Lachnospira.	11.2117				5.3639			
Firmicutes	Clostridia	Clostridiales	Lachnospiraceae	Oribacterium.						4.8628		
Firmicutes	Clostridia	Clostridiales	Lachnospiraceae	Roseburia.	5.7201				5.6027			
Firmicutes	Clostridia	Clostridiales	Lachnospiraceae	Ruminococcus.					8.7554			
Firmicutes	Clostridia	Clostridiales	Ruminococcaceae	.	9.5727				5.4384			
Firmicutes	Clostridia	Clostridiales	Ruminococcaceae	Clostridium.					8.4540			
Firmicutes	Clostridia	Clostridiales	Ruminococcaceae	Eubacterium.					8.3283			
Firmicutes	Clostridia	Clostridiales	Ruminococcaceae	Faecalibacterium.	8.0139				4.2510			
Firmicutes	Clostridia	Clostridiales	Ruminococcaceae	Oscillospira.	11.8092				9.3243			
Firmicutes	Clostridia	Clostridiales	Ruminococcaceae	Ruminococcus.					2.0550			
Firmicutes	Clostridia	Clostridiales	Veillonellaceae	Dialister.					12.01442			15.3434
Firmicutes	Clostridia	Clostridiales	Veillonellaceae	Megamonas.			5.9172					
Firmicutes	Clostridia	Clostridiales	Veillonellaceae	Phascolarctobacterium.	12.8094				7.4434			
Firmicutes	Clostridia	Clostridiales	Veillonellaceae	Veillonella.		3.0536	6.3528			4.7209	8.4390	9.4384
Fusobacteria	Fusobacteria.class.	Fusobacteriales	Fusobacteriaceae	Fusobacterium.		2.7310	3.0524	3.0842		1.9958	6.9229	8.5149
Fusobacteria	Fusobacteria.class.	Fusobacteriales	Fusobacteriaceae	J2.29.								11.1478
Fusobacteria	Fusobacteria.class.	Fusobacteriales	Fusobacteriaceae	Leptotrichia.						5.3121	9.3729	12.2202

Table 12: Estimate $\hat{\sigma}$ for abundant genera in the moving picture dataset (cont.)

					Person 1				Person 2			
Phylum	Class	Order	Family	Genus	gut	tongue	left	right	gut	tongue	left	right
Proteobacteria	Alphaproteobacteria	Caulobacterales	Caulobacteraceae	Brevundimonas.								12.0270
Proteobacteria	Alphaproteobacteria	Rhizobiales	Methylobacteriaceae	Methylobacterium.								12.5906
Proteobacteria	Alphaproteobacteria	Rhodobacterales	Rhodobacteraceae	Paracoccus.								12.1347
Proteobacteria	Alphaproteobacteria	Sphingomonadales	Erythrobacteraceae	Erythromicrobium.								7.2840
Proteobacteria	Alphaproteobacteria	Sphingomonadales	Sphingomonadaceae	Blastomonas.								7.8584
Proteobacteria	Alphaproteobacteria	Sphingomonadales	Sphingomonadaceae	Kaistobacter.								10.9640
Proteobacteria	Alphaproteobacteria	Sphingomonadales	Sphingomonadaceae	Sphingobium.								11.8405
Proteobacteria	Alphaproteobacteria	Sphingomonadales	Sphingomonadaceae	Sphingomonas.			17.0637				8.4409	6.9039
Proteobacteria	Betaproteobacteria	Burkholderiales	Alcaligenaceae		5.4504				1.1395			
Proteobacteria	Betaproteobacteria	Burkholderiales	Burkholderiaceae	Lautropia.						10.0108	10.1252	12.9543
Proteobacteria	Betaproteobacteria	Burkholderiales	Comamonadaceae	.								5.4047
Proteobacteria	Betaproteobacteria	Burkholderiales	Comamonadaceae	Brachymonas.								8.24881
Proteobacteria	Betaproteobacteria	Burkholderiales	Comamonadaceae	Comamonas.								6.3986
Proteobacteria	Betaproteobacteria	Burkholderiales	Comamonadaceae	Hylemonella.								10.7243
Proteobacteria	Betaproteobacteria	Burkholderiales	Oxalobacteraceae	.							7.6925	7.6710
Proteobacteria	Betaproteobacteria	Burkholderiales	Oxalobacteraceae	Janthinobacterium.								10.3997
Proteobacteria	Betaproteobacteria	Burkholderiales	Oxalobacteraceae	Massilia.			18.6043				7.1110	8.1626
Proteobacteria	Betaproteobacteria	Neisseriales	Neisseriaceae	.			8.6421			5.0516		16.1174
Proteobacteria	Betaproteobacteria	Neisseriales	Neisseriaceae	Conchiformibius.			12.0884					11.3971
Proteobacteria	Betaproteobacteria	Neisseriales	Neisseriaceae	Neisseria.		1.4654	7.6738			2.7904	6.8382	8.1728
Proteobacteria	Deltaproteobacteria	Desulfovibrionales	Desulfovibrionaceae	Bilophila.					4.9589			
Proteobacteria	Deltaproteobacteria	Desulfovibrionales	Desulfovibrionaceae	Desulfovibrio.	7.4896				7.2623			
Proteobacteria	Epsilonproteobacteria	Campylobacterales	Campylobacteraceae	Campylobacter.					15.6096	5.4818		19.5163
Proteobacteria	Gammaproteobacteria	Enterobacteriales	Enterobacteriaceae	.								7.1132
Proteobacteria	Gammaproteobacteria	Enterobacteriales	Enterobacteriaceae	Escherichia.	8.2425				7.6737			
Proteobacteria	Gammaproteobacteria	Enterobacteriales	Enterobacteriaceae	Pantoea.								7.6438
Proteobacteria	Gammaproteobacteria	Oceanospirillales	Pseudomonadaceae	Pseudomonas.			4.6808	5.3423			5.3179	5.4727
Proteobacteria	Gammaproteobacteria	Pasteurellales	Pasteurellaceae	.		3.2254	7.7728	7.6217		3.2537	9.3398	12.7512
Proteobacteria	Gammaproteobacteria	Pasteurellales	Pasteurellaceae	Actinobacillus.						3.7028		
Proteobacteria	Gammaproteobacteria	Pasteurellales	Pasteurellaceae	Gallibacterium.						3.1286		
Proteobacteria	Gammaproteobacteria	Pasteurellales	Pasteurellaceae	Haemophilus.		4.07198	6.5562			4.5651	7.6930	8.9702
Proteobacteria	Gammaproteobacteria	Pseudomonadales	Moraxellaceae	Acinetobacter.			3.3955	4.9327			6.3454	6.1879
Proteobacteria	Gammaproteobacteria	Pseudomonadales	Moraxellaceae	Enhydrobacter.			4.9782					11.9601
Proteobacteria	Gammaproteobacteria	Pseudomonadales	Moraxellaceae	Moraxella.			8.9079					11.0640
Proteobacteria	Gammaproteobacteria	Pseudomonadales	Moraxellaceae	Psychrobacter.			5.5840					8.0841
Proteobacteria	Gammaproteobacteria	Vibrionales	Vibrionaceae	Photobacterium.								13.2585
Proteobacteria	Gammaproteobacteria	Xanthomonadales	Xanthomonadaceae	.								14.7030
Tenericutes	Erysipelotrichi	Erysipelotrichales	Erysipelotrichaceae	Bulleidia.						6.7680		
Tenericutes	Erysipelotrichi	Erysipelotrichales	Erysipelotrichaceae	Clostridium.					5.3507			
Tenericutes	Mollicutes	Mycoplasmatales	Mycoplasmataceae	Mycoplasma.								8.6197
Verrucomicrobia	Verrucomicrobiae	Verrucomicrobiales	Verrucomicrobiaceae	Akkermansia.	4.0206				2.2851			

Table 13: Estimate $\hat{\mu}$ for abundant genera in the moving picture dataset

					Person 1				Person 2			
Phylum	Class	Order	Family	Genus	gut	tongue	left	right	gut	tongue	left	right
Actinobacteria	Actinobacteria	Actinomycetales		.								−7.4205
Actinobacteria	Actinobacteria	Actinomycetales	Actinomycetaceae	Actinomyces.		−3.4808	−4.9175			−3.7949	−5.2548	−5.1891
Actinobacteria	Actinobacteria	Actinomycetales	Actinomycetaceae	Varibaculum.					−10.3265			−6.9604
Actinobacteria	Actinobacteria	Actinomycetales	Brevibacteriaceae	Brevibacterium.								−7.1794
Actinobacteria	Actinobacteria	Actinomycetales	Corynebacteriaceae	.			−4.9427				−5.4386	−5.4732
Actinobacteria	Actinobacteria	Actinomycetales	Corynebacteriaceae	Corynebacterium.			−2.6739	−2.6106			−2.5644	−2.6630
Actinobacteria	Actinobacteria	Actinomycetales	Dermabacteraceae	Dermabacter.								−7.5886
Actinobacteria	Actinobacteria	Actinomycetales	Intrasporangiaceae									−7.2990
Actinobacteria	Actinobacteria	Actinomycetales	Microbacteriaceae	.			−5.4108					−6.8131
Actinobacteria	Actinobacteria	Actinomycetales	Micrococcaceae	.								−7.0529
Actinobacteria	Actinobacteria	Actinomycetales	Micrococcaceae	Arthrobacter.			−4.7702				−5.9000	−5.8607
Actinobacteria	Actinobacteria	Actinomycetales	Micrococcaceae	Kocuria.								−6.8780
Actinobacteria	Actinobacteria	Actinomycetales	Micrococcaceae	Rothia.		−2.9299	−5.1490			−3.8693	−5.1586	−5.1481
Actinobacteria	Actinobacteria	Actinomycetales	Nocardiaceae	Rhodococcus.								−7.5191
Actinobacteria	Actinobacteria	Actinomycetales	Nocardioidaceae	.								−7.5227
Actinobacteria	Actinobacteria	Actinomycetales	Propionibacteriaceae	Propionibacterium.							−5.4510	−5.7885
Actinobacteria	Actinobacteria	Coriobacteriales	Coriobacteriaceae	Atopobium.						−6.6396		
Bacteroidetes	Bacteroidia	Bacteroidales		.	−3.8875				−3.4161			
Bacteroidetes	Bacteroidia	Bacteroidales	Bacteroidaceae	Bacteroides	−0.63912		−5.7097		−0.8806			−7.3766
Bacteroidetes	Bacteroidia	Bacteroidales	Porphyromonadaceae	Odoribacter.					−6.6474			
Bacteroidetes	Bacteroidia	Bacteroidales	Porphyromonadaceae	Parabacteroides.					−3.4558			
Bacteroidetes	Bacteroidia	Bacteroidales	Porphyromonadaceae	Porphyromonas.		−4.6240	−4.7009		−8.3256	−3.2954	−5.3135	−5.3396
Bacteroidetes	Bacteroidia	Bacteroidales	Prevotellaceae	Prevotella.		−2.0124	−3.9767	−4.1462	−7.6966	−1.9942	−4.3937	−4.5214
Bacteroidetes	Bacteroidia	Bacteroidales	Rikenellaceae	Alistipes.					−3.8723			
Bacteroidetes	Flavobacteria	Flavobacteriales	Flavobacteriaceae	.						−6.6031		−6.4910
Bacteroidetes	Flavobacteria	Flavobacteriales	Flavobacteriaceae	Capnocytophaga.							−7.0994	−7.0249
Bacteroidetes	Flavobacteria	Flavobacteriales	Flavobacteriaceae	Flavobacterium.							−7.3166	−7.4561
Bacteroidetes	Sphingobacteria	Sphingobacteriales	Flexibacteraceae	Flectobacillus.								−8.6899
Bacteroidetes	Sphingobacteria	Sphingobacteriales	Flexibacteraceae	Hymenobacter.								−7.4676
Bacteroidetes	Sphingobacteria	Sphingobacteriales	Sphingobacteriaceae	Pedobacter.								−7.3270
Cyanobacteria				.			−3.1275	−3.3252			−3.1455	−3.2411
Firmicutes	Bacilli	Bacillales		Exiguobacterium.								−8.3156
Firmicutes	Bacilli	Bacillales		Gemella.		−4.0966	−4.9340			−5.0316	−5.2293	−5.2693
Firmicutes	Bacilli	Bacillales	Bacillaceae	Bacillus.			−5.2742				−5.9000	−6.0531
Firmicutes	Bacilli	Bacillales	Staphylococcaceae	Salinicoccus.							−5.6971	−5.9236
Firmicutes	Bacilli	Bacillales	Staphylococcaceae	Staphylococcus.			−2.8868	−2.7839			−2.4898	−2.6003
Firmicutes	Bacilli	Lactobacillales	Aerococcaceae	Abiotrophia.								−6.3098
Firmicutes	Bacilli	Lactobacillales	Carnobacteriaceae	Granulicatella.		−3.8127	−5.6771			−4.0936	−5.3387	−5.4018
Firmicutes	Bacilli	Lactobacillales	Lactobacillaceae	Lactobacillus.			−4.2949	−4.3667			−5.3774	−5.5664
Firmicutes	Bacilli	Lactobacillales	Streptococcaceae	Lactococcus.			−6.2727				−6.8640	−6.8446
Firmicutes	Bacilli	Lactobacillales	Streptococcaceae	Streptococcus.		−1.5592	−2.7163	−2.7797	−8.3069	−2.0203	−2.6695	−2.7212
Firmicutes	Clostridia	Clostridiales			−5.5005				−4.6809			
Firmicutes	Clostridia	Clostridiales	Catabacteriaceae	.					−9.0534			
Firmicutes	Clostridia	Clostridiales	Clostridiaceae	Clostridium.			−5.0433					−6.7493
Firmicutes	Clostridia	Clostridiales	FamilyXI	.					−8.6326		−6.0092	−6.0435
Firmicutes	Clostridia	Clostridiales	FamilyXI	Anaerococcus.			−4.6545		−9.5346		−4.5453	−4.6528
Firmicutes	Clostridia	Clostridiales	FamilyXI	Finegoldia.			−5.5137		−9.4219		−4.8525	−4.9155
Firmicutes	Clostridia	Clostridiales	FamilyXI	Peptoniphilus.			−5.3678		−8.8730		−4.8391	−4.9289
Firmicutes	Clostridia	Clostridiales	Lachnospiraceae		−3.5925				−3.3787	−5.0470		−6.7714
Firmicutes	Clostridia	Clostridiales	Lachnospiraceae	Blautia	−4.3313				−4.8786			−7.8708
Firmicutes	Clostridia	Clostridiales	Lachnospiraceae	Clostridium.	−3.6285				−4.1373			
Firmicutes	Clostridia	Clostridiales	Lachnospiraceae	Coprococcus	−4.6938				−5.0159			
Firmicutes	Clostridia	Clostridiales	Lachnospiraceae	Eubacterium.					−6.6131			
Firmicutes	Clostridia	Clostridiales	Lachnospiraceae	Lachnobacterium.					−8.0519			
Firmicutes	Clostridia	Clostridiales	Lachnospiraceae	Lachnospira	−4.0569				−4.4435			
Firmicutes	Clostridia	Clostridiales	Lachnospiraceae	Oribacterium.						−5.0917		
Firmicutes	Clostridia	Clostridiales	Lachnospiraceae	Roseburia	−3.5551				−3.7760			
Firmicutes	Clostridia	Clostridiales	Lachnospiraceae	Ruminococcus.					−6.2434			
Firmicutes	Clostridia	Clostridiales	Ruminococcaceae	.	−4.5985				−3.3283			
Firmicutes	Clostridia	Clostridiales	Ruminococcaceae	Clostridium.					−6.8383			
Firmicutes	Clostridia	Clostridiales	Ruminococcaceae	Eubacterium.					−5.2643			
Firmicutes	Clostridia	Clostridiales	Ruminococcaceae	Faecalibacterium	−3.4162				−3.1006			
Firmicutes	Clostridia	Clostridiales	Ruminococcaceae	Oscillospira	−5.8210				−4.8793			
Firmicutes	Clostridia	Clostridiales	Ruminococcaceae	Ruminococcus.					−4.5870			
Firmicutes	Clostridia	Clostridiales	Veillonellaceae	Dialister.					−9.0743			−7.2534
Firmicutes	Clostridia	Clostridiales	Veillonellaceae	Megamonas.			−6.6255					
Firmicutes	Clostridia	Clostridiales	Veillonellaceae	Phascolarctobacterium.	−3.6214				−4.4163			
Firmicutes	Clostridia	Clostridiales	Veillonellaceae	Veillonella.		−2.3858	−5.3956			−2.7873	−5.4021	−5.4853
Fusobacteria	Fusobacteria	Fusobacteriales	Fusobacteriaceae	Fusobacterium.		−3.6507	−4.2204	−4.3882		−3.3392	−5.2305	−5.1911
Fusobacteria	Fusobacteria	Fusobacteriales	Fusobacteriaceae	J2.29.								−8.4821
Fusobacteria	Fusobacteria	Fusobacteriales	Fusobacteriaceae	Leptotrichia.						−4.4188	−6.7398	−6.7175

Table 14: Estimate $\hat{\mu}$ for abundant genera in the moving picture dataset (cont.)

					Person 1				Person 2			
Phylum	Class	Order	Family	Genus	gut	tongue	left	right	gut	tongue	left	right
Proteobacteria	Alphaproteobacteria	Caulobacterales	Caulobacteraceae	Brevundimonas								−7.6944
Proteobacteria	Alphaproteobacteria	Rhizobiales	Methylobacteriaceae	Methylobacterium.								−7.0246
Proteobacteria	Alphaproteobacteria	Rhodobacterales	Rhodobacteraceae	Paracoccus.								−7.0338
Proteobacteria	Alphaproteobacteria	Sphingomonadales	Erythrobacteraceae	Erythromicrobium.								−9.0722
Proteobacteria	Alphaproteobacteria	Sphingomonadales	Sphingomonadaceae	Blastomonas.								−8.7187
Proteobacteria	Alphaproteobacteria	Sphingomonadales	Sphingomonadaceae	Kaistobacter.								−7.3534
Proteobacteria	Alphaproteobacteria	Sphingomonadales	Sphingomonadaceae	Sphingobium.								−8.3436
Proteobacteria	Alphaproteobacteria	Sphingomonadales	Sphingomonadaceae	Sphingomonas.			−4.8426				−5.8908	−6.0165
Proteobacteria	Betaproteobacteria	Burkholderiales	Alcaligenaceae		−6.1823				−6.4699			
Proteobacteria	Betaproteobacteria	Burkholderiales	Burkholderiaceae	Lautropia.						−7.3281	−6.7912	−6.6319
Proteobacteria	Betaproteobacteria	Burkholderiales	Comamonadaceae	.								−6.2502
Proteobacteria	Betaproteobacteria	Burkholderiales	Comamonadaceae	Brachymonas								−7.8482
Proteobacteria	Betaproteobacteria	Burkholderiales	Comamonadaceae	Comamonas								−7.6921
Proteobacteria	Betaproteobacteria	Burkholderiales	Comamonadaceae	Hylemonella								−8.3630
Proteobacteria	Betaproteobacteria	Burkholderiales	Oxalobacteraceae	.							−7.0108	−7.0576
Proteobacteria	Betaproteobacteria	Burkholderiales	Oxalobacteraceae	Janthinobacterium								−8.4198
Proteobacteria	Betaproteobacteria	Burkholderiales	Oxalobacteraceae	Massilia.			−5.3087				−6.3163	−6.3736
Proteobacteria	Betaproteobacteria	Neisseriales	Neisseriaceae	.			−5.2204			−5.4986		−6.1485
Proteobacteria	Betaproteobacteria	Neisseriales	Neisseriaceae	Conchiformibius			−5.3089					−7.0425
Proteobacteria	Betaproteobacteria	Neisseriales	Neisseriaceae	Neisseria.		−2.6472	−5.0714			−1.7608	−4.6234	−4.7182
Proteobacteria	Deltaproteobacteria	Desulfovibrionales	Desulfovibrionaceae	Bilophila					−6.1985			
Proteobacteria	Deltaproteobacteria	Desulfovibrionales	Desulfovibrionaceae	Desulfovibrio	−5.1536				−5.8687			
Proteobacteria	Epsilonproteobacteria	Campylobacterales	Campylobacteraceae	Campylobacter.					−9.2567	−5.3263		−7.3090
Proteobacteria	Gammaproteobacteria	Enterobacteriales	Enterobacteriaceae	.								−6.5585
Proteobacteria	Gammaproteobacteria	Enterobacteriales	Enterobacteriaceae	Escherichia.	−6.4105				−7.5263			
Proteobacteria	Gammaproteobacteria	Enterobacteriales	Enterobacteriaceae	Pantoea.								−6.9942
Proteobacteria	Gammaproteobacteria	Oceanospirillales	Pseudomonadaceae	Pseudomonas.			−3.4514	−3.5926			−3.8338	−3.8876
Proteobacteria	Gammaproteobacteria	Pasteurellales	Pasteurellaceae	.		−2.6020	−3.8525	−3.9384		−2.5165	−4.8095	−4.6606
Proteobacteria	Gammaproteobacteria	Pasteurellales	Pasteurellaceae	Actinobacillus						−8.0022		
Proteobacteria	Gammaproteobacteria	Pasteurellales	Pasteurellaceae	Gallibacterium						−6.9575		
Proteobacteria	Gammaproteobacteria	Pasteurellales	Pasteurellaceae	Haemophilus.		−3.2934	−5.2455			−2.9191	−4.7779	−4.9392
Proteobacteria	Gammaproteobacteria	Pseudomonadales	Moraxellaceae	Acinetobacter			−3.6311	−3.9249			−5.2777	−5.2447
Proteobacteria	Gammaproteobacteria	Pseudomonadales	Moraxellaceae	Enhydrobacter.			−6.2993					−7.4243
Proteobacteria	Gammaproteobacteria	Pseudomonadales	Moraxellaceae	Moraxella.			−4.9836					−7.2141
Proteobacteria	Gammaproteobacteria	Pseudomonadales	Moraxellaceae	Psychrobacter.			−5.2749					−7.2321
Proteobacteria	Gammaproteobacteria	Vibrionales	Vibrionaceae	Photobacterium.								−9.5496
Proteobacteria	Gammaproteobacteria	Xanthomonadales	Xanthomonadaceae									−7.7673
Tenericutes	Erysipelotrichi	Erysipelotrichales	Erysipelotrichaceae	Bulleidia.						−6.2239		
Tenericutes	Erysipelotrichi	Erysipelotrichales	Erysipelotrichaceae	Clostridium					−6.4086			
Tenericutes	Mollicutes	Mycoplasmatales	Mycoplasmataceae	Mycoplasma								−8.0342
Verrucomicrobia	Verrucomicrobiae	Verrucomicrobiales	Verrucomicrobiaceae	Akkermansia.	−3.1217				−6.7384			

Table 15: Estimate $\hat{\eta}$ for abundant gut microbes in the moving picture dataset and the David *et al.* dataset

					Moving Picture		David *et al.*	
Phylum	Class	Order	Family	Genus	Person 1	Person 2	Subject A	Subject B
Actinobacteria	Actinobacteria	Actinomycetales	Actinomycetaceae	Actinomyces			0.8320	
Actinobacteria	Actinobacteria	Actinomycetales	Actinomycetaceae	Varibaculum.		1.8519		
Actinobacteria	Actinobacteria	Bifidobacteriales	Bifidobacteriaceae	Bifidobacterium			0.3566	0.9358
Bacteroidetes	Bacteroidia	Bacteroidales	Bacteroidaceae	Bacteroides	2.5214	6	1.4410	0.6007
Bacteroidetes	Bacteroidia	Bacteroidales	Porphyromonadaceae	Odoribacter.		0.2851		
Bacteroidetes	Bacteroidia	Bacteroidales	Porphyromonadaceae	Parabacteroides.		0.7917		
Bacteroidetes	Bacteroidia	Bacteroidales	Porphyromonadaceae	Porphyromonas.		1.7185		
Bacteroidetes	Bacteroidia	Bacteroidales	Prevotellaceae	Prevotella.		1.4192	2.0256	
Bacteroidetes	Bacteroidia	Bacteroidales	Rikenellaceae	Alistipes.		1.6207		
Firmicutes	Bacilli	Lactobacillales	Carnobacteriaceae	Granulicatella.			1.6666	
Firmicutes	Bacilli	Lactobacillales	Streptococcaceae	Streptococcus.		1.2405	1.3472	1.3091
Firmicutes	Clostridia	Clostridiales	FamilyXI	Anaerococcus.		1.5771		
Firmicutes	Clostridia	Clostridiales	FamilyXI	Finegoldia.		1.4869		
Firmicutes	Clostridia	Clostridiales	FamilyXI	Peptoniphilus.		1.5488		
Firmicutes	Clostridia	Clostridiales	Lachnospiraceae	Blautia.	1.4392	2.7268	0.7966	1.0021
Firmicutes	Clostridia	Clostridiales	Lachnospiraceae	Clostridium.	1.3574	1.8556		
Firmicutes	Clostridia	Clostridiales	Lachnospiraceae	Coprococcus.	1.0891	2.008	0.7619	0.6375
Firmicutes	Clostridia	Clostridiales	Lachnospiraceae	Dorea			0.8493	0.7622
Firmicutes	Clostridia	Clostridiales	Lachnospiraceae	Eubacterium.		0.6677		
Firmicutes	Clostridia	Clostridiales	Lachnospiraceae	Lachnobacterium.		0.297		
Firmicutes	Clostridia	Clostridiales	Lachnospiraceae	Lachnospira.	3.2833	1.1228		
Firmicutes	Clostridia	Clostridiales	Lachnospiraceae	Roseburia.	1.7145	1.4758	0.1311	3.9203
Firmicutes	Clostridia	Clostridiales	Lachnospiraceae	Ruminococcus.		1.4278	0.5008	0.1390
Firmicutes	Clostridia	Clostridiales	Ruminococcaceae	Clostridium.		1.2342		
Firmicutes	Clostridia	Clostridiales	Ruminococcaceae	Eubacterium.		1.6407		
Firmicutes	Clostridia	Clostridiales	Ruminococcaceae	Faecalibacterium.	2.4203	1.2732	0.8978	0.7168
Firmicutes	Clostridia	Clostridiales	Ruminococcaceae	Oscillospira.	2.1789	2.1399		
Firmicutes	Clostridia	Clostridiales	Ruminococcaceae	Ruminococcus.		0.29	0.5539	0.2663
Firmicutes	Clostridia	Clostridiales	Veillonellaceae	Dialister.		1.2542		
Firmicutes	Clostridia	Clostridiales	Veillonellaceae	Phascolarctobacterium.	6	1.7944	0.7340	1.8373
Firmicutes	Clostridia	Clostridiales	Veillonellaceae	Veillonella.			0.9389	1.4537
Fusobacteria	Fusobacteria	Fusobacteriales	Fusobacteriaceae	Fusobacterium.			2.0122	
Proteobacteria	Deltaproteobacteria	Desulfovibrionales	Desulfovibrionaceae	Bilophila		0.7328		
Proteobacteria	Deltaproteobacteria	Desulfovibrionales	Desulfovibrionaceae	Desulfovibrio	1.5205	1.2311		
Proteobacteria	Epsilonproteobacteria	Campylobacterales	Campylobacteraceae	Campylobacter		1.7426		
Proteobacteria	Gammaproteobacteria	Enterobacteriales	Enterobacteriaceae	Escherichia.	1.0673	0.8784		
Proteobacteria	Gammaproteobacteria	Pasteurellales	Pasteurellaceae	Haemophilus.			2.1665	1.2358
Tenericutes	Erysipelotrichi	Erysipelotrichales	Erysipelotrichaceae	Clostridium.		0.7616		
Verrucomicrobia	Verrucomicrobiae	Verrucomicrobiales	Verrucomicrobiaceae	Akkermansia.	0.8704	0.2008		

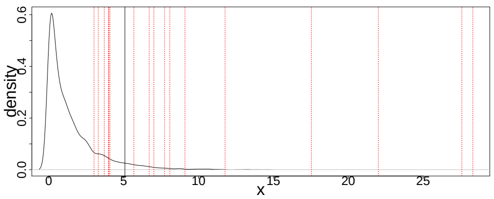

(a) Person 1 gut

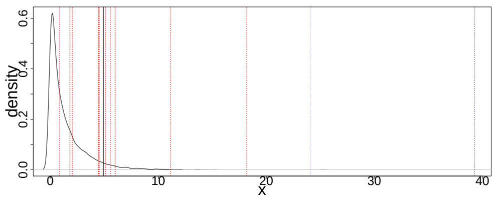

(b) Person 1 tongue

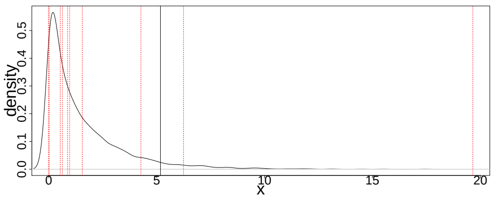

(c) Person 1 right palm

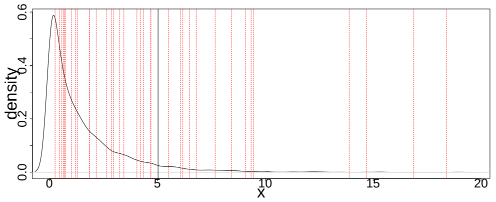

(d) Person 1 left palm

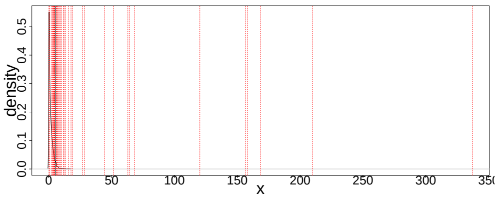

(e) Person 2 gut

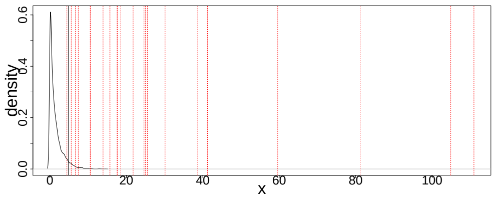

(f) Person 2 tongue

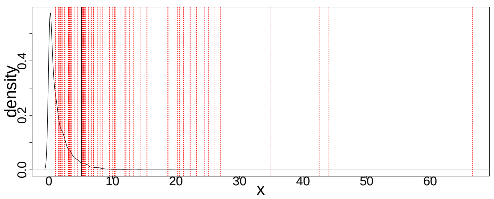

(g) Person 2 right palm

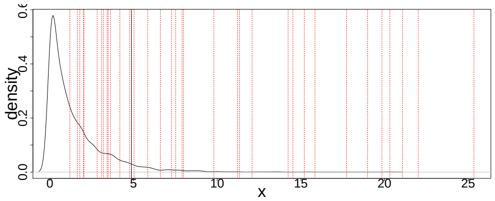

(h) Person 2 left palm

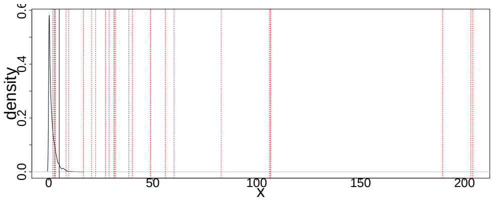

(i) David *et al.*, Subject A

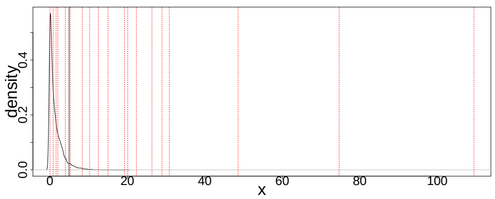

(j) David *et al.* Subject B

Figure 1: Null distribution density plots of Likelihood ratio statistic between i.i.d normal distribution and OU mean reverting process for the gut, tongue and palms genera in the moving picture dataset, and both subjects in the David *et al.* dataset, using log proportions. Vertical red lines indicate the likelihood ratios for the real data, while vertical black lines are the critical values at the 5% significance levels.

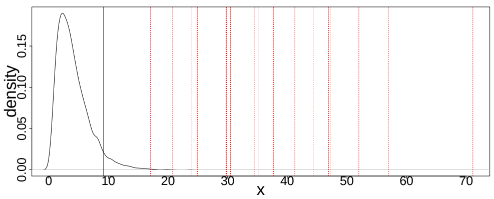

(a) Person 1 gut

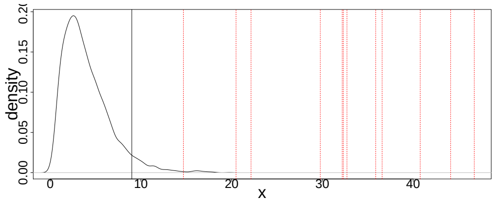

(b) Person 1 tongue

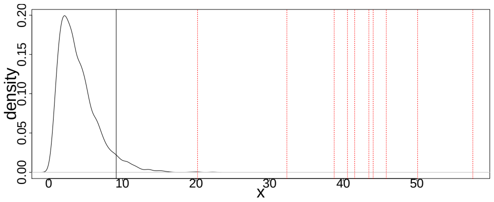

(c) Person 1 right palm

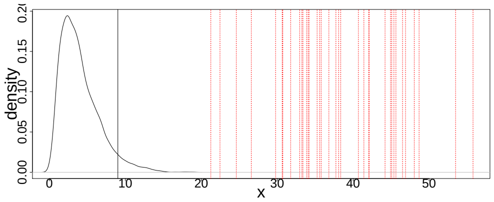

(d) Person 1 left palm

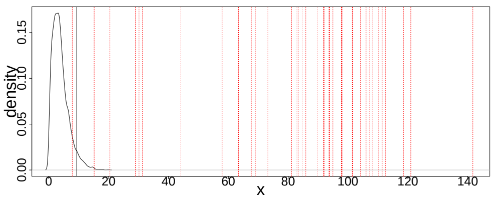

(e) Person 2 gut

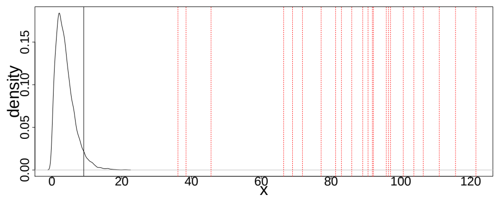

(f) Person 2 tongue

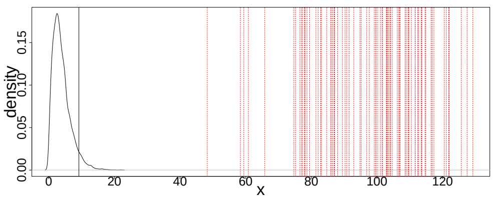

(g) Person 2 right palm

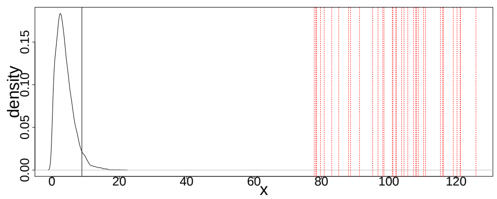

(h) Person 2 left palm

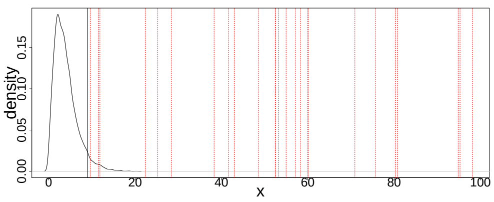

(i) David *et al.*, Subject A

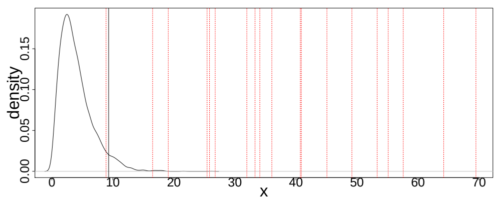

(j) David *et al.* Subject B

Figure 2: Null distribution density plots of Likelihood ratio statistic between Brownian motion and OU mean reverting process for the gut, tongue and palms genera in the moving picture dataset and both subjects in the David *et al.* dataset, using log proportion data. The black curve is the density of the likelihood ratio calculated from the standard likelihood formula for the OU process. The grey curve is the density of the likelihood ratio statistic calculated using a Taylor approximation. Vertical red lines indicate the likelihood ratios for the real data, while the vertical black line is the critical values at the 5% significance level.

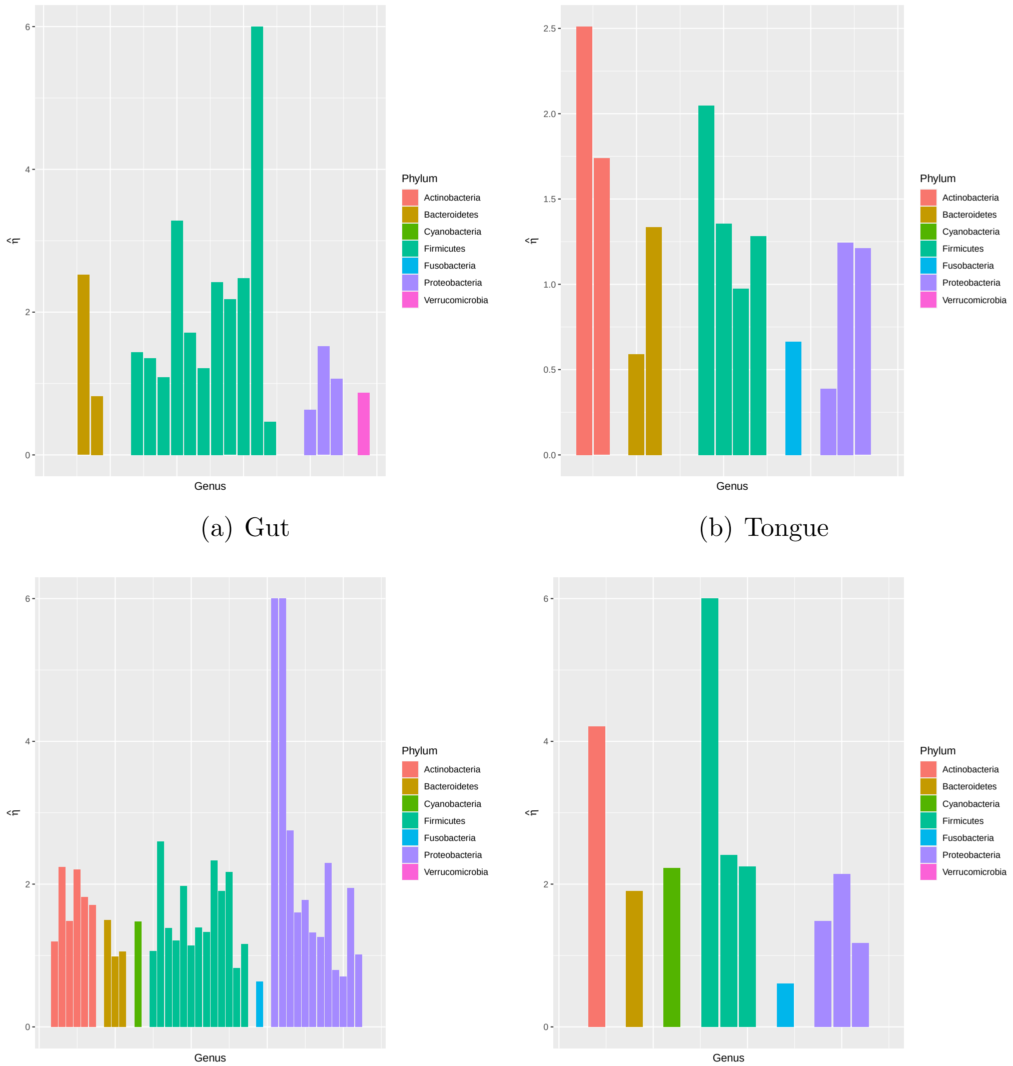

(a) Gut

(b) Tongue

(c) Left Palm

(d) Right Palm

Figure 3: Comparison of estimated η values for common genera at each body site for Person 1. The genera are arranged by taxonomy.
